# Synthesis of Nitro Compounds from Nitrogen Dioxide Captured in a Metal-Organic Framework

**DOI:** 10.1021/jacs.2c07283

**Published:** 2022-10-05

**Authors:** Jiangnan Li, Zi Wang, Yinlin Chen, Yongqiang Cheng, Luke L. Daemen, Floriana Tuna, Eric J. L. McInnes, Sarah J. Day, Anibal J. Ramirez-Cuesta, Martin Schröder, Sihai Yang

**Affiliations:** † Department of Chemistry, University of Manchester, Manchester M13 9PL, U.K.; ‡ Neutron Scattering Division, Neutron Sciences Directorate, Oak Ridge National Laboratory, Oak Ridge, Tennessee 37831, United States; § Photon Science Institute, University of Manchester, Manchester M13 9PL, U.K.; ∥ Diamond Light Source, Harwell Science Campus, Oxfordshire OX11 0DE, U.K.

## Abstract

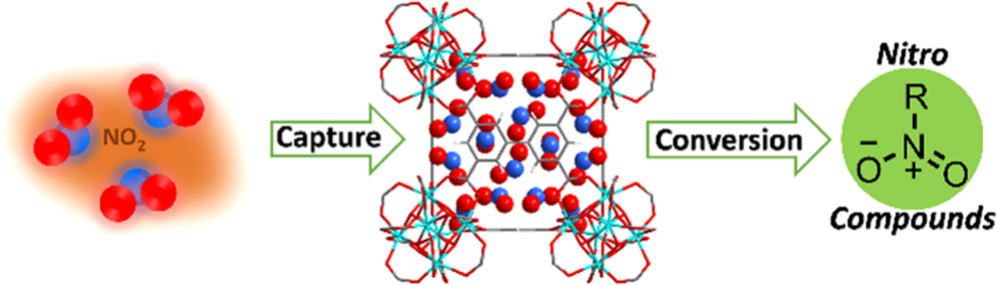

Increasing levels of air pollution are driving the need for the development of new processes that take “waste-to-chemicals”. Herein, we report the capture and conversion under ambient conditions of a major air pollutant, NO_2_, using a robust metal-organic framework (MOF) material, Zr-bptc (H_4_bptc = 3,3′,5,5′-biphenyltetracarboxylic acid), comprising {Zr_6_(μ_3_-O)_4_(μ_3_-OH)_4_(COO)_12_} clusters linked by 4-connected bptc^4–^ ligands in an **ftw** topology. At 298 K, Zr-bptc shows exceptional stability and adsorption of NO_2_ at both low (4.9 mmol g^–1^ at 10 mbar) and high pressures (13.8 mmol g^–1^ at 1.0 bar), as measured by isotherm experiments. Dynamic breakthrough experiments have confirmed the selective retention of NO_2_ by Zr-bptc at low concentrations under both dry and wet conditions. The immobilized NO_2_ can be readily transformed into valuable nitro compounds relevant to construction, agrochemical, and pharmaceutical industries. *In situ* crystallographic and spectroscopic studies reveal strong binding interactions of NO_2_ to the {Zr_6_(μ_3_-O)_4_(μ_3_-OH)_4_(COO)_12_} cluster node. This study paves a circular pathway to enable the integration of nitrogen-based air pollutants into the production of fine chemicals.

## Introduction

The growing emissions of nitrogen dioxide, NO_2_, from the combustion of fossil fuels contribute significantly to global warming, acid rain, and ozone depletion and have severe impacts on the environment and human health.^1−4^ State-of-the-art deNO*_x_* processes based upon selective catalytic reduction (SCR) incorporating precious metal catalysts, toxic chemicals, and significant energy consumption^5^ are struggling to meet increasingly stringent regulations.^6^ The transformation of waste into value-added chemicals is therefore becoming an important target in the development of “circular economy”, where products are made, used, and reused, rather than being disposed.^7^ Nitro compounds and their derivatives are important intermediates for a wide range of explosives, colorants, agrochemicals, and pharmaceuticals,^8,9^ but the state-of-the-art synthesis of nitro compounds relies heavily on the use of HNO_3_ and NH_3_ that are produced from the extremely energy-demanding Ostwald and Haber–Bosch processes, respectively.^10^ The capture and enrichment of pollutant NO_2_ and its conversion into nitro compounds are thus a promising route to achieve the circular utilization of reactive nitrogen resources, as well as reducing the carbon footprint for chemical industries.

Exploiting the high porosity and stability of porous materials for the reversible capture of target gases affords economically viable technologies for clean-up and mitigation of gaseous pollutants.^11^ Traditional porous materials, such as activated carbons,^12^ silica,^13^ and zeolites,^14,15^ have been tested for the adsorption of NO_2_. However, their limited structural stability and restricted design functionalization result in low and often irreversible adsorption. As emerging solid sorbents for a wide spectrum of gases and vapors,^16,17^ metal-organic framework (MOF) materials and their composites have been investigated for the adsorption of NO_2_.^18^ Although some systems have achieved high dynamic sorption (250–2138 ppm) in exceptional cases, the rapid structural degradation of the MOF host upon NO_2_ uptake has hampered their further applications (Table S1).^19−24^ To the best of our knowledge, only MFM-300(Al)^25^ and MFM-520^26^ have been reported to display fully reversible NO_2_ uptake for the pure gas over repeated cycles. However, MFM-300(Al) shows only a very low uptake of 1.4 mmol g^–1^ at low pressure (10 mbar and 298 K) owing to its inherently moderate binding sites (μ_2_-OH and aromatic C–H groups), while MFM-520 exhibits a low total uptake of 4.5 mmol g^–1^ (1.0 bar and 298 K) due to its limited porosity (surface area of 313 m^2^ g^–1^) (Figure S4). To enable the enrichment of NO_2_ within pores at low concentrations and the subsequent efficient conversion of NO_2_ to nitro compounds, the sorbent material must display high adsorption under both low and high pressures and afford sufficient stability upon regeneration. This represents an extremely challenging target.

Herein, we report the high adsorption of NO_2_ in a Zr-based MOF, Zr-bptc, which displays a fully reversible uptake of 4.9 and 13.8 mmol g^–1^ at 10 mbar and 1.0 bar, respectively, at 298 K. In addition, breakthrough experiments confirm that Zr-bptc exhibits highly selective retention of NO_2_ at low concentrations (2500 ppm) under both dry and wet conditions. Importantly, the immobilized NO_2_ molecules (4.9–13.8 mmol g^–1^) can be quantitatively converted to various nitro compounds under ambient conditions. The binding domains of NO_2_ (and also CO_2_ for comparison) in Zr-bptc have been determined by *in situ* synchrotron X-ray powder diffraction. The adsorbed NO_2_ molecules partially dimerize to N_2_O_4_ in the pore, and this has been studied by variable-temperature electron paramagnetic resonance (EPR) spectroscopy. The remaining NO_2_ monomers are stabilized by strong host–guest interactions with heats of adsorption (*Q*_st_) of 90 kJ mol^–1^, which have been visualized by *in situ* inelastic neutron scattering (INS) and EPR studies, coupled with density functional theory (DFT) calculations. More importantly, Zr-bptc can be fully regenerated upon the delivery of nitro compounds and reused, thus fulfilling the “waste-to-chemicals” target.

## Experimental Methods

### NO_2_ Adsorption Isotherms

Gravimetric sorption isotherms of NO_2_ were recorded at 298, 303, 308, and 313 K, maintained using a temperature-programmed water bath and furnace, on a Hiden Xemis system under ultrahigh vacuum (10^–10^ bar) using a turbo pumping system. Ultrapure research grade (99.999%) NO_2_ was purchased from BOC. In a typical gas adsorption experiment, acetone-exchanged Zr-bptc (50 mg) was loaded into the Xemis system and activated at 573 K under a dynamic high vacuum (10^–10^ bar) for 24 h to give fully desolvated Zr-bptc.

### Breakthrough Experiments

The flow rate of the entering gas mixture was maintained at 40 mL min^–1^, and the gas concentration, *C*, of gases at the outlet was determined by mass spectrometry and compared with the corresponding inlet concentration *C*_0_, where *C*/*C*_0_ = 1 indicates a complete breakthrough. For breakthrough separation under wet conditions, a fixed bed was packed with Zr-bptc that had been treated and preadsorbed with water at 75% RH. A gas mixture of 0.25% NO_2_ (2500 ppm diluted in 22.25% He and 77.5% N_2_) was then passed through the fixed bed. Breakthrough separation of NO_2_/CO_2_ was conducted using a mixture of 0.25% NO_2_ (2500 ppm) and 6.25% CO_2_ diluted in 93.5% He. The concentrations of NO_2_ and CO_2_ were chosen to mimic a typical exhaust gas of combustion of diesels in marine transport (N_2_: 77.50%, O_2_: 13.75%, CO_2_: 6.25%, NO_2_: 0.212%, SO_2_: 0.17%, H_2_O: 0.025%, CO: 0.005%, hydrocarbons 0.005%).^27^

### General Procedure for Conversion

Preactivated Zr-bptc (1.0 g) was packed in a fixed bed, and a gas flow of 2500 ppm NO_2_ (diluted in 77.5% N_2_ and 22.25% He) was passed through the column at 298 K until a complete breakthrough was achieved. The gas flow was switched off, and Zr-bptc loaded with 2500 ppm NO_2_ (denoted as NO_2_@Zr-bptc-N) was sealed for the study of conversion. The quantity of captured NO_2_ in NO_2_@Zr-bptc-N was determined by TGA, which shows a 19% weight loss between 25 and 330 °C, corresponding to an uptake of 5.1 mmol g^–1^ (Figure S23). This is consistent with that (4.9 mmol g^–1^) observed in the breakthrough experiment. Aromatic substrates (0.75 mmol) and CHCl_3_ (5.0 mL) were added to a 10 mL round-bottom flask and stirred for 5 min to obtain a clear solution. NO_2_@Zr-bptc-N containing 0.51 mmol NO_2_ was added to the solution under stirring at room temperature or 0 °C. Upon completion of the reaction, the mixture was centrifuged, the solid was recycled, and the supernatant was collected and reduced under vacuum for analysis. Nitration experiments were also conducted using Zr-bptc loaded with NO_2_ at 1 bar, 298 K (denoted as NO_2_@Zr-bptc-N*) (see SI 1.12). The quantity of captured NO_2_ in NO_2_@Zr-bptc-N* was determined by TGA, which shows a 40% weight loss between 25 and 330 °C, corresponding to an uptake of 14.3 mmol g^–1^ (Figure S23), consistent with that (13.8 mmol g^–1^) observed in the isotherm adsorption experiments.

## Results and Discussion

The highly stable MOF Zr-bptc [Zr_6_O_4_(OH)_4_(bptc)_3_] (H_4_bptc = biphenyl-3,3′,5,5′-tetracarboxylic acid) incorporates 12-connected {Zr_6_(μ_3_-O)_4_(μ_3_-OH)_4_(COO)_12_} clusters linked by 4-connected bptc^4–^ ligands to form an open and neutral framework in the **ftw** topology.^28^ Desolvated Zr-bptc exhibits cubic-shaped cages decorated with {Zr_6_} clusters and planar bptc^4–^ linkers on the vertices and faces, respectively (denoted as Cage A; Figure 2a). Cage A has a diameter of ∼12 Å after considering the van der Waals’ radii, and they are interconnected through another type of smaller tetrahedral cages of ∼4.5 Å diameter (Cage B; Figure 2a) located at the 12 edges of cubic Cage A. The ratio of Cage A to B is 1:3. Desolvated Zr-bptc shows a Brunauer–Emmett–Teller (BET) surface area of 960 m^2^ g^–1^, a pore volume of 0.413 g cm^–3^, and high chemical, thermal, and water/moisture stability (Figures S1 and S2).

**Figure 1:**
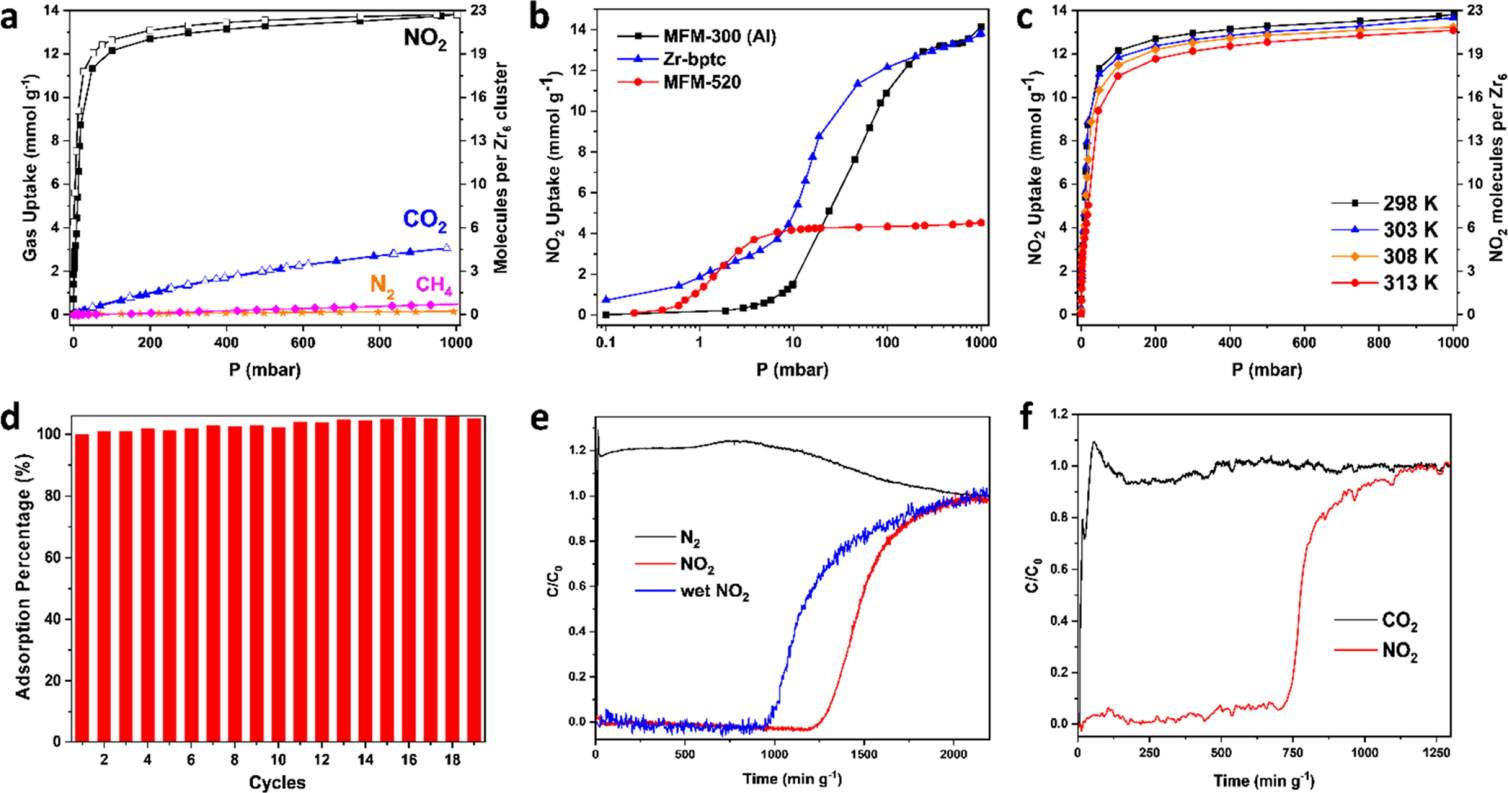
Gas adsorption and dynamic separation data. (a) Adsorption isotherms for NO_2_, CO_2_, N_2_, and CH_4_ in desolvated Zr-bptc at 298 K (desorption of N_2_ and CH_4_ is omitted for clarity); (b) comparison of NO_2_ adsorption isotherms on a logarithmic scale for Zr-bptc, MFM-300(Al), and MFM-520 (desorption data are omitted for clarity); (c) sorption isotherms for NO_2_ in Zr-bptc (desorption data are omitted for clarity and shown in Figure S5); (d) cyclic adsorption–desorption of NO_2_ at 298 K between 0 and 200 mbar (a small gradual increase in the uptake was due to the minor amount of retained NO_2_ in Zr-bptc upon desorption under pressure-swing conditions); (e) breakthrough plots for N_2_/NO_2_ gas mixtures under dry and wet conditions (2500 ppm NO_2_ diluted in 77.5% N_2_ and 22.25% He); and (f) breakthrough plots for CO_2_/NO_2_ gas mixtures (2500 ppm NO_2_ and 6.25% CO_2_ diluted in 93.5% He).

Isothermal adsorption of NO_2_ in Zr-bptc reveals exceptional uptakes of 1.8, 4.9, and 13.8 mmol g^–1^ at 0.001, 0.01, and 1.0 bar, respectively, at 298 K (Figures 1a–c and S5). The uptake of NO_2_ at very low pressure by Zr-bptc exceeds that of the state-of-the-art material, MFM-520 (1.3 and 4.2 mmol g^–1^ at 0.001 and 0.01 bar, respectively, at 298 K), thus representing a new benchmark for NO_2_ adsorption and confirming its potential for the efficient capture of NO_2_ at low concentrations. In addition, significant amounts of residual NO_2_ (30–50%) were observed upon pressure-swing desorption (Figures 1a and S5), indicating the strong binding of NO_2_ in Zr-bptc, which acts as an efficient NO_2_ store. Zr-bptc displays a comparable uptake capacity at 1.0 bar with the best-performing material, MFM-300(Al) (14.1 mmol g^–1^ at 298 K),^25^ and this high total uptake translates to high working capacities for the storage and conversion of NO_2_. Thus, the exceptional uptakes at both very low and ambient pressures endow Zr-bptc with great potential for the development of “waste-to-chemicals” processes. Although a number of metal-doped MOFs^20,24,29^ and MOF/graphite oxide composites^23^ show the dynamic adsorption of NO_2_ (*ca*. 500–1000 ppm) from gas mixtures under both dry and humid conditions, this is not comparable with the direct uptakes obtained *via* isotherm experiments with pure NO_2_ owing to the uncertainties in the former, which has been discussed in detail in our earlier report.^25^ Furthermore, these composite materials often display partial or complete structural degradation on NO_2_ adsorption–desorption cycles. In contrast, little change in structure or sorption capacity was observed for Zr-bptc after 19 cycles of adsorption and desorption of NO_2_ (Figures 1d, S1, and S2). Combined thermogravimetric analysis (TGA) and differential scanning calorimetry (DSC) determined the heats of adsorption for NO_2_ uptake in Zr-bptc to be 90 kJ mol^–1^ (Figure S9), substantially higher than for MFM-300(Al)^25^ and MFM-520(Zn)^26^ (61 and 58 kJ mol^–1^, respectively, determined by TGA–DSC; Figures S10 and S11), thus suggesting the presence of stronger interactions between Zr-bptc and adsorbed NO_2_ molecules. This is also consistent with the higher uptakes observed for Zr-bptc at both low and high pressures. Significantly, Zr-bptc shows low adsorption of CO_2_, CH_4_, and N_2_ at 298 K and 1.0 bar (3.05, 0.49, and 0.14 mmol g^–1^, respectively) (Figures 1a and S3). The value of *Q*_*st*_ for CO_2_ uptake is 28 kJ mol^–1^ and shows little variation as a function of surface coverage, indicating weak host–guest interactions (Figures S12 and S13 and Table S2).

Analysis of pure-component isotherms *via* ideal adsorbed solution theory (IAST)^30^ affords adsorption selectivities for various gas mixtures (N_2_, CO_2_, and NO_2_) at 298 K and 0–1.0 bar (Figure S14). The IAST selectivities for NO_2_/CO_2_ (1:99) and NO_2_/N_2_ (1:99) are ∼750 and >5000, respectively. It is worth noting that the very high IAST selectivities are subject to uncertainties owing to the extremely low adsorption of N_2_ or overlapping binding sites of NO_2_ and CO_2_ in Zr-bptc but confirm the potential of Zr-bptc in the selective adsorption of NO_2_ in the presence of CO_2_ and N_2_. Dynamic breakthrough experiments using a fixed bed packed with Zr-bptc were undertaken with gas mixtures of NO_2_/N_2_ (2500 ppm NO_2_ diluted in 77.5% N_2_ and 22.25% He) under both dry and wet (relative humidity: 75%) conditions and with NO_2_/CO_2_ (2500 ppm NO_2_ and 6.25% CO_2_ diluted in 93.5% He) at 298 K and 1.0 bar.^27^ Highly selective retention of NO_2_ by Zr-bptc was observed in all cases, showing a retention time of NO_2_ of 1200, 980, and 750 min g^–1^ for the mixture of NO_2_/N_2_ (dry), NO_2_/N_2_ (wet), and NO_2_/CO_2_, respectively (Figure 1e,f) (additional data are shown in Figures S6–S8). Compared with the dynamic adsorption capacity of NO_2_ (4.9 mmol g^–1^) under dry conditions, the small reduction (4.0 mmol g^–1^) under wet conditions is due to the competitive adsorption of water and reaction between adsorbed NO_2_ and H_2_O molecules in the pore. The dynamic selectivities of NO_2_/N_2_ and NO_2_/CO_2_ are estimated to be 151 and 24, respectively.

**Figure 2:**
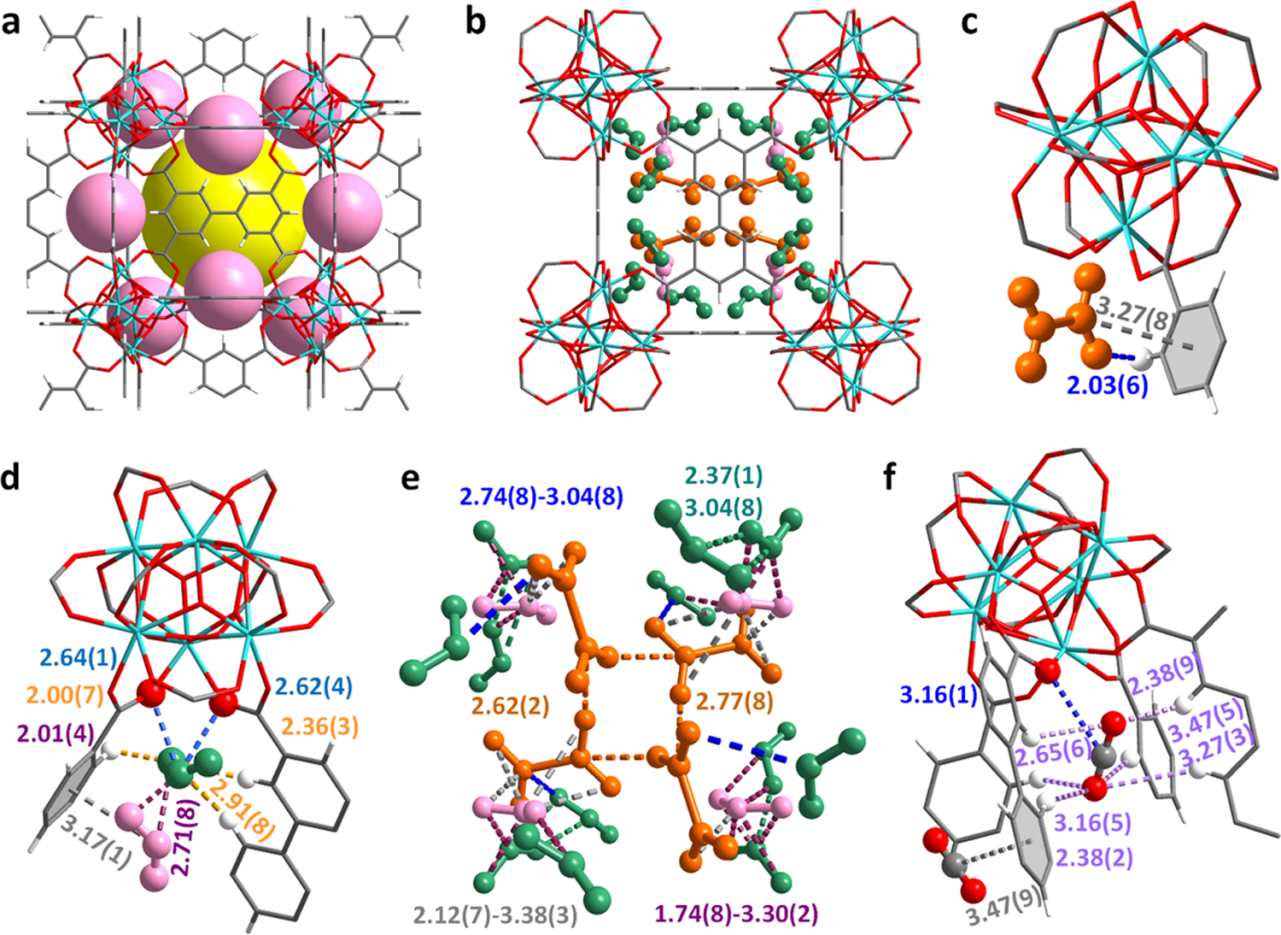
Views of the crystal structures of Zr-bptc, [Zr_6_O_4_(OH)_4_(bptc)_3_·(NO_2_)_7.5_·(NO_2_)_2.3_·(N_2_O_4_)_4.1_], and [Zr_6_O_4_(OH)_4_(bptc)_3_·(CO_2_)_2.8_]. All structures were derived from Rietveld refinements of *in situ* synchrotron X-ray powder diffraction data collected at 298 K (C: gray; N: blue; O: red Zr: cyan; H: white). (a) Metal–ligand cage A (yellow) and B (rose) in Zr-bptc; (b) packing of adsorbed NO_2_ and N_2_O_4_ molecules in Zr-bptc; (c) enlarged view of binding sites of N_2_O_4_ in Zr-bptc; (d) enlarged view of the binding site of monomer NO_2_ in Zr-bptc; (e) packing of NO_2_–N_2_O_4_ in the pore of Zr-bptc (orange: N_2_O_4_; sea green: NO_2_ at site I; rose: NO_2_ at site II; key dipole–dipole interactions are labeled); and (f) enlarged view of the binding site of CO_2_ in Zr-bptc.

Rietveld refinement of the high-resolution synchrotron X-ray powder diffraction data of NO_2_-loaded Zr-bptc, [Zr_6_O_4_(OH)_4_(bptc)_3_·(NO_2_)_7.5_·(NO_2_)_2.3_·(N_2_O_4_)_4.1_], at 298 K revealed three independent binding sites, I, II, and III, which are assigned as NO_2_, NO_2_, and N_2_O_4_ molecules, respectively (Figures 2b, S15, and S20 and Table S3). The total crystallographic occupancy of NO_2_ molecules (18.0 NO_2_ per {Zr_6_} cluster) is slightly lower than that obtained from the isotherm (22.9 NO_2_ per {Zr_6_} cluster), which is likely due to the presence of a small amount of highly disordered NO_2_ molecules in the pores. The NO_2_ molecules at site I (7.5 NO_2_ per {Zr_6_} cluster) exhibit strong interaction with the carboxylate group [O_2_N···OOC = 2.64(1) and 2.62(4) Å] (Figure 2d). Furthermore, O centers from NO_2_ molecules form multiple supramolecular interactions with the aromatic C–H groups on benzene rings. The interaction [NO_2_···H–C = 2.00(7)], <C–H–O = 101.8(4)° and twofold hydrogen bond [NO_2_···H–C = 2.36(3), 2.91(8) Å, <C–H–O = 144.4(7), 156.5(7)°] (Table S4) stabilize the packing of NO_2_ molecules at site I. The multiple hydrogen bonds and dipole–dipole interactions between NO_2_ at site I and the framework suggest strong binding, consistent with the exceptionally high adsorption at low pressure. Three different dipole interactions including [O_2_N···benzene = 3.17(1) Å] and [NO_2_···NO_2_ = 2.01(4) and 2.71(8) Å] stabilize the NO_2_ molecules at site II (2.3 NO_2_ per {Zr_6_} cluster). Interestingly, NO_2_ molecules at site III (8.2 NO_2_ per {Zr_6_} cluster) are dimerized to N_2_O_4_ and stabilized by electrostatic and dipole interactions [N_2_O_4_···H–C = 2.03(6) and O_2_N···benzene = 3.27(8) Å] (Figure 2c). The packing of NO_2_–N_2_O_4_ molecules is sustained by multiple intermolecular dipole–dipole interactions based upon monomer-to-monomer, monomer-to-dimer, and dimer-to-dimer with distances ranging from 1.74(8) to 3.38(3) Å, offering an efficient storage environment (Figure 2e), consistent with the high *Q*_st_ of Zr-bptc (90 kJ mol^–1^). Interestingly, the NO_2_ and N_2_O_4_ molecules immobilized in the pores of Zr-bptc on saturation show an unprecedented packing density of 1.83 g cm^–3^ at 298 K, higher than that observed in MFM-300(Al) (1.73 g cm^–3^ at 298 K), MFM-520 (1.42 g cm^–3^ at 298 K), liquid NO_2_ and N_2_O_4_ (1.45 and 1.44 g cm^–3^, respectively, at 294 K), and close to that of solid N_2_O_4_ (1.94 g cm^–3^ at 140 K).^31^ This result confirms a highly efficient packing of NO_2_/N_2_O_4_ molecules within the pores of Zr-bptc.

Two independent binding sites, I’ and II’, were located in cages B and A, respectively, in CO_2_-loaded Zr-bptc, [Zr_6_O_4_(OH)_4_(bptc)_3_·(CO_2_)_2.8_] (Figures S16–S19). The total crystallographic occupancy of 2.8 CO_2_ per {Zr_6_} cluster is lower than that obtained from the isotherm (5.2 CO_2_ per {Zr_6_} cluster), likely due to the presence of a large amount of highly disordered CO_2_ molecules in the pore owing to the weak host–guest interactions. CO_2_ molecules at site I’ (0.93 CO_2_ per {Zr_6_} cluster) display dipole interactions with the carboxylate group [O_2_C···OOC = 3.16(1) Å] (Figure 2f). In addition, multiple weak supramolecular interactions were observed between CO_2_^I’^ molecules and the benzene ring [O=C=O···H–C = 2.38(2)–3.47(5) Å] (Table S4). The CO_2_ molecules at site II’ (1.87 CO_2_ per {Zr_6_} cluster) are stabilized by the π···π interaction with the phenyl ring [O_2_C···benzene = 3.47(9) Å]. Overall, the host–guest binding interaction is notably weaker than that of NO_2_, consistent with low *Q*_st_ (28 kJ mol^–1^) and the selective uptake of NO_2_ in the breakthrough experiment.

**Figure 3:**
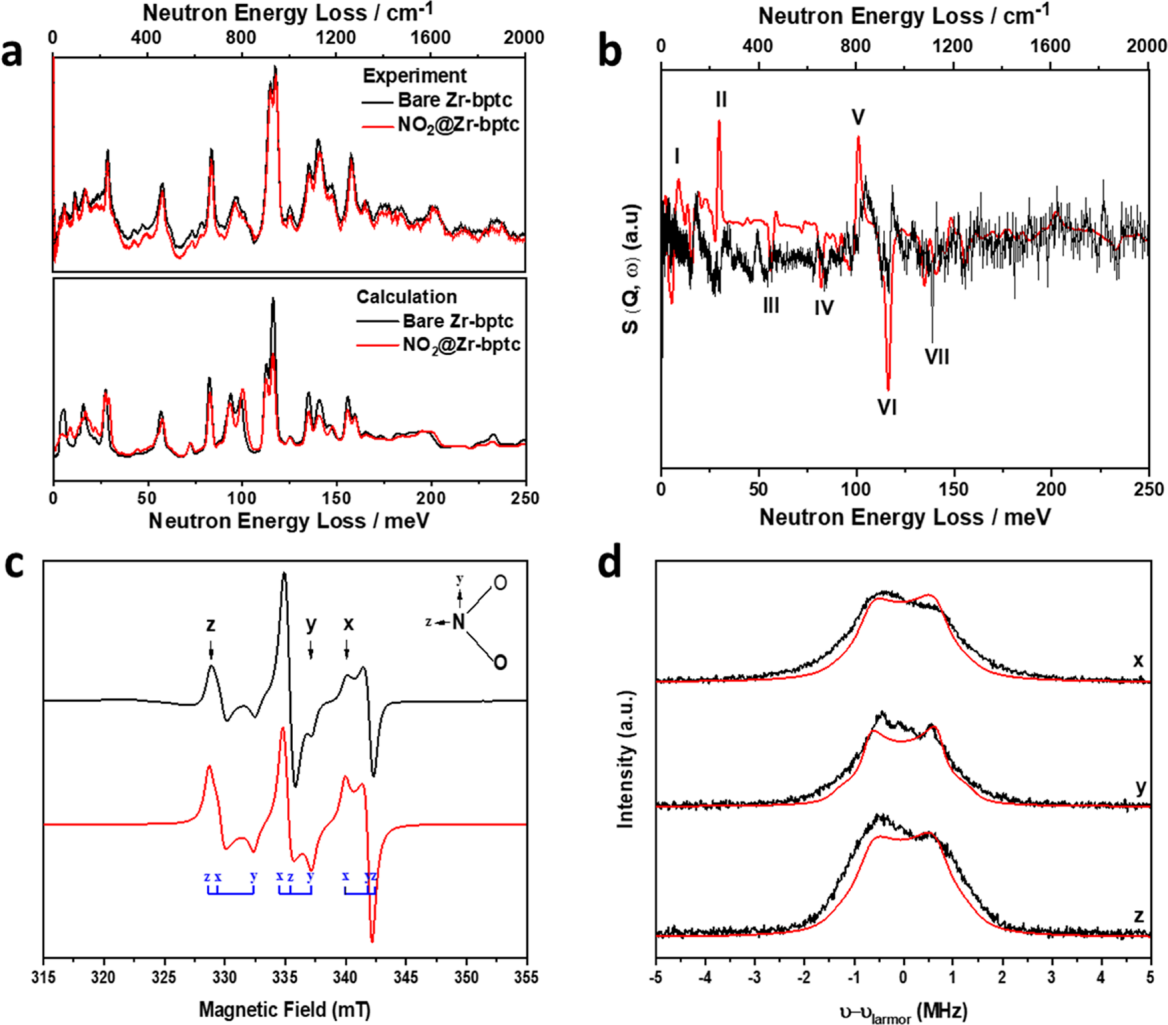
Spectroscopic data. (a) Comparison of the experimental (top) and DFT-simulated (bottom) INS spectra for bare and NO_2_-loaded Zr-bptc; (b) comparison of the difference plots for experimental and DFT-calculated INS spectra of bare and NO_2_-loaded Zr-bptc. No scale factor was used for the DFT calculations. *S*, dynamic structure factor; *Q*, difference between the incoming and outgoing wave vectors; ℏω, the energy change experienced by the sample; (c) continuous-wave X-band (9.72 GHz) EPR spectrum of NO_2_@Zr-bptc at 10 K (black) and simulation (red) with *g_x_* = 2.0055, *g_y_* =1.991, and *g_z_* =2.0028 and ^14^N nuclear hyperfine interactions (nuclear spin, *I* = 1) of *A_x_* = 145, *A_y_* = 127, and *A_z_* =185 MHz, where *x*, *y*, and *z* define the NO_2_ molecular axes (inset). NO_2_ has a *C*_2*v*_ point symmetry with the z axis along the C_2_ rotation axis, *y* parallel to the O–O vector, and x normal to the NO_2_ plane; (d) X-band Davies ENDOR spectrum (black) at 5 K and the static magnetic fields indicated, shown by the arrows in panel (c), dominantly selecting the NO_2_*x*, *y*, and *z* axes (top to bottom), respectively. ENDOR gives pairs of transitions separated by the effective hyperfine coupling for the orientation selected, centered on the Larmor frequency of the nucleus being probed (14.9 MHz for ^1^H at 350 mT). The black lines are the experimental spectra, and the red lines are the calculated spectra.

*In situ* INS, coupled with DFT calculations, enabled the visualization of the binding dynamics for NO_2_-loaded Zr-bptc with a focus on the −CH groups involved in the supramolecular contacts. Seven major changes in the INS spectra were observed on the adsorption of NO_2_ in Zr-bptc (Figure 3a,b). Peaks I–III at low energy transfer (<60 meV) and peaks IV–VII at high energy (80–150 meV) correspond to deformational modes of the phenyl ring and the twisting/wagging/scissoring modes of the aromatic −CH groups, respectively. These changes support the direct interactions between adsorbed NO_2_/N_2_O_4_ molecules and the soft −CH groups, consistent with the *in situ* crystallographic analysis.

The presence of adsorbed NO_2_ monomers in Zr-bptc is confirmed unambiguously by EPR spectroscopy on NO_2_@Zr-bptc at 10 K (Figure 3c), which shows signals for immobilized NO_2_ with a resolution of both the anisotropic electronic *g*-factor and ^14^N hyperfine interaction.^32^ The spectral line width is approximately double that of NO_2_-loaded MFM-300(Al)^25^ (Table S5), reflecting the higher concentration of monomeric NO_2_ in the pores of Zr-bptc, as found by the crystallographic study. The interaction of NO_2_ with the framework was probed by Davies ENDOR (electron–nuclear double resonance) spectroscopy at 5.7 K, which reveals weak ^1^H hyperfine interactions with frequencies around 2 MHz (Figure 3d).^33^ Based on a point-dipole model, these can be reproduced with a H···NO_2_ distance of 3.8 Å. The ENDOR spectra are rather broad and relatively insensitive to the magnetic field of measurement, *i.e*., with respect to the orientation of the NO_2_ molecule. This is presumably because of the multiple monomer NO_2_ binding sites within the pores of Zr-bptc; much stronger orientation selection is observed for NO_2_@MFM-300(Al) system, which has only one monomer binding site. This result is consistent with the enhanced packing density of NO_2_ in Zr-bptc. The H···NO_2_ distance from ENDOR at 10 K is longer than that found from the structural model determined at room temperature, indicating that the trapped NO_2_ molecules form stronger intermolecular interactions at low temperature. On warming the sample (200–360 K; Figure S21), the spectra broaden and the signal intensity increases as a function of the monomer–dimer equilibrium in the pores.^26^

**Figure 4:**
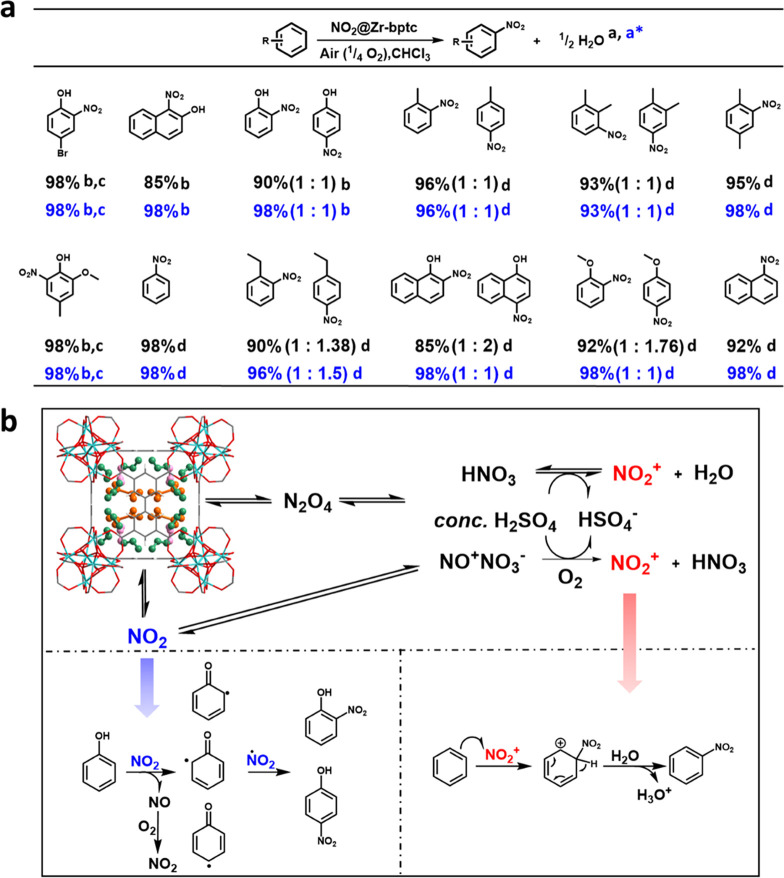
Schematic view of the utilization of NO_2_ pollutant for nitration processes. (a) Views of nitro compounds prepared using captured NO_2_. Yields are based on the captured NO_2_. ^a^NO_2_@Zr-bptc-N was formed from Zr-bptc loaded with NO_2_ at 2500 ppm at 298 K to give a total capacity of NO_2_ of 5.1 mmol g^–1^. Conditions for nitration: a sample of NO_2_@Zr-bptc-N containing 0.51 mmol of NO_2_ was treated with 0.75 mmol of aromatic substrate in 5 mL of CHCl_3_ at room temperature. Yields and selectivity are in black. ^a*^NO_2_@Zr-bptc-N* was formed from Zr-bptc loaded with NO_2_ at 1 bar at 298 to give a total capacity of NO_2_ of 14.3 mmol g^–1^. A sample of NO_2_@Zr-bptc-N* containing 1.43 mmol of NO_2_ was treated with 1.5 mmol of aromatic substrate in 5 mL of CH_2_Cl_2_ at room temperature. Yields and selectivity are in blue. ^b^Reaction conducted at 0 °C within 0.5 h; ^c^the reaction was completed within 1.0 h; ^d^a catalytic amount (1%) of *conc.* H_2_SO_4_ was added, and the reaction time was extended to 6.0 h; (b) proposed mechanism for the nitration reaction of phenol and benzene by NO_2_@Zr-bptc-N. The captured NO_2_ is released from the MOF into the reaction mixture as N_2_O_4_ or NO_2_. In the presence of *conc.* H_2_SO_4_, NO_2_^+^ is produced in the presence of O_2_, which drives the nitration process. For the nitration process in the absence of *conc*. H_2_SO_4_, the reaction occurs between NO_2_-derived radicals and substrate.^37,38^ The MOF plays a key role in the enrichment of NO_2_ from gas mixtures and facile release of captured NO_2_ into the reaction system.

Nitration is widely used in the chemical industry to produce important nitro compounds (*e.g*., nitrobenzene), which are key intermediates for the synthesis of a wide range of explosives, colorants, agrochemicals, and pharmaceuticals.^8,9,26,34^ The global market for nitrobenzene is $9.8 billion in 2020 and is projected to reach $14.5 billion by 2027.^35^ The construction industry is the dominant end user of nitrobenzene and consumes approximately half of the world’s annual production. In addition, nitrobenzene is also used in the synthesis of paracetamol, which serves as a generic medicine globally. For other examples, nifedipine, entacapone, and niclosamide are nitro-group-containing medicines and are widely used to treat hypertension, Parkinson’s disease, and tapeworm infections, respectively.^36^ Nitration in the chemical industry is usually carried out using a mixture of concentrated acids (*e.g*., 20% nitric acid, 60% sulfuric acid, and 20% water for mononitration) at 80–120 °C.^9^ Because these processes are highly exothermic, enormous amounts of energy are consumed by cooling. Additionally, the industrial production of nitric acid *via* the oxidation of ammonia in the Ostwald process carries huge carbon footprints.^10^ Thus, nitration is widely considered one of the most energy-consuming and hazardous industrial processes. Although NO_2_ gas can be used as a nitration reagent, its highly corrosive and toxic nature renders the operation highly challenging.^39^ In addition, byproducts from overnitration and overoxidation are often obtained when using gaseous or liquid NO_2_ in nitration processes,^40,41^ and this is also confirmed by our tests on the nitration of a range of substrates with NO_2_ gas (Figures S27–S36). We sought to convert the captured NO_2_ by Zr-bptc to valuable nitro compounds at room temperature and without the use of mixtures of concentrated acids. Activated Zr-bptc was loaded with NO_2_ at 2500 ppm, which gives an uptake of 5.1 mmol g^–1^ (denoted as NO_2_@Zr-bptc-N; see the Experimental Methods and SI sections for details). Activated Zr-bptc was also loaded with NO_2_ at 1 bar, which gives an uptake of 14.3 mmol g^–1^ (denoted as NO_2_@Zr-bptc-N*; see the Experimental Methods and SI sections for details). A series of nitration experiments of aromatic compounds were then undertaken using NO_2_@Zr-bptc-N as the nitration source (Figures 4a and S37–S69). A fixed amount of NO_2_@Zr-bptc-N (containing 0.51 mmol NO_2_) was added to the mixture of aromatic substrates (0.75 mmol) and CHCl_3_ (5.0 mL) under stirring at room temperature or 0 °C, and the product was isolated by centrifugation and the supernatant was collected and analyzed by NMR spectroscopy. Importantly, the captured NO_2_ molecules can be quantitatively converted to nitro compounds in the absence or the presence of a catalytic amount (1%) of sulfuric acid, which is also confirmed by EPR analysis of the postreaction mixture that confirms the absence of residual NO_2_ (Fig. S24). Mononitration was achieved in a series of key aromatic compounds, including benzene, naphthalene, and *p*-xylene with yields of >85%. *O*- and *p*-substituted nitro compounds were obtained in nearly 1:1 ratio for phenol, toluene, and *o*-xylene and >1:1.4 ratio for ethylbenzene, anisole, and naphthalen-2-ol. Trace amounts of *m*-substituted nitro compound, which is a common product in the nitration process with gaseous NO_2_,^40,41^ were observed in these reactions. Control experiments using H_4_bptc or ZrOCl_2_·8H_2_O as the capture material were conducted, and little conversion was observed (Figure S26), demonstrating the key role of Zr-bptc in enriching NO_2_ from gas mixtures and releasing NO_2_ into the reaction medium (Figure S25).

**Figure 5:**
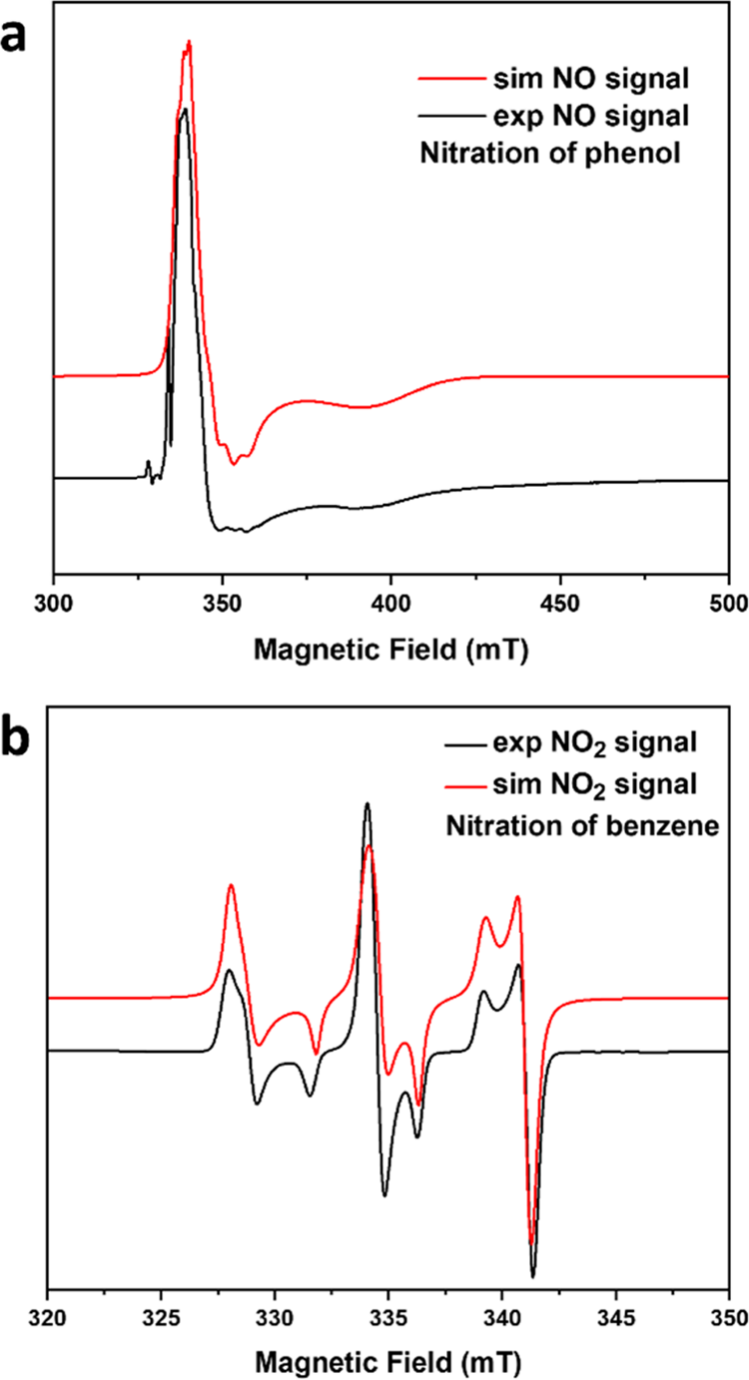
EPR spectroscopy. (a) Continuous-wave X-band (9.72 GHz) EPR spectra of the nitration reaction mixture of phenol at 10 K (black) and simulation (red); and (b) continuous-wave X-band (9.72 GHz) EPR spectra of the nitration reaction mixture of benzene at 10 K (black) and simulation (red), confirming the absence of NO.

**Figure 6:**
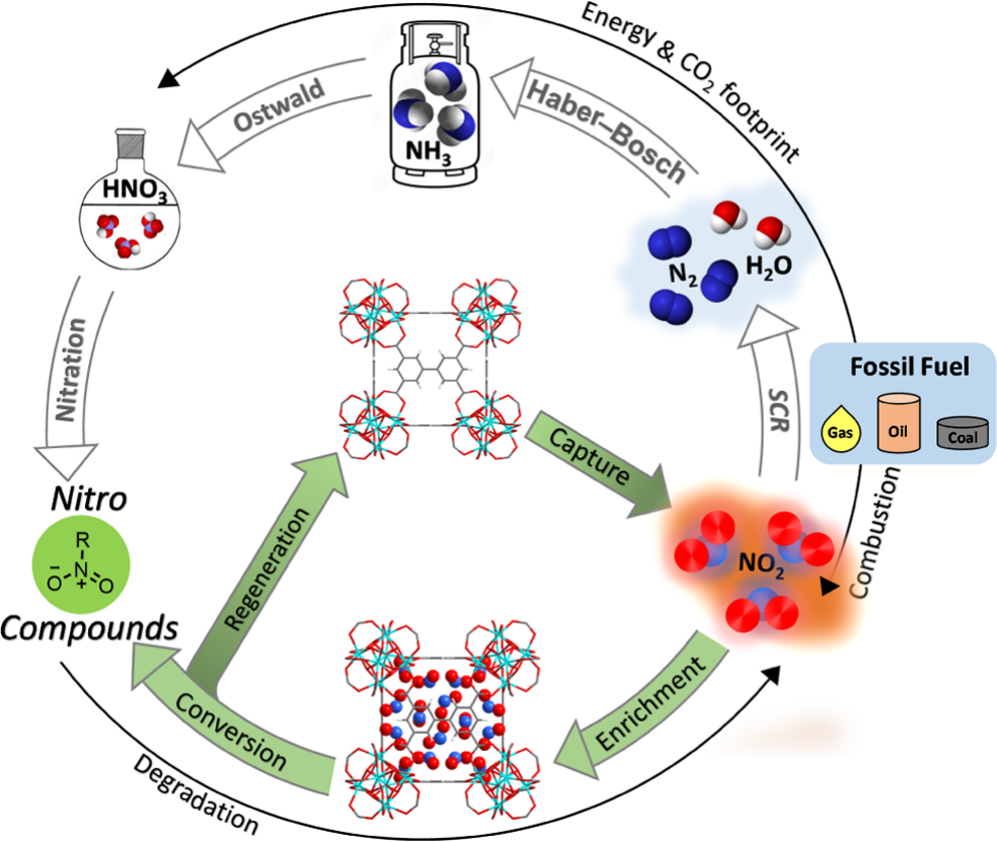
Illustration of the nitrogen cycle for the synthesis of nitro compounds and regeneration of sorbent materials.

In addition, EPR spectroscopy was employed to validate the proposed mechanism (Figure 4b), and the presence of signals of NO in the nitration of phenol demonstrated the presence of an alternative reaction pathway to nitronium ion-induced nitration, consistent with the short reaction time required (Figure 5a,b). The Zr-bptc recovered from these reactions can be regenerated fully *via* heating under dynamic vacuum. Uptake of NO_2_ and conversion efficiency in the synthesis of nitrobenzene were stable over three consecutive cycles (Figures S2 and S70 and Table S6). Thus, the integration of waste NO_2_ into the production of important nitro compounds not only holds the promise to reduce the carbon footprint of existing industrial nitration processes but also fulfills the requirement of “waste-to-chemicals” processes (Figure 6).

## Conclusions

The robust porous MOF material, Zr-bptc, exhibits exceptional adsorption capacity of NO_2_ under both low- and high-pressure conditions and high heats of adsorption with an unprecedented packing density of NO_2_ in the pore. *In situ* synchrotron X-ray diffraction and INS studies, coupled with DFT calculations, unravel the molecular details on the host–guest binding that result in excellent adsorption performance. *In situ* EPR analysis demonstrates the existence of NO_2_–N_2_O_4_ equilibrium in this system. The successful synthesis of various fine chemicals from the key air pollutant demonstrates a promising “waste-to-chemicals” process for the recovery and circular utilization of reactive nitrogen-based wastes.

## Supporting Information Available

Additional crystallographic information, gas adsorption data, thermogravimetric analysis, density functional theory (DFT) calculations, breakthrough data, stability test data, and conversion of captured NO_2_ are available (PDF)
