# Supplementary material for: Synthesis of Nitro Compounds from Nitrogen Dioxide Captured in a Metal-Organic Framework
Source: J Am Chem Soc. 2022 Oct 5;144(41):18967–75. doi: 10.1021/jacs.2c07283 (PMC9585588; doi:10.1021/jacs.2c07283)
Supplement: Supplementary file 1 — ja2c07283_si_001.pdf [file ja2c07283_si_001.pdf]

Supplementary Information

Synthesis of nitro compounds from nitrogen dioxide captured in a metal-organic framework

Jiangnan Li[1], Zi Wang[1], Yinlin Chen[1], Yongqiang Cheng[2], Luke L. Daemen[2], Floriana Tuna[1,3], Eric J. L. McInnes[1], Sarah J. Day[4], Anibal J. Ramirez-Cuesta[2], Martin Schröder[1]* and Sihai Yang[1]*

[1] Department of Chemistry, University of Manchester, Manchester, M13 9PL (UK)

[2] Neutron Scattering Division, Neutron Sciences Directorate, Oak Ridge National Laboratory, Oak Ridge, TN 37831 (USA)

[3] Photon Science Institute, University of Manchester, Manchester, M13 9PL (UK)

[4] Diamond Light Source, Harwell Science Campus, Oxfordshire, OX11 0DE (UK)

1. Experimental Section……………………………………………………………………..…..…..3

1.1 Synthesis and Activation of Zr-bptc…………………………………………………………3

1.2 NO_2 Safety……………………………………………………………………………………..3

1.3 Gas Adsorption Isotherms ……………………………………………………………..…….3

1.4 Gas Separation by Breakthrough Experiments………………………….…...…..……...3

1.5 Thermogravimetric Analysis……………………………………………………………...3

1.6 *In situ* Synchrotron X-Ray Powder Diffraction……………………………..…..……..3

1.7 Inelastic Neutron Scattering (INS)……………………………………………………….4

1.8 DFT Modelling and Simulation…………………………………………..……..……4

1.9 Electron Paramagnetic Resonance (EPR) Experiment……………………...………….4

1.10 IAST Analysis of the Selectivity Data for Zr-bptc……………………………..…………....5

1.11 Calculation of Dynamic Selectivity………………………………………………………....5

1.12 General Procedure of Conversion………………………………………...…………….5

1.13 Reusability Tests for Zr-bptc……………………………………………...…………….5

2. Additional Data on Stability of Zr-bptc………………………………………………………….6

3. Additional Gas Adsorption Isotherms and Separations for Zr-bptc…………………….......8

4. Thermogravimetric Analytical Data………………………………..........…….....………......15

5. Calculation of Isosteric Heats of Adsorption……………………………………….…………18

6. Selectivity Data for Zr-bptc……………………………………………….......................…...22

7. Additional Structural Views and Data on Gas-loaded Zr-bptc………………………………..23

8. Additional Analysis of EPR Data ………………………………………………………………31

9. Conversion of Captured NO_2 in Zr-bptc…………………………………………………..35

10. References………………………………………………………………...……………...85

1. Experimental Section

1.1 Synthesis and Activation of Zr-bptc

All reagents were used as received from commercial suppliers without purification. Synthesis of $Zr_6O_4(OH)_4(C_{16}H_6O_8)_3$ (Zr-bptc):[1] Zirconium (IV) oxychloride octahydrate ($ZrOCl_2\cdot8H_2O$, 322 mg, 1.0 mmol) was added to H_4bptc (H_4bptc = biphenyl-3,3',5,5'-tetracarboxylic acid) (330 mg, 1.0 mmol), N,N-dimethylformamide (DMF, 50 mL) and formic acid (50 mL) in a 200 mL Teflon reactor. This was sealed and transferred to a preheated oven at 120 °C. The reaction was heated at 120 °C for 3 days, cooled and a microcrystalline white powder obtained through centrifugation. The product was washed with DMF and methanol and activated at 573 K under dynamic vacuum prior to adsorption studies.

1.2 NO_2 safety

The hardware and piping involved in the supply, delivery and measurement of NO_2 were rigorously leak tested and used only within range of a NO_2 detection system with a sensitivity of 0.1 ppm.

1.3 Gas Adsorption Isotherms

Sorption isotherms for N_2, CH_4, CO_2 were recorded at different temperatures, maintained using a temperature-programmed water bath, on a Hiden Isochema IGA-003 system under ultra-high vacuum (10^{-10} bar) using a turbo pumping system. Ultra-pure research grade (99.999%) N_2, CH_4 and CO_2 were purchased from BOC or Air Liquide. In a typical gas adsorption experiment, 50 mg of acetone-exchanged Zr-bptc was loaded into the IGA system and activated at 573 K under dynamic high vacuum (10^{-10} bar) for 24 h to give fully desolvated Zr-bptc. Isotherms were performed on an IGA using ultrahigh purity (99.999%) N_2 at 77 K for void volumetric determination. The BET surface areas were calculated using the software integrated into the instrument.

1.4 Gas Separation by Breakthrough Experiments

Breakthrough experiments were performed on a Hiden Isochema IGA-003 with ABR attachments and a Hiden Analytical mass spectrometer to detect gases as they break through the sample bed. Experiments were carried out in a 7 mm diameter fixed-bed of 120 mm length packed with ~0.55 g of Zr-bptc powder (particle size < 1 micron). The sample was pre-activated at 573 K under vacuum and the sample loaded into the column and re-activated under a flow of He for 12 h. The fixed-bed was cooled to room temperature (298 K) using a temperature programmed water bath and the breakthrough experiment performed with a series of gas mixtures at atmospheric pressure and room temperature.

1.5 Thermogravimetric Analysis

The synthesis of MFM-520 and MFM-300(Al) was conducted using our previously reported methods[2-3]. TGA-DSC analysis of NO_2 adsorption in Zr-bptc, MFM-520 and MFM-300(Al) were analysed by activating the sample at 573, 393 and 423 K, respectively under a flow of dry N_2. The samples were then cooled to 303 K and a flow of NO_2 (diluted in He) was passed over the sample. Adsorption of NO_2 caused an increase in the weight of the sample as shown in Fig. S9-11. The weight gain of sample (m) and the heat flow were measured directly, and the integration of heat flow for NO_2 was found to be 3.58 J (the area of integration is indicated as H) in Zr-bptc. The heat of adsorption (Q_{st}) was calculated from the following equation:

$$Q_{st} = \frac{H \times M_w}{m}$$

where M_w is the molecular mass of the gas. The units of m, M_w and H are g, g/mol and kJ, respectively.

1.6 *In situ* Synchrotron X-Ray Powder Diffraction

High-resolution X-ray powder diffraction of bare, CO_2-loaded and NO_2-loaded Zr-bptc was carried out on beamline I11 of the Diamond Light Source. A high brightness monochromatic beam was produced by a Si(111) monochromator and double-bounce harmonic rejection mirrors. The beam was delivered to the main instrument hutch where five multi-analysing crystal-detectors (MAC) travel in an arc of 2θ around the sample.

Measurements were carried out in capillary mode and the sample environment controlled using an Oxford Cryosystems open-flow N_2 gas cryostat. The samples were ground to provide a uniform particle size, packed into a borosilicate capillary and mounted into a gas cell for *in situ* gas dosing. The sample was activated under vacuum (1 x 10^{-6} mbar) at 573 K for > 3 h to remove residual solvent molecules from the material. Diffraction data for the activated sample was collected and analysed to confirm that no residual solvent molecules are present in the pores. A gas dosing panel was used for *in situ* dosing of activated Zr-bptc at 1 bar CO_2. NO_2 loaded Zr-bptc was prepared directly in the laboratory. Details are tabulated in Tables S3.

1.7 Inelastic Neutron Scattering (INS)

INS spectra were recorded on the VISION spectrometer at Spallation Neutron Source, Oak Ridge National Laboratory (USA). VISION is an indirect geometry crystal analyser instrument that provides a wide dynamic range with high resolution. The sample of pre-activated Zr-bptc (573 K under vacuum) was loaded into a cylindrical vanadium sample container with an indium vacuum seal and connected to a gas handling system. The sample was degassed at 10^{-7} mbar at 393 K for 1 day to remove any remaining trace guest water molecules. The temperature during data collection was controlled using a closed cycle refrigerator (CCR) cryostat (10 ± 0.1 K). The loading of NO_2 was performed volumetrically at room temperature in order to ensure that NO_2 was present in the gas phase when not adsorbed and also to ensure sufficient mobility of NO_2 inside the crystalline structure of Zr-bptc. Subsequently, the temperature was reduced to below 10 K in order to perform the scattering measurements with minimum thermal motion for the framework host and adsorbed NO_2 molecules. Background spectra [sample can plus bare Zr-bptc] were subtracted to obtain the difference spectra.

1.8 DFT Modelling and Simulation

Vibrational frequencies and polarization vectors were calculated using CP2K (http://www.cp2k.org)[4], based on the mixed Gaussian and plane-wave scheme[5] and the Quickstep module[6]. The calculation used molecularly optimized Double-Zeta-Valence plus Polarization (DZVP) basis set[7], Goedecker-Teter-Hutter pseudopotentials[8], and the Perdew-Burke-Ernzerhof (PBE) exchange correlation functional[9]. The plane-wave energy cutoff was 400 Ry. The DFT-D3 level correction for dispersion interactions, as implemented by Grimme *et al*[10], was applied, with a cutoff distance of 15 Å. The calculation was performed on Gamma point only, with no symmetry constraint. Structural optimization was performed using the Broyden-Fletcher-Goldfarb-Shannon (BFGS) optimizer, until the maximum force is below 0.00045 Ry/Bohr (0.011 eV/Å). Finite displacement method was used for the phonon calculation, with incremental displacement of 0.01 Bohr (0.0053 Å). The INS spectrum was then simulated using the OClimax software[11].

1.9 Electron Paramagnetic Resonance Experiments

Pre-activated Zr-bptc (30 mg) was placed into a J. Young EPR quartz tube (4 mm o.d.). The sample then was evacuated under vacuum for 2 h at room temperature, and heated at 393 K overnight to activate the sample. The EPR tube was connected to a NO_2 cylinder equipped with a pressure regulator. The whole system was checked for leakage and the MOF sample was left for 1 h under 1 bar pressure of NO_2. After gas adsorption was complete the tube was evacuated at 10^{-2} mbar for 1 min, sealed and disconnected from the system at low temperature. The NO_2 gas was condensed into the EPR tube using a cold trap method where the sample volumes containing the MOF material were cooled to 77 K. The application of the cold trap method during the sealing of the quartz glass tubes ensured that the entire amount of loaded NO_2 was fully trapped within the EPR tubes. Loading of NO_2 were rigorously leak tested and used only within range of a NO_2 detection system with a sensitivity of 0.1 ppm.

CW EPR measurements were carried out at X-band (*ca*. 9.5 GHz) on a Bruker EMX spectrometer equipped with temperature control system. A modulation amplitude of 1 mT was used with microwave power of ~ 2.0 mW after testing for saturation of spectral lines. EPR spectra were simulated using the EasySpin toolbox for Matlab[12]. Pulsed electron paramagnetic resonance (EPR) measurements of NO_2-loaded Zr-bptc sample was detected at X-band (ca. 9.7 GHz) on a Bruker Elexsys E580 spectrometer. Echo-detected field swept (EDFS) spectra were measured at X-band using the pulse sequence ($\pi/2 - \tau - \pi - \tau -$ echo) with $\pi/2$ and π pulse lengths of 200 ns and 400 ns respectively. The interpulse delay τ was 200 ns. Electron-nuclear double resonance

(ENDOR) measurements used the Davies sequence (π_{inv} − RF −π/2 − τ − π − τ – echo) with microwave inversion and radiofrequency (RF) π-pulse durations of 200 and 1200 ns, respectively.

Variable-temperature EPR intensity measurements

For the variable-temperature EPR intensity data, spectra were measured through an entire temperature loop, heating from 200 to 360 K and then cooling from 360 to 200 K, equilibrating for 10 min at each temperature.

1.10 IAST Analysis of the Selectivity Data for Zr-BPTC

Ideal adsorbed solution theory (IAST) was used to determine the selectivity factor, *S*, for binary mixtures using pure component isotherm data. The selectivity factor, *S*, is defined according to the following Equation where x_1 is the amount of component 1 adsorbed and y_1 is the mole fraction of component 1 in the gas phase at equilibrium.

$$S = \frac{x1/y1}{x2/y2}$$

The IAST adsorption selectivity was calculated for NO_2/CO_2 (1: 99) and NO_2/N_2 (1: 99) compositions at 298 K and a total pressure of 1 bar.

1.11 Calculation of Dynamic Selectivity

Dynamic adsorption selectivity (*S*) of an adsorbent can be obtained from experimental breakthrough curves by using the following equation:

$$S = \frac{qi/qj}{Xi/Xj}$$

where *q* and *X* are the adsorbed amounts and molar fractions in the bulk phase of the components i and j, respectively. The calculated selectivity for NO_2/N_2 (2500 ppm diluted in N_2 and He) and NO_2/CO_2 (2500 ppm/6.25%; *v*/*v*) is 151 and 24, respectively.

1.12 General Procedure for Conversion

Activated Zr-bptc (100 mg) was dosed with NO_2 at 1 bar and 298 K for 1 h to reach adsorption equilibrium (denoted as NO_2@Zr-bptc-N*). The quantity of captured NO_2 in NO_2@Zr-bptc-N* was determined by TGA, which shows 40% weight loss between 25 and 330 °C, corresponding to an uptake of 14.3 mmol g^{-1} (Fig. S23), consistent with that (13.8 mmol g^{-1}) observed in the adsorption experiment. Aromatic substrates (1.5 mmol) and CH_2Cl_2 (5.0 mL) were added to a 10 mL round-bottom flask under stirring for 5 mins to obtain a clear solution. NO_2@Zr-bptc-N* (containing 1.43 mmol NO_2) was added to the mixture under stirring at room temperature or 0 °C. Upon completion of reaction, the mixture was centrifuged, the solid recycled and the supernatant collected and reduced under vacuum for analysis.

NMR spectroscopy and preparative thin layer chromatography (TLC) were used to purify and quantify the conversion and the yield of nitro compounds. The recyclability of Zr-bptc was studied for the conversion of benzene over three cycles. No apparent leaching (<0.1 ppm, monitored by a sensor) of NO_2 was observed from the reaction system, consistent with the high conversion of NO_2.

1.13 Reusability Tests for Zr-bptc

Powder X-ray diffraction (PXRD) patterns were obtained using PANalytical X'Pert Pro MPD diffractometer in Bragg-Brentano geometry using Cu-Kα_1 radiation (λ = 1.5406 Å). Zr-bptc (30 mg) was treated with NO_2 adsorption/conversion experiments and after the measurements, PXRD patterns were measured (Fig. S1 and S70).

2. Additional Data on Stability of Zr-bptc

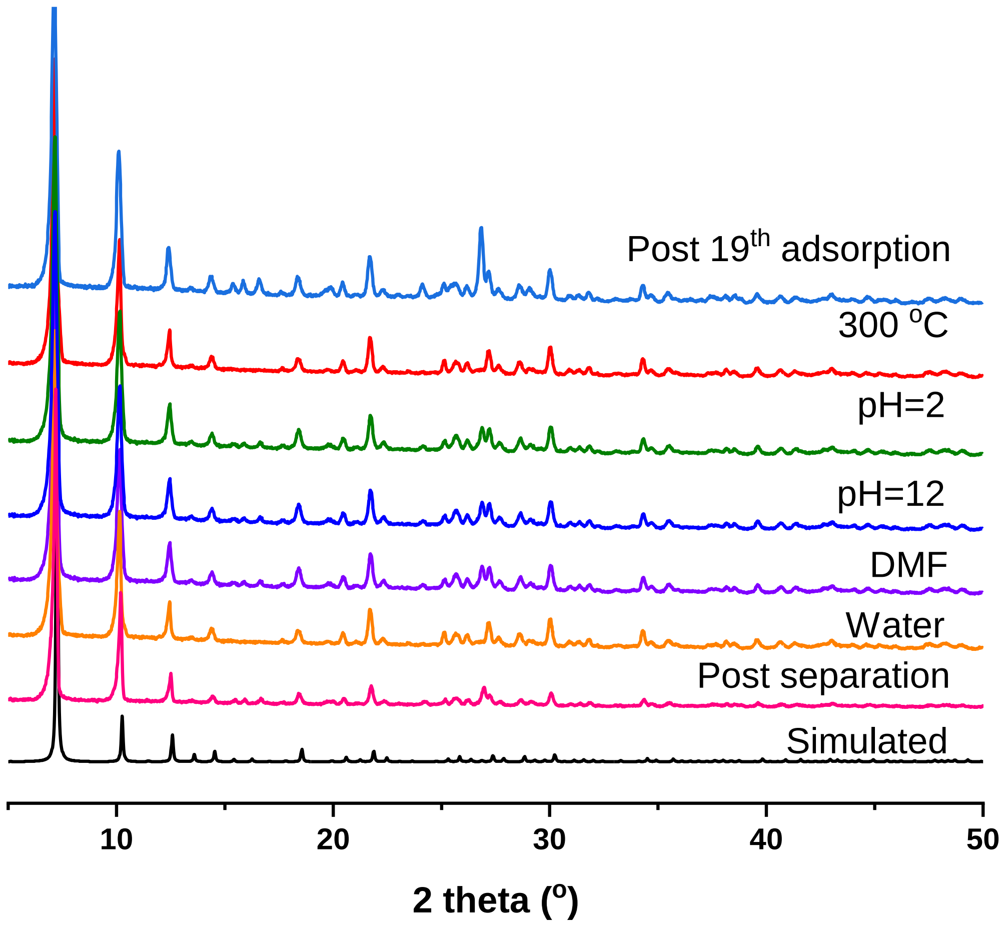

Figure S1. PXRD patterns for Zr-bptc. Simulated Zr-bptc (black); Zr-bptc after separation experiments (pink); Zr-bptc soaked in water for 1 week (orange); Zr-bptc soaked in DMF for 1 week (violet); Zr-bptc soaked in NaOH with pH=12 for 12 h (blue); Zr-bptc soaked in HCl with pH=2 for 12 h (dark green); Zr-bptc heated at 300 °C under N_2 (red) for 12 h and Zr-bptc post 19 cycles of NO_2 adsorption (light blue).

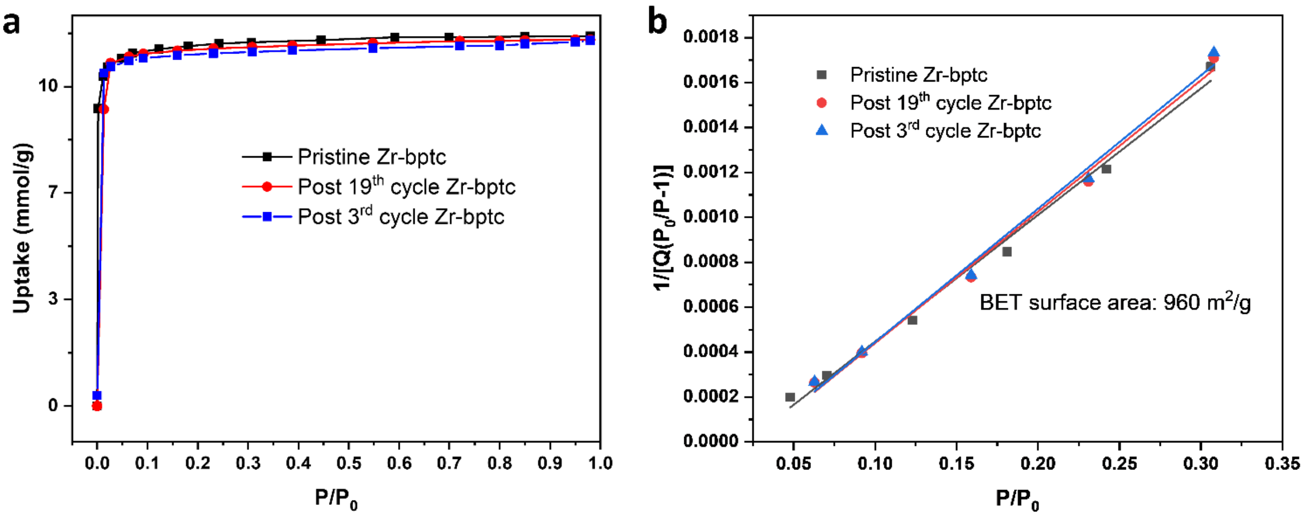

Figure S2. (a) N_2 isotherms at 77 K and (b) plots of BET surface area of pristine Zr-bptc, and the sample after 19 cycles of NO_2 adsorption and desorption, and after three cycles of aromatic substrate conversion using trapped NO_2.

3. Additional Gas Adsorption Isotherms and Separations for Zr-bptc

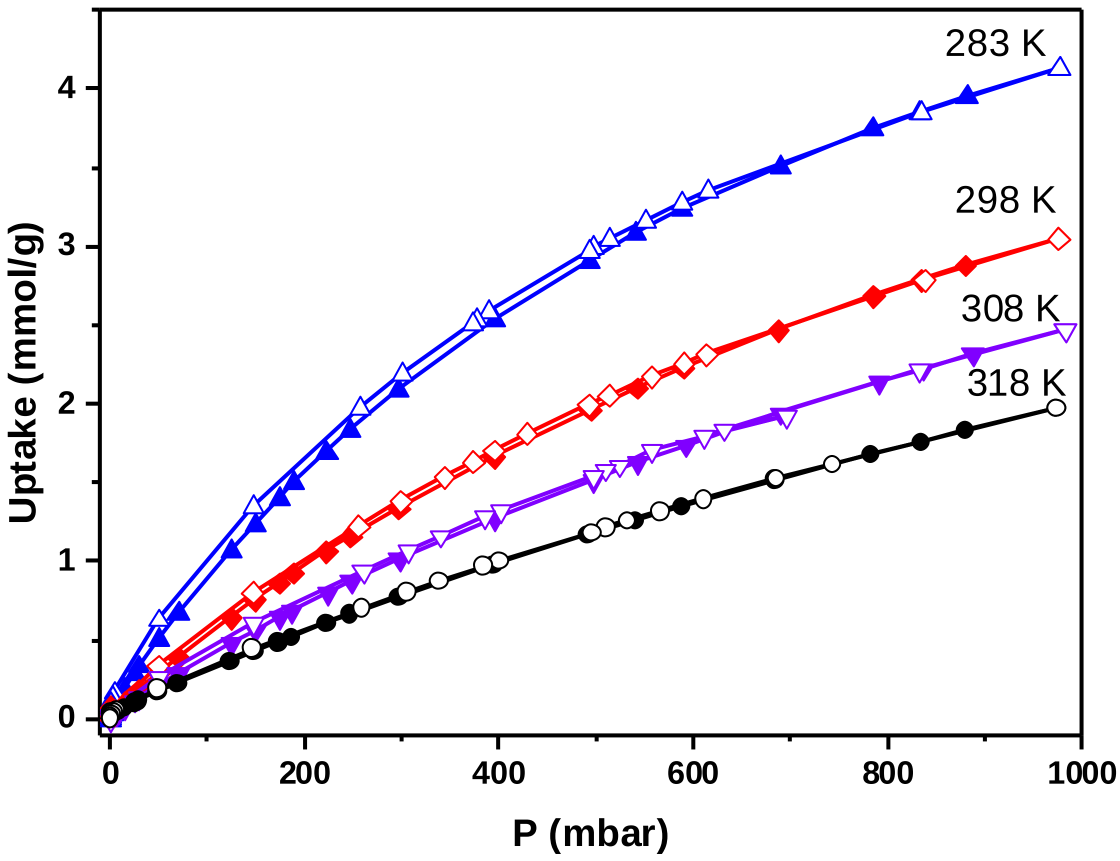

Figure S3. Sorption isotherms for CO_2 in Zr-bptc 283 K (blue), 298 K (red), 308 K (violet) and 318 K (black); solid adsorption, open desorption.

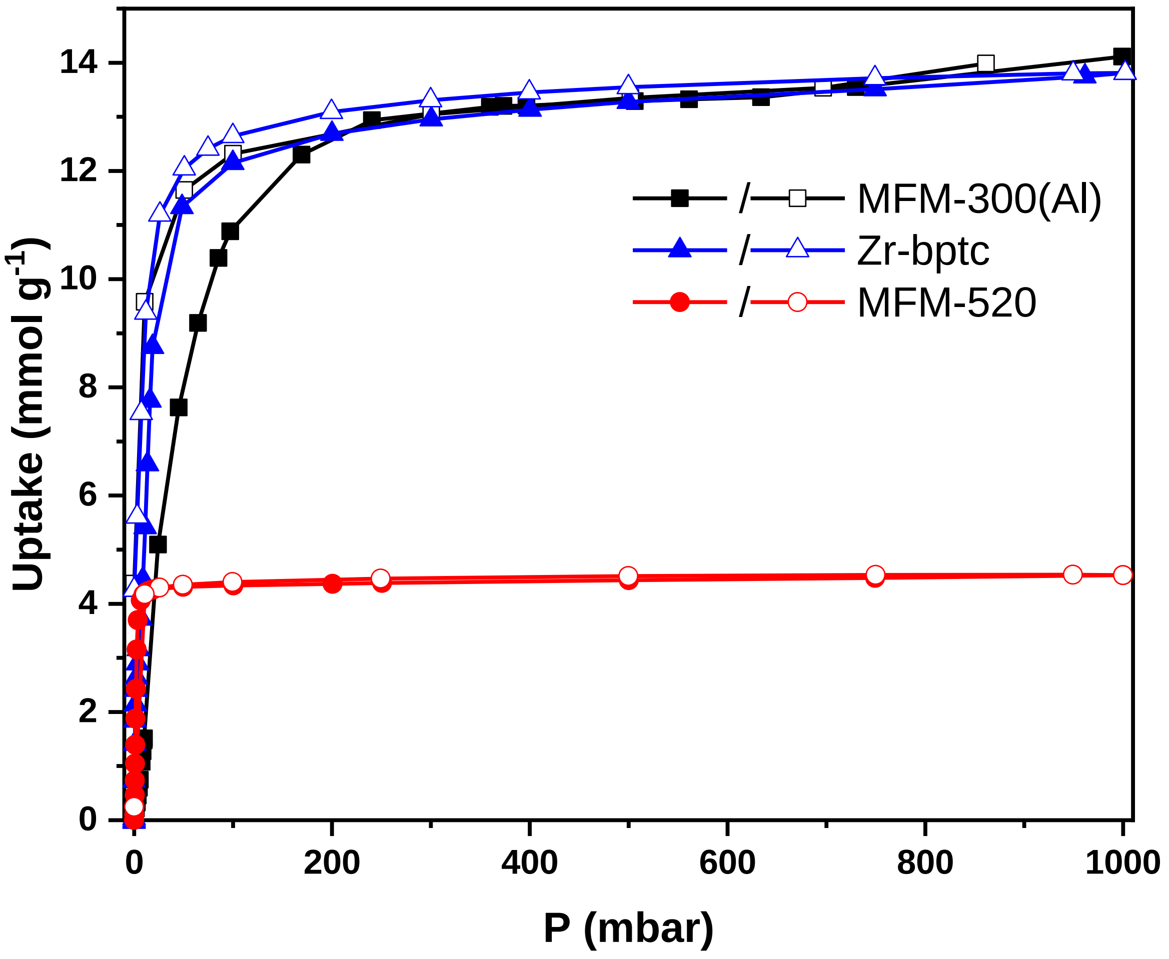

Figure S4. Comparison of NO_2 sorption isotherms in Zr-bptc (blue), MFM-300 (Al) (black) and MFM-520 (red) at 298 K; solid adsorption, open desorption.

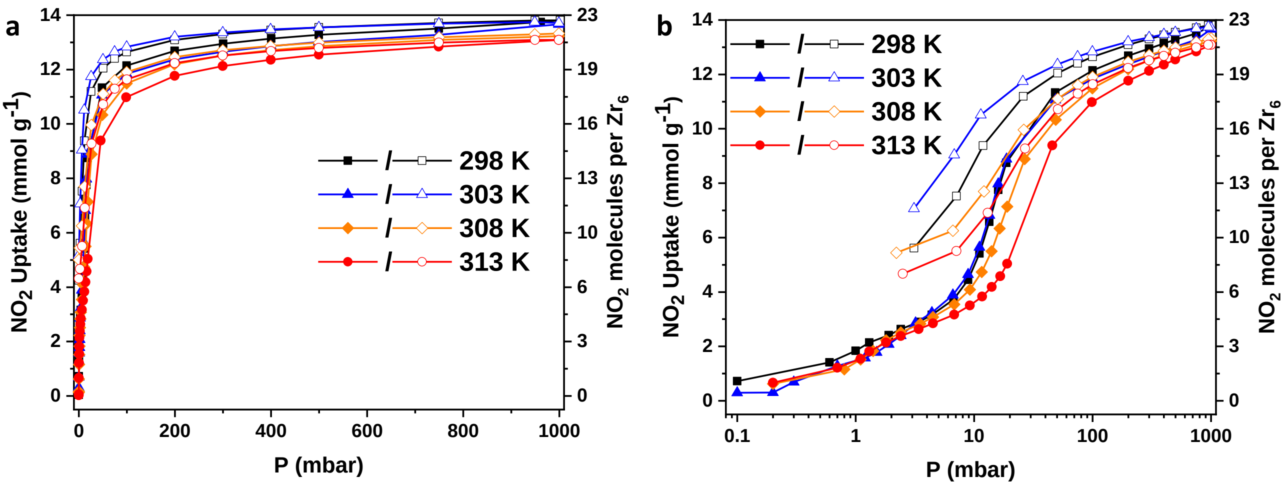

Figure S5. Desorption isotherms for (a) NO_2 in Zr-bptc, and (b) NO_2 in Zr-bptc on a logarithmic scale (solid: adsorption, open: desorption) at various temperatures.

Table S1. Summary of NO_2 adsorption capacity in reported MOF materials

Materials	BET surface area (m^2 g^{-1})	Experiment capacity (mmol g^{-1})	Measurement method	Conditions	Stability	Ref
Zr-bptc	960	13.8 (dry)	Isotherm	Pure NO_2 at 298 K and 1 bar	Y	This work
Zr-bptc	960	4.9 (dry)<br>4.0 (wet)	Breakthrough	2,500 ppm NO_2/He at 298 K	Y	
MFM-520	313	4.53 (dry)	Isotherm	Pure NO_2 at 298 K and 1 bar	Y	3
MFM-300 (Al)	1,370	14.0 (dry)	Isotherm	Pure NO_2 at 298 K and 1 bar	Y	2
MFM-300 (V^{IV})	-	13.0 (dry)	Isotherm	Pure NO_2 at 298 K and 1 bar	Y	13
HKUST-1	909	2.30 (dry)<br>1.17 (wet)	Breakthrough	1,000 ppm NO_2/air at 298 K	N	14
HKUST-1/GO	989-1,002	2.43-2.91(dry)<br>0.83-1.28(wet)			N	
UiO-66	891	1.59 (dry)<br>0.87 (wet)	Breakthrough	1,000 ppm NO_2/air at 298 K	N	15
UiO-67	1,372	1.72 (dry)<br>2.56 (wet)			N	
U-ZrBDC	1,070	0.80 (dry)<br>2.20 (wet)	Breakthrough	1,000 ppm NO_2/air at 298 K	N	16
M-ZrBDC	6	0.06 (dry)<br>0.22 (wet)			N	
U-ZrBDPC	2,040	1.61 (dry)<br>3.35 (wet)			N	
M-ZrBDPC	75	0.89 (dry)<br>2.02 (wet)			N	
UiO-66	990	3.8 (dry)	Micro-breakthrough	2,138 ppm NO_2 at 293 K	N	17
UiO-66-vac	1,590	3.9 (dry)			N	
UiO-66-ox	1,410	8.4 (dry)			N	
UiO-66	-	8.8 (dry)<br>13.2 (wet)	Micro-breakthrough	500-700 ppm NO_2/air at 293 K	N	18
UiO-66-NH_2	987	20.3 (dry)<br>31.2 (wet)			N	
HKUST-1	-	6.5 (dry)<br>26.1 (wet)			N	
Ce-UiO-66	1,035	2.07 (dry)<br>1.15(wet)	Breakthrough	1,000 ppm NO_2/air at 298 K	N	19
Ce-UiO-67	2,302	1.87 (dry)<br>1.85 (wet)			N	
CuBTC-U	1,201	0.72(dry)<br>1.52(wet)	Breakthrough	1,000 ppm NO_2/N2 at 298 K	N	20
CuBTC-M	43	0.50(dry)<br>0.065(wet)			N	
MOF-808	1,822	1.19(dry)<br>0.82(wet)	Micro-breakthrough	250 ppm NO_2/air at 298 K	N/A	21
G808	1,110	1.52(dry)<br>4.15(wet)			N/A	
MOF-808-NH_2	1,174	3.36(dry)<br>3.21(wet)			Y	
G808	1,037	3.67(dry)<br>6.36(wet)			Y	
UiO-66-NH_2	1,165	5.95(dry)<br>7.69(wet)			N/A	
G66-NH_2	762	8.65(dry)<br>9.93(wet)			N/A	

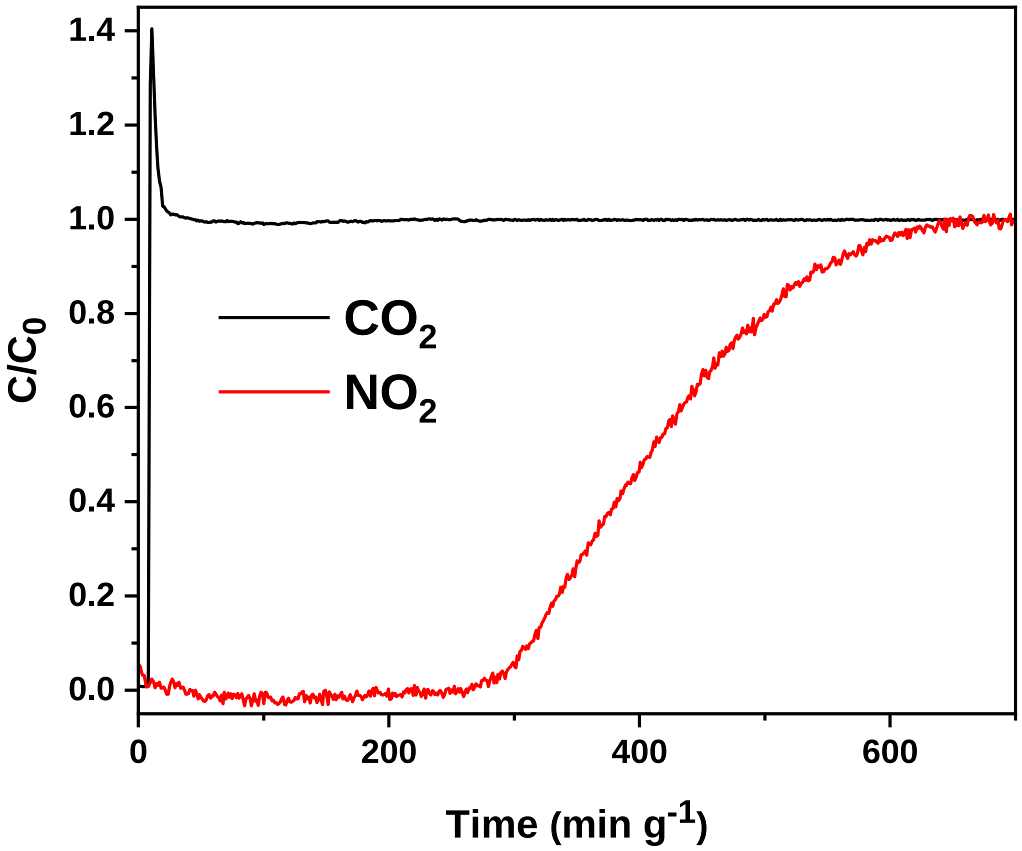

Figure S6. Breakthrough plots for CO_2/NO_2 gas mixtures (2500 ppm NO_2 and 15% CO_2 diluted in He, total flow rate: 40 mL min^{-1}).

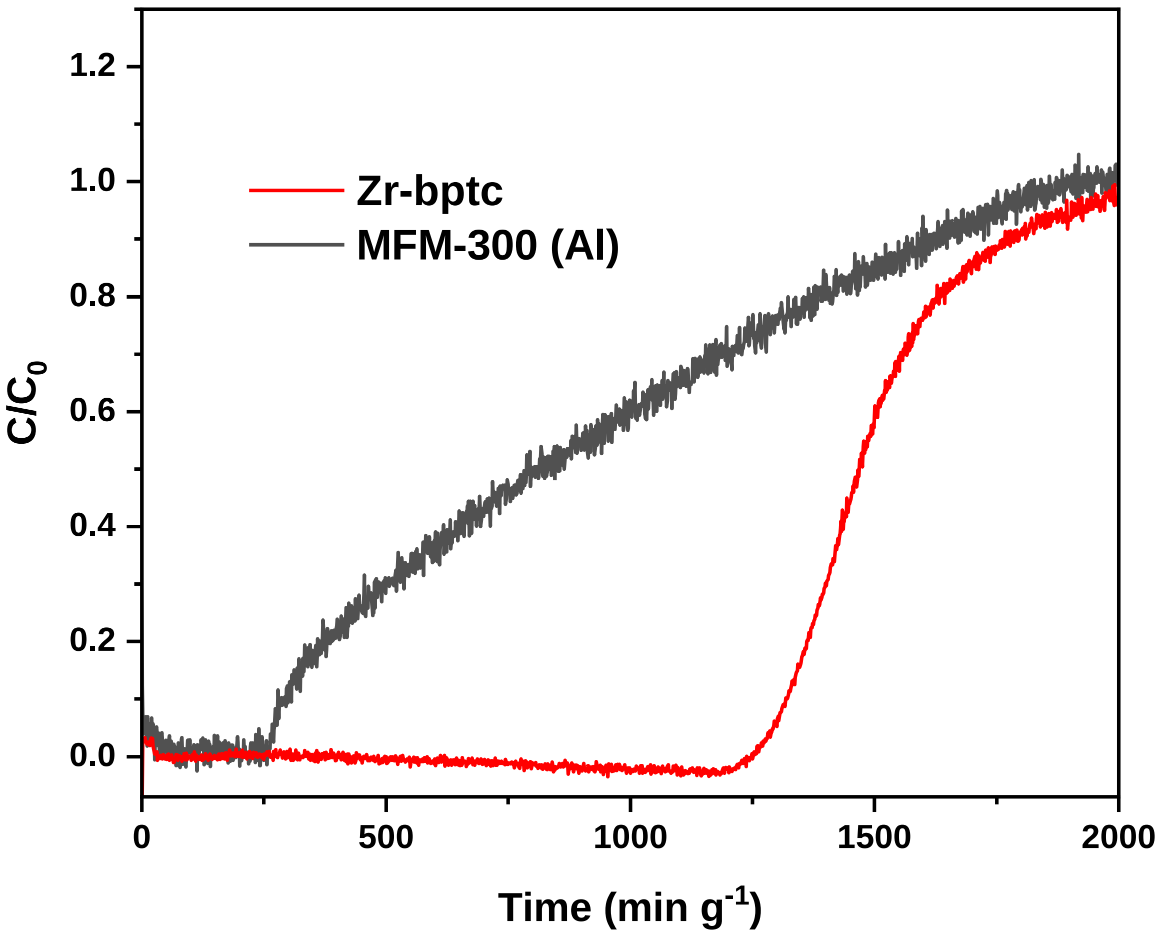

Figure S7. Comparison of breakthrough plots of MFM-300(Al) and Zr-bptc (2500 ppm NO_2 diluted in He and N_2, total flow rate 40 mL/min).

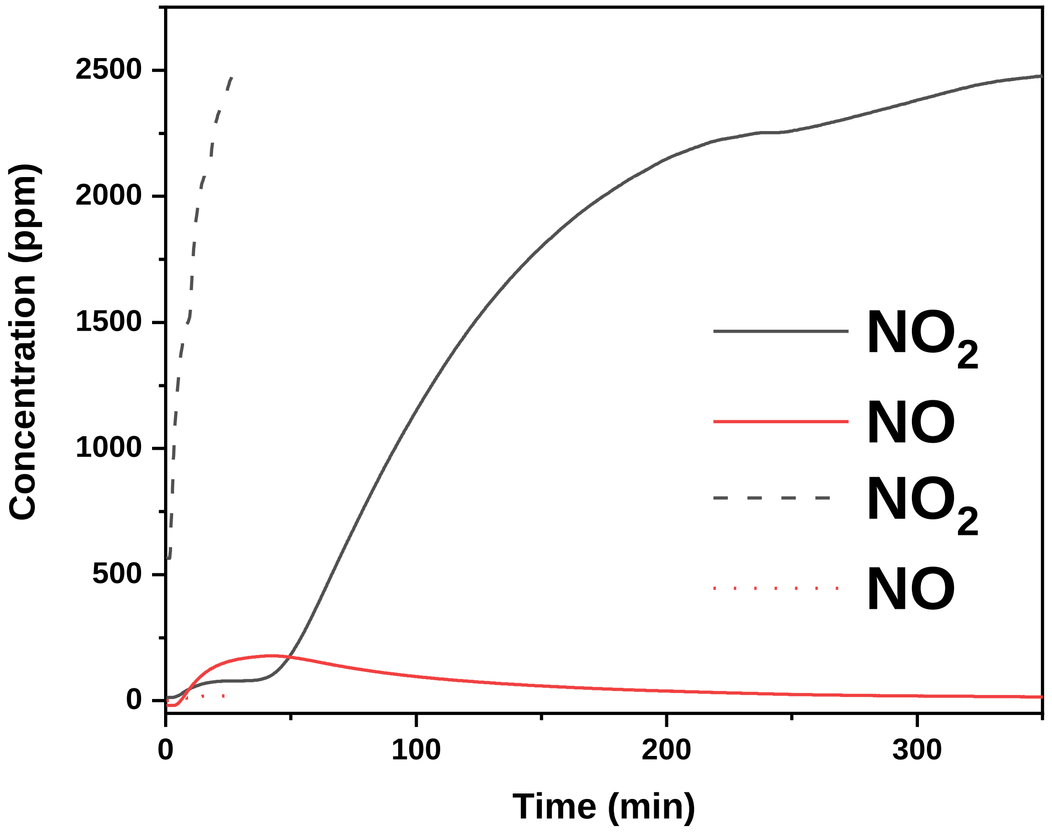

Figure S8. Breakthrough plots for NO_2 (2500 ppm NO_2 diluted in He) through a fixed-bed packed with Zr-bptc (solid line) and a blank fixed-bed (dash line). A low concentration of NO (~18 ppm) is observed upon adsorption of NO_2 and the concentration of NO decreases to the baseline upon breakthrough of NO_2.

4. Thermogravimetric Analytical Data

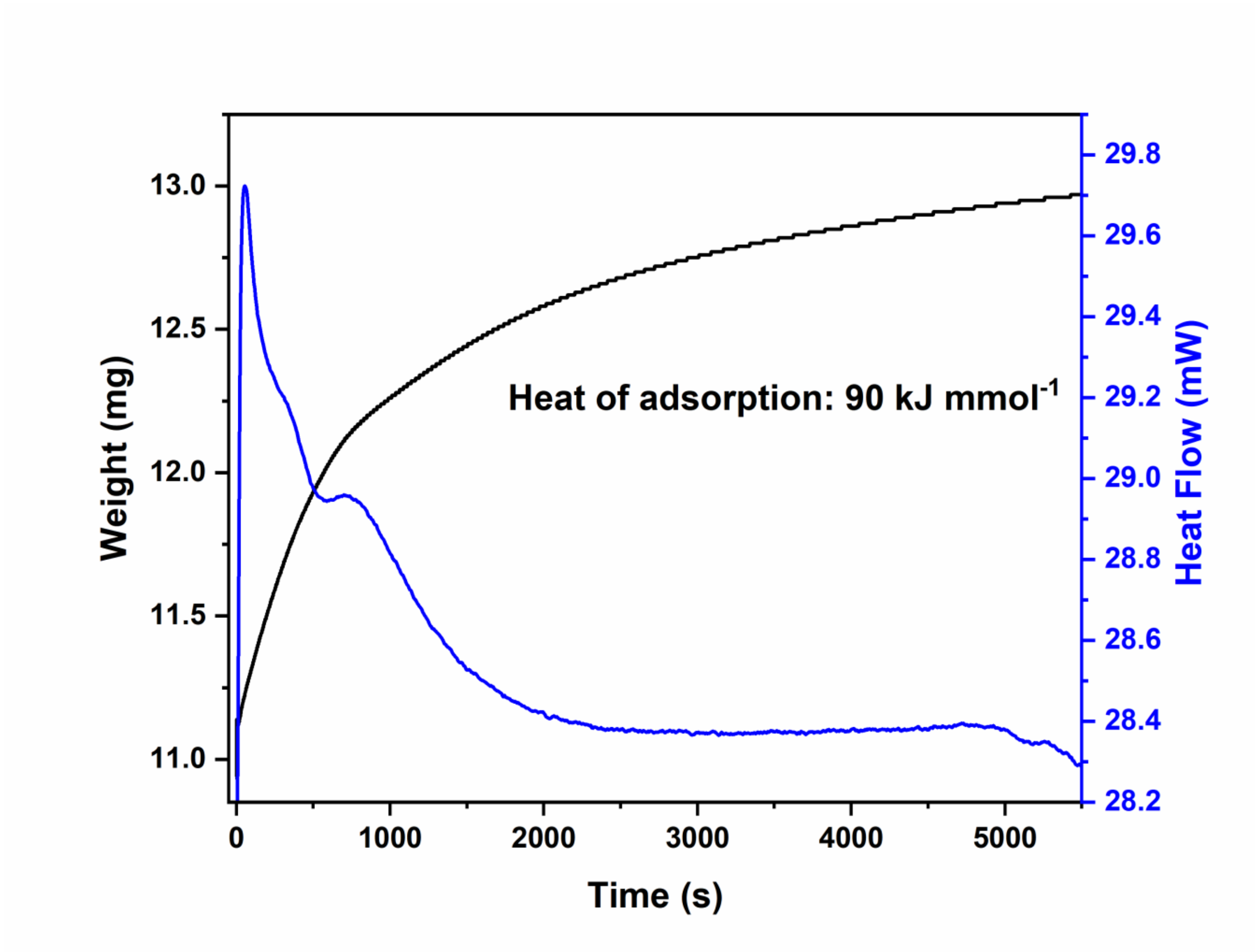

Figure S9. The TGA-DSC analysis of NO_2 adsorption in Zr-bptc (weight, black; heat flow, blue).

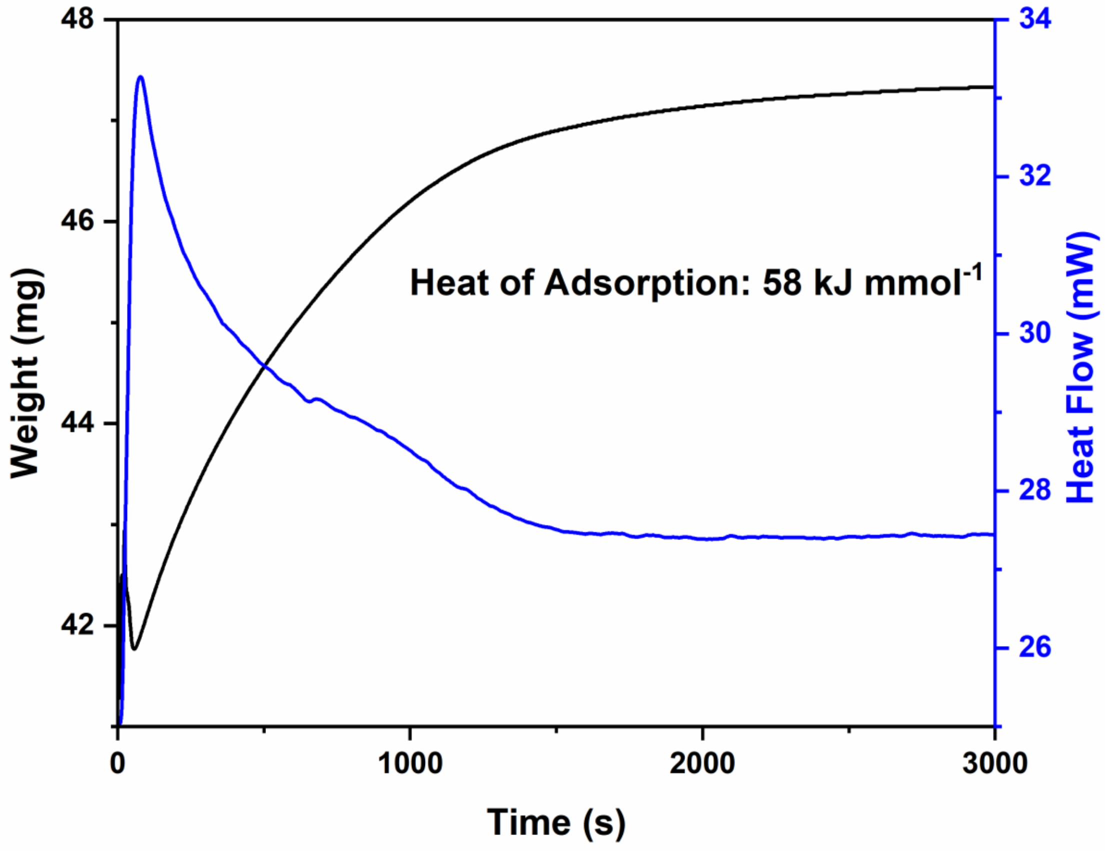

Figure S10. TGA-DSC analysis of NO_2 adsorption in MFM-520 (weight, black; heat flow, red).

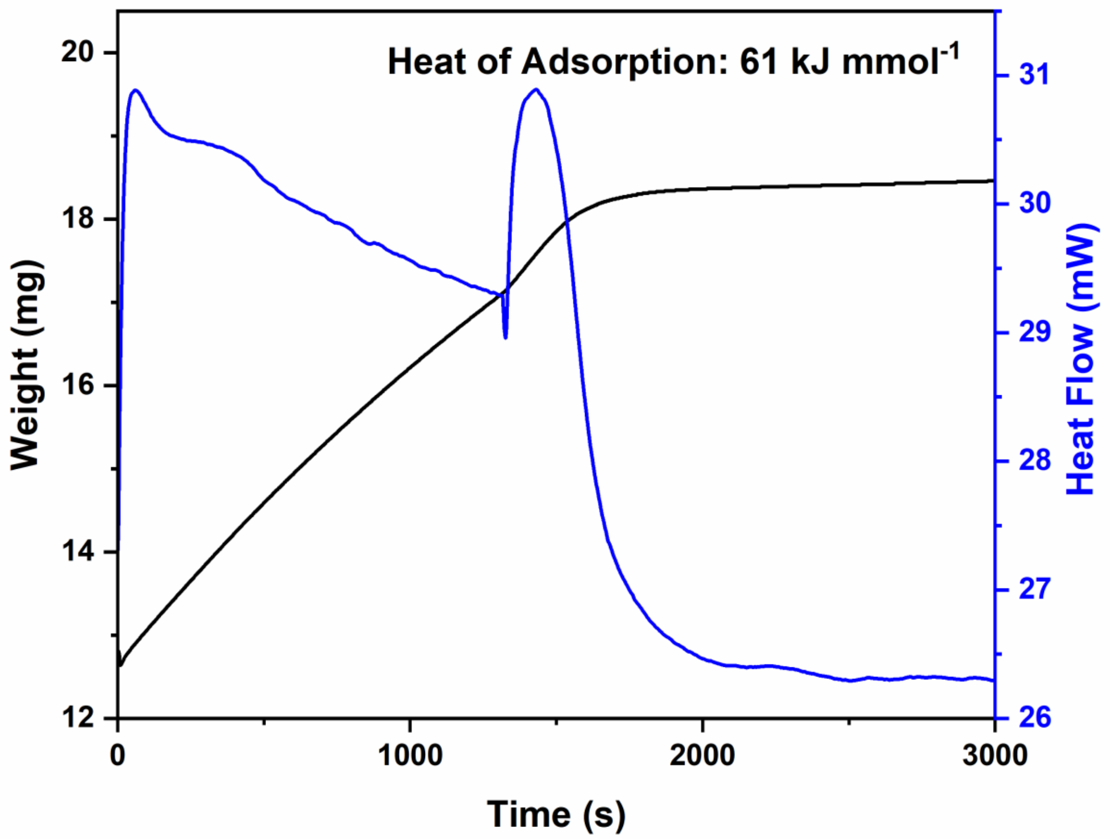

Figure S11. TGA-DSC analysis of NO_2 adsorption in MFM-300 (Al) (weight, black; heat flow, red).

5. Calculation of Isosteric Heats of Adsorption

To estimate the differential enthalpies (ΔH_n) and (ΔS_n) for CO_2 adsorption, all isotherms at different temperatures were fitted to the van't Hoff isochore:

$$\ln(P) = \frac{\Delta Hn}{RT} - \frac{\Delta Sn}{R}$$

where P is pressure, T is the temperature, R is the real gas constant. Selected linear fitting plots are shown in Figure S13. All linear fittings show R^2 above 0.98, indicating consistency in the isotherm data. A plot of ln(p) versus 1/T at constant amount adsorbed allows the differential enthalpy and entropy of adsorption and the isosteric enthalpy of adsorption (Q_{st}) to be determined.

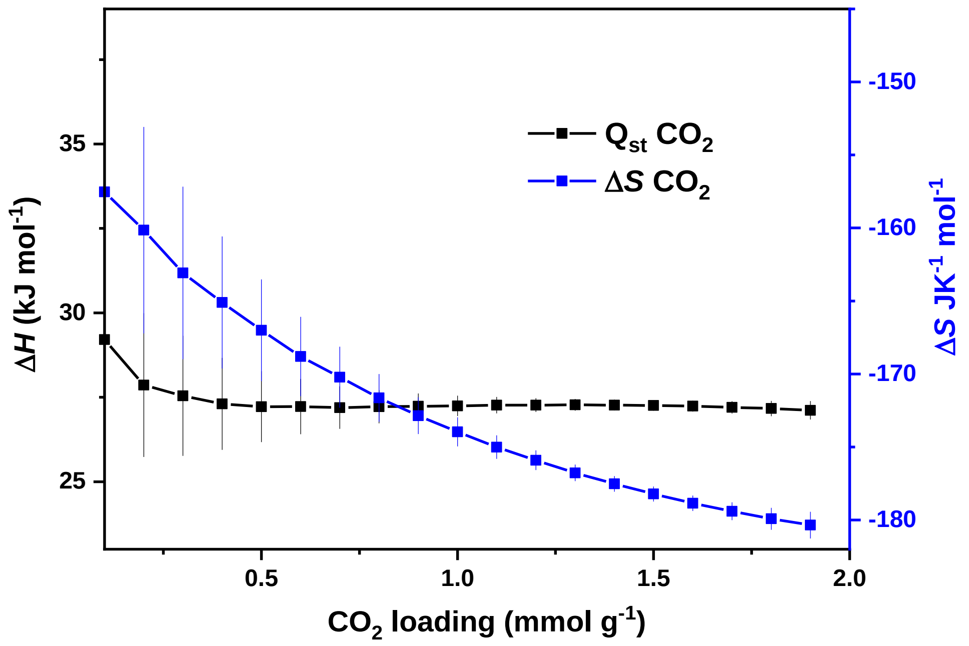

Figure S12. Variation of isosteric heat of adsorption (Q_{st}) and entropy (ΔS) for CO_2 uptake in Zr-bptc.

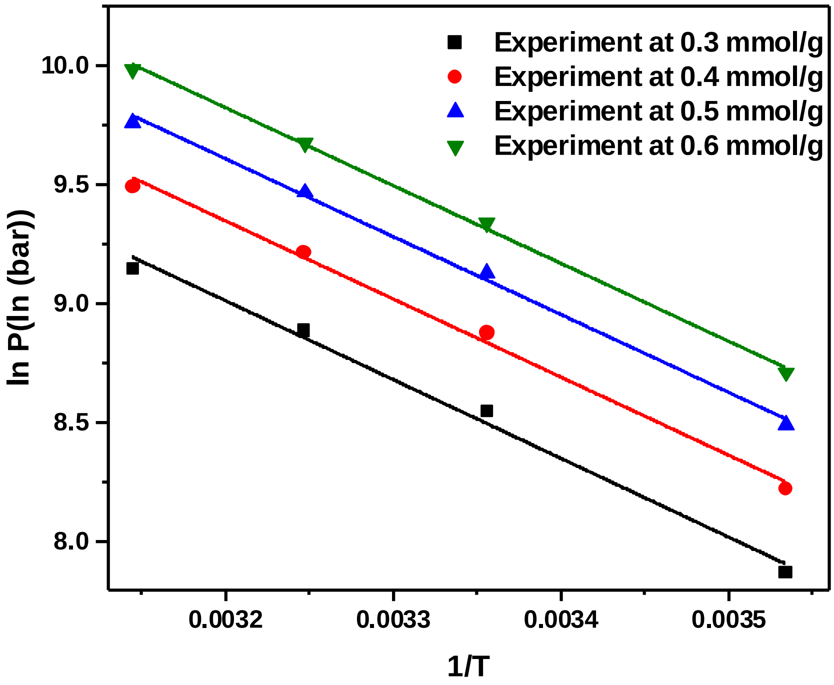

Figure S13. Linear fitting of van't Hoff plots for isotherm data for CO_2 adsorption in Zr-bptc (selected fittings show R^2 above 0.98, indicating consistency in the isotherm data).

Table S2 Thermodynamic parameters for CO_2 adsorption in Zr-bptc

n<br>mmol g^{-1}	Q_{st}<br>kJ mol^{-1}	Q_{st} error<br>kJ mol^{-1}	ΔS<br>JK^{-1} mol^{-1}	ΔS error<br>JK^{-1} mol^{-1}	R^2
0.1	29.2	2.0	-157.5	-6.6	0.9864
0.2	27.9	2.1	-160.1	-7.1	0.9827
0.3	27.5	1.8	-163.1	-5.9	0.9876
0.4	27.3	1.4	-165.1	-4.5	0.9926
0.5	27.2	1.0	-167.0	-3.5	0.9956
0.6	27.2	0.8	-168.8	-2.7	0.9973
0.7	27.2	0.6	-170.2	-2.1	0.9984
0.8	27.2	0.5	-171.6	-1.6	0.9990
0.9	27.2	0.4	-172.9	-1.3	0.9994
1.0	27.2	0.3	-174.0	-1.0	0.9996
1.1	27.3	0.2	-175.0	-0.8	0.9998
1.2	27.3	0.2	-175.9	-0.7	0.9998
1.3	27.3	0.2	-176.8	-0.6	0.9999
1.4	27.3	0.2	-177.5	-0.5	0.9999
1.5	27.3	0.2	-178.2	-0.5	0.9999
1.6	27.2	0.2	-178.9	-0.5	0.9999
1.7	27.2	0.2	-179.4	-0.6	0.9999
1.8	27.2	0.2	-179.9	-0.7	0.9998
1.9	27.1	0.3	-180.3	-0.9	0.9997

6. Selectivity Data for Zr-bptc

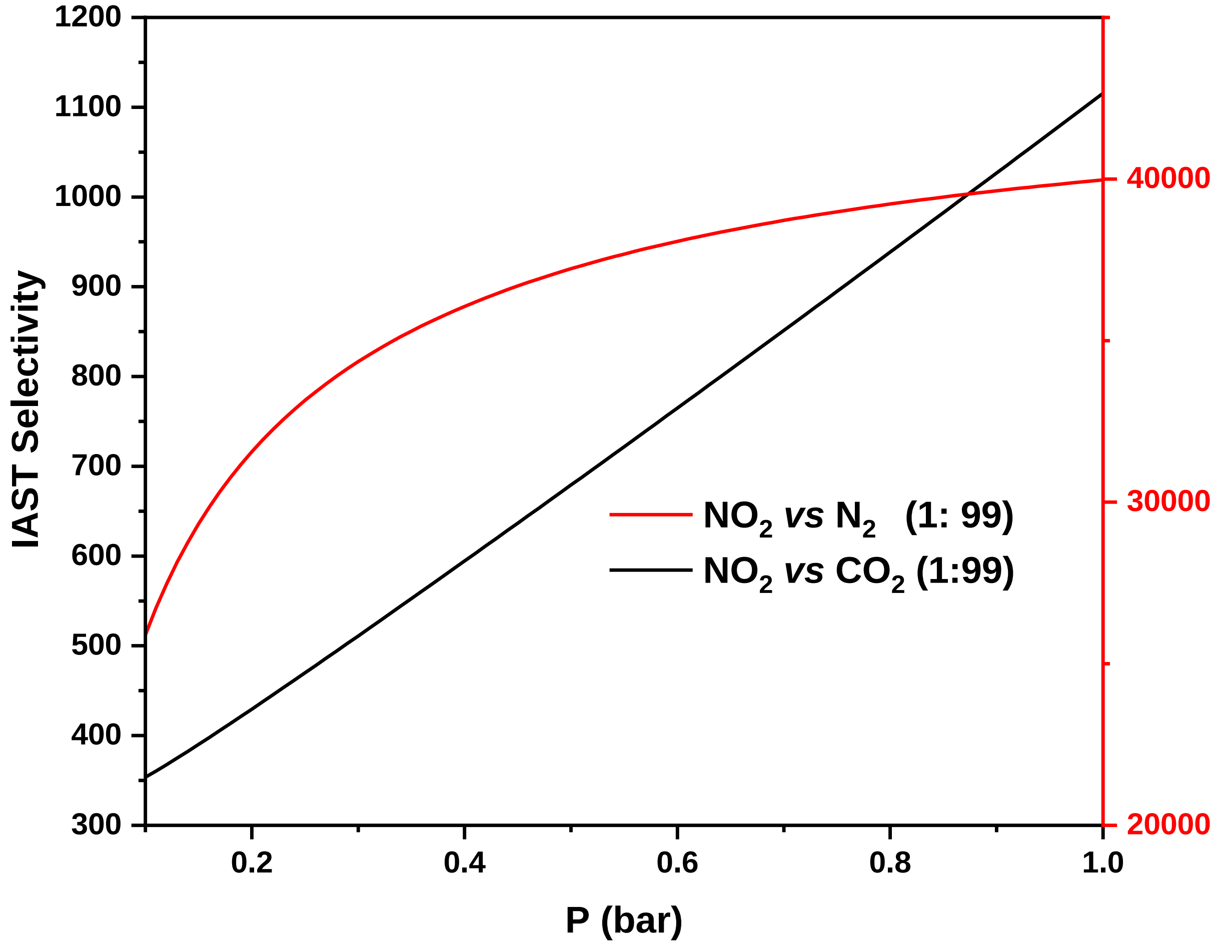

Figure S14. IAST selectivities for mixtures of NO_2/CO_2 (1:99) and NO_2/N_2 (1:99) and at 0.1–1.0 bar for Zr-bptc at 298 K.

7. Additional Structural Views and Data on Gas-loaded Zr-bptc

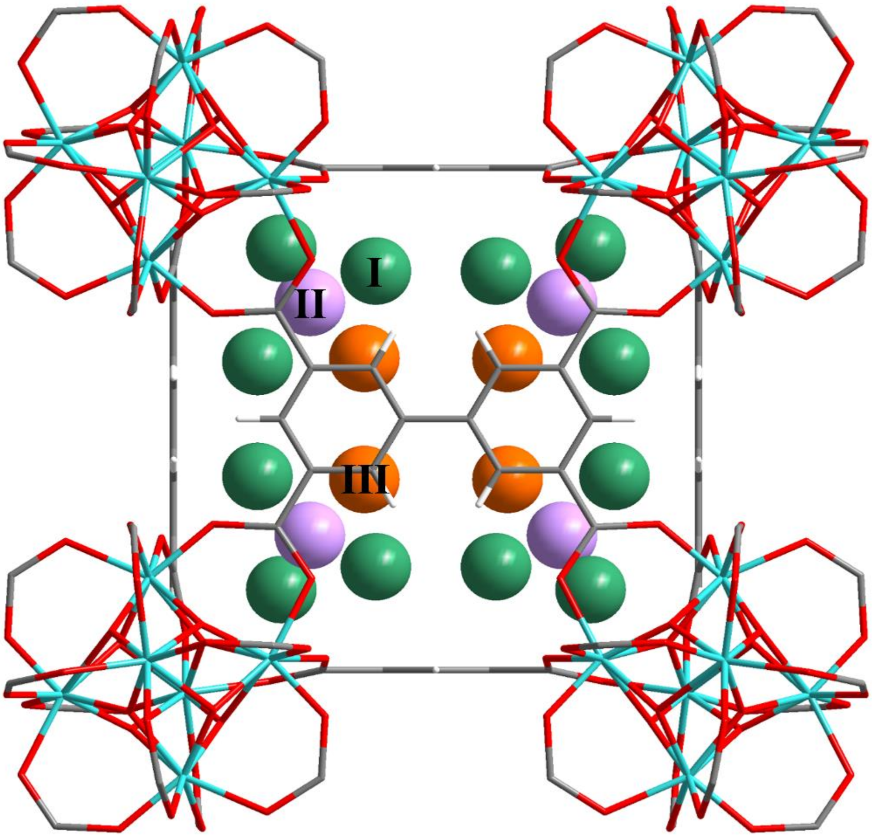

Figure S15. Binding sites of NO_2 in Zr-bptc (sea green: site I; pink: site II and orange: site III).

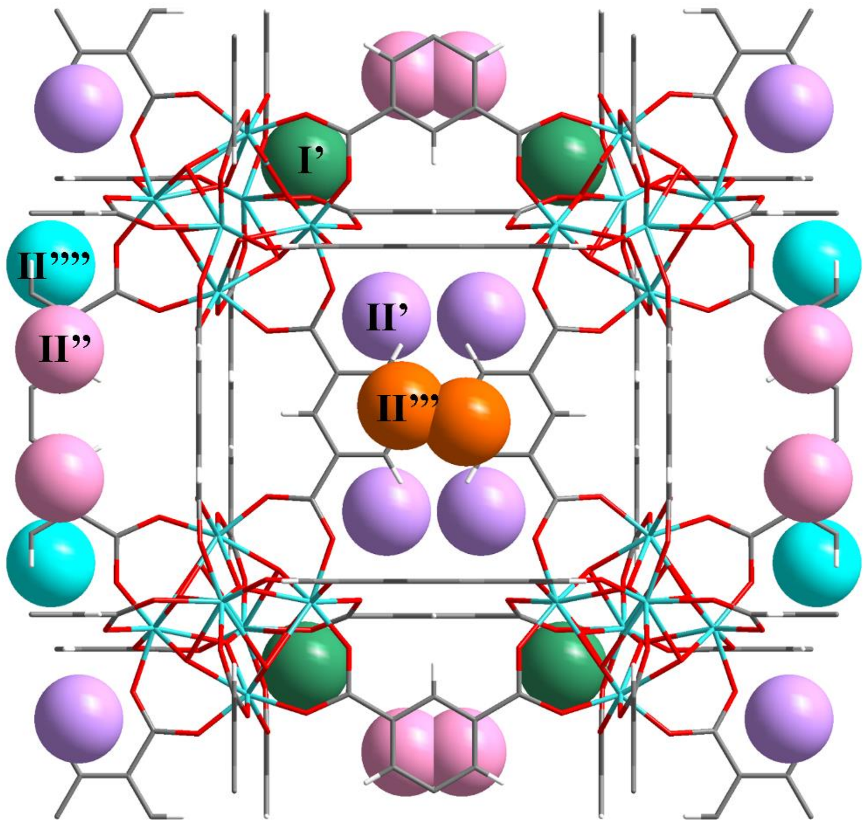

Figure S16. Binding sites of CO_2 in Zr-bptc (sea green: site I’; pink: site II’; rose: site II’’; orange: II’’’; cyan: II’’’’). The CO_2 molecules at site II’ are comprised of four closely related binding sites, namely II’, II’’, II’’’ and II’’’’, due to the reduced crystal symmetry from cubic to orthorhombic system owing to a minor distortion.

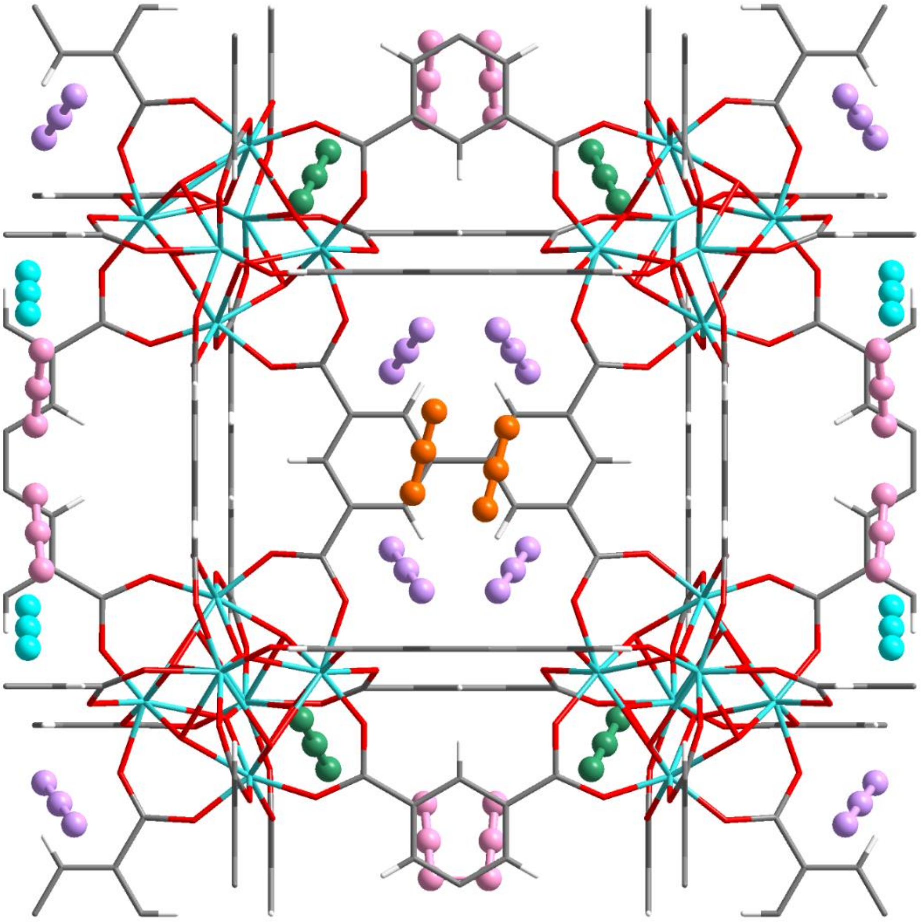

Figure S17. Packing of adsorbed CO_2 molecules in $Zr_6O_4(OH)_4(bptc)_3{\cdot}(CO_2)_{2.8}$.

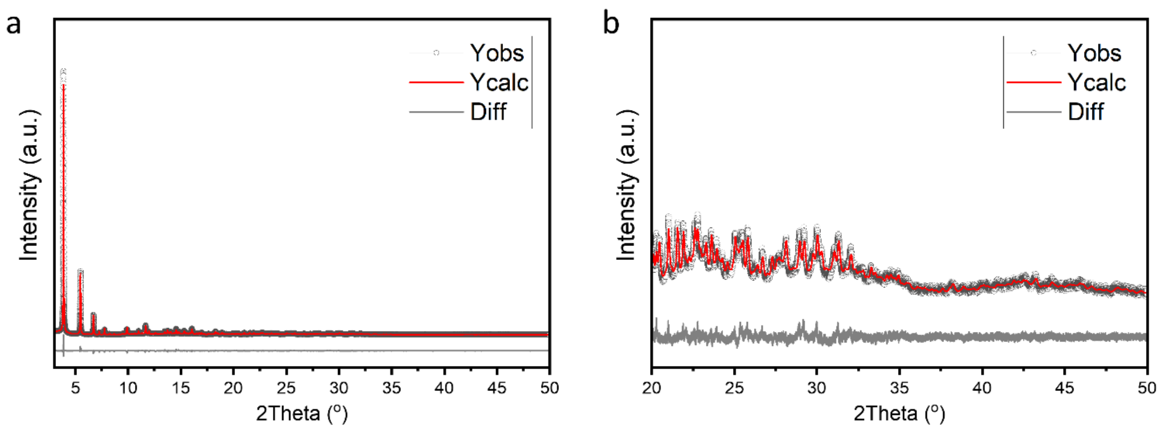

Figure S18. PXRD patterns [observed (black), calculated (red) and difference (grey)] for the Rietveld refinement of the bare Zr-bptc [$\lambda = 0.8264(6)$ Å]; (a) view of the fitting of PXRD patterns ($2\theta = 3\text{-}50^o$); (b) high angle data ($2\theta = 20\text{-}50^o$) scaled up to show the quality of fit between the observed and the calculated patterns.

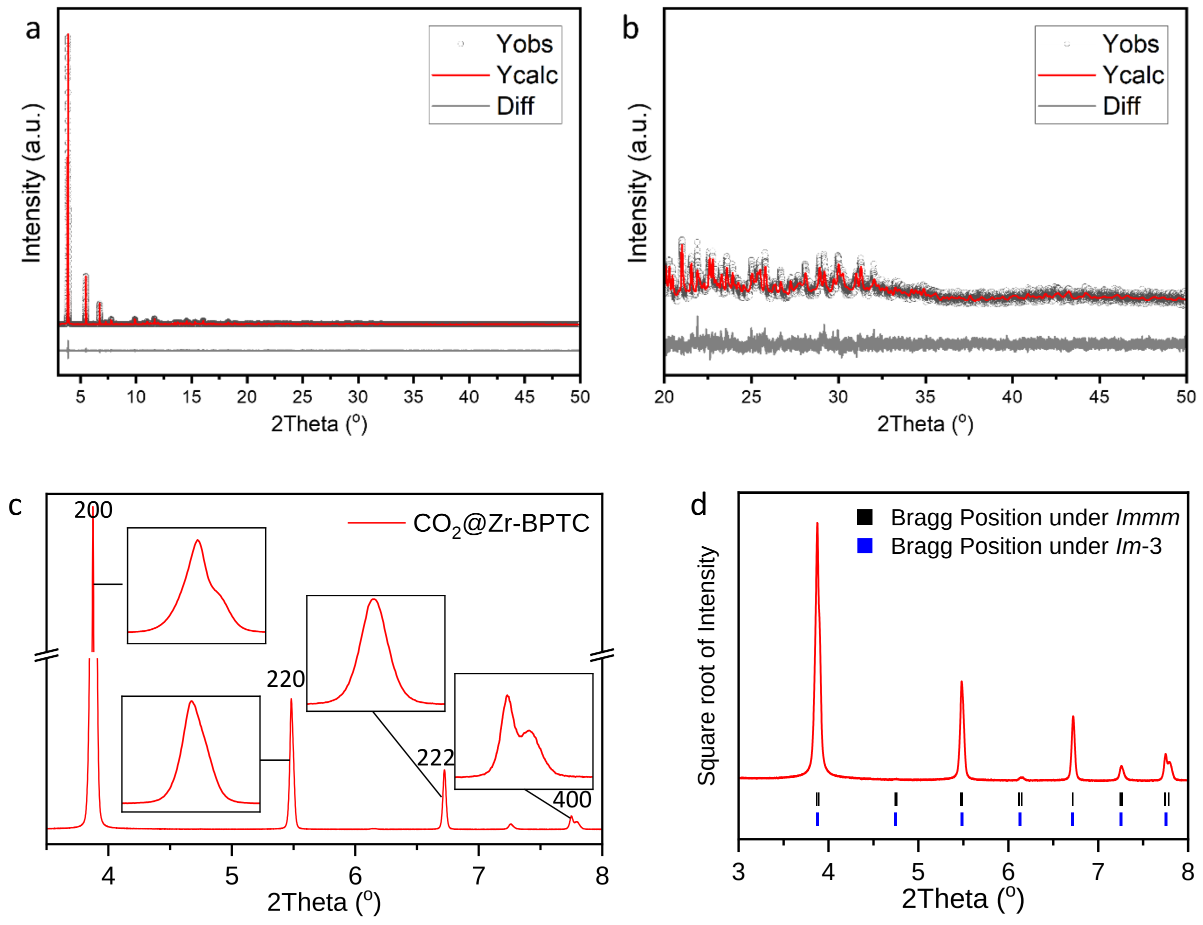

Figure S19. PXRD patterns [observed (black), calculated (red) and difference (grey)] for the Rietveld refinement of the $\{Zr_6O_4(OH)_4(bptc)_3{\cdot}(CO_2)_{2.8}\}$ [λ = 0.8245(2) Å]; (a) view of the fitting of PXRD patterns (2θ = 3-50°); (b) high angle data (2θ = 20-50°) scaled up to show the quality of fit between the observed and the calculated patterns; (c) detailed view of PXRD pattern 2θ = 3-8°; (d) comparison of Bragg positions in space group of *I*mmm and *I*m-3 for $\{Zr_6O_4(OH)_4(bptc)_3{\cdot}(CO_2)_{2.8}\}$ confirming a reduction of symmetry upon CO_2 loading. It is worth noting that there is no structural change in coordination at the metal sites or metal-ligand connectivity upon the reduction of symmetry except a slight compression of the lattice along the *b*-axis.

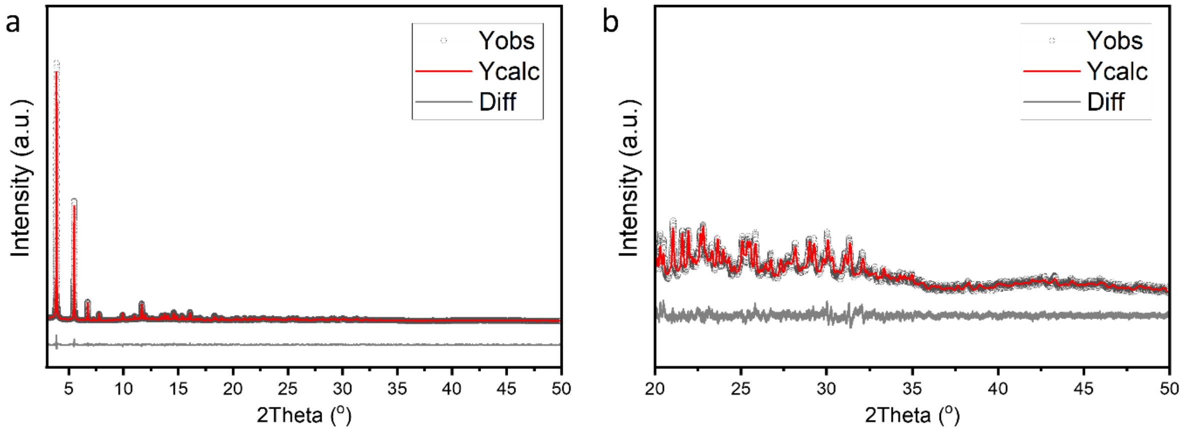

Figure S20. PXRD patterns [observed (black), calculated (red) and difference (grey)] for the Rietveld refinement of the $Zr_6O_4(OH)_4(bptc)_3{\cdot}(NO_2)_{7.5}{\cdot}(NO_2)_{2.3}{\cdot}(N_2O_4)_{4.1}$ [λ = 0.8267(6) Å]; (a) view of the fitting of PXRD patterns (2θ = 3-50°); (b) high angle data (2θ = 20-50°) scaled up to show the quality of fit between the observed and the calculated patterns.

Table S3. Summary of powder X-ray diffraction refinements for gas loaded Zr-bptc

	Zr-bptc	NO_2@Zr-bptc	CO_2@Zr-bptc
Formula	$Zr_6C_{48}H_{22}O_{32}\cdot(H_2O)_{16.83}$	$Zr_6C_{48}H_{22}O_{32}\cdot(NO_2)_{7.5}\cdot(NO_2)_{2.3}\cdot(N_2O_4)_{4.1}$	$Zr_6C_{48}H_{22}O_{32}\cdot(CO_2)_{2.8}$
Formula weight (g/mol)	1923.20	2486.11	2322.01
Temp (K)	298	298	298
Radiation type		Synchrotron	
Diffractometer		Beamline I11 of Diamond Light Source	
Data collection mode		Transmission	
Wavelength (Å)	0.826452(6)	0.826452(6)	0.8245(2)
Crystal system	cubic	cubic	orthorhombic
Space group	Im-3	Im-3	Immm
a / Å	24.4047(5)	24.3731(3)	24.39(6)
b / Å	24.4047(5)	24.3731(3)	24.22(6)
c / Å	24.4047(5)	24.3731(3)	24.39(4)
α/ β/ γ	90	90	90
V / $Å^3$	14,535.2(8)	14,478.7(1)	14,417.3(3)
D_c g/cm^3	1.512	2.287	1.615
R_{exp} / %	2.332	2.148	6.639
R_{wp} / %	6.110	5.905	10.407
R_p / %	4.412	4.279	7.044
GoF	2.619	2.749	1.615
R_{Bragg}	2.781	2.122	2.935

Table S4. Summary of O···H-C supramolecular interactions in NO_2@Zr-bptc and CO_2@Zr-bptc

	Types	N<u>O</u>$_2$···<u>H</u>-C distance (Å)	$< C - H - O$ (°)
NO_2@Zr-bptc	Electronic binding	2.00(7)	101.8(4)
	Hydrogen binding	2.36(3)	144.4(7)
	Hydrogen binding	2.91(8)	156.5(7)
		N_2<u>O</u>$_4$···<u>H</u>-C distance (Å)	
	Electronic binding	2.03(6)	77.2(6)
		C<u>O</u>$_2$···<u>H</u>-C distance (Å)	
CO_2@Zr-bptc	Hydrogen binding	2.38(2)	147.3(0)
		2.38(9)	133.3(8)
		2.65(6)	120.4(9)
		3.16(5)	139.3(2)
		3.27(3)	149.7(1)
		3.47(5)	165.6(5)

8. Additional Analysis of EPR Data

ENDOR spectra show hyperfine interactions between the NO_2-based electron spin and ^1H nuclear spins of the interior of the MOF. The simulated model was calculated based on the dipolar (through space) interactions between the NO_2 and the nearest ^1H nucleus. The 3x3 dipolar ^1H hyperfine interaction matrices (A^{dip}) were calculated according to:

$$A^{dip} = \frac{\mu_0}{4\pi h}\beta_e\beta_n\sum_k \rho_k \frac{3(\mathrm{g.\,n_k})(\tilde{\mathrm{n}}_\mathrm{k}.\,g_n 1) - \mathrm{g}.\,g_n 1}{r_k^3}$$

where h is Plank's constant ($6.63\text{x}10^{-34}$ J.s), μ_0 is vacuum permittivity ($1.26\text{x}10^{-6}$ $T^2.J^{-1}.m^3$), β_e is Bohr magneton ($9.27\text{x}10^{-24}$ $J.T^{-1}$) and β_n is nuclear magneton ($5.05\text{x}10^{-27}$ $J.T^{-1}$), respectively. The nuclear *g*-values for ^1H: $g_H = 5.586$. g and g_n1 are the electron and nuclear g (3x3) matrixes (g_n is the nuclear *g*-value, a scalar; 1 is the unit matrix), ρ_k is the electron spin population at atom k ($0 \leq \rho_k \leq 1$ and dimensionless), n_k is the $n\ldots k$ unit vector expressed in the molecular frame and r_k is the $n\ldots k$ distance ($O_2\underline{N}\cdots H$).

Calculations, based on a dipolar model, give a $O_2\underline{N}\cdots H$ distance of 3.8 Å. For the observed linewidth, the simulated spectra show low sensitivity to the polar angles between the N-H vector and *g*-tensor frame. This longer N-H distance compared with PXRD model suggests that the intermolecular interaction between the NO_2 molecules are stronger at low temperature.

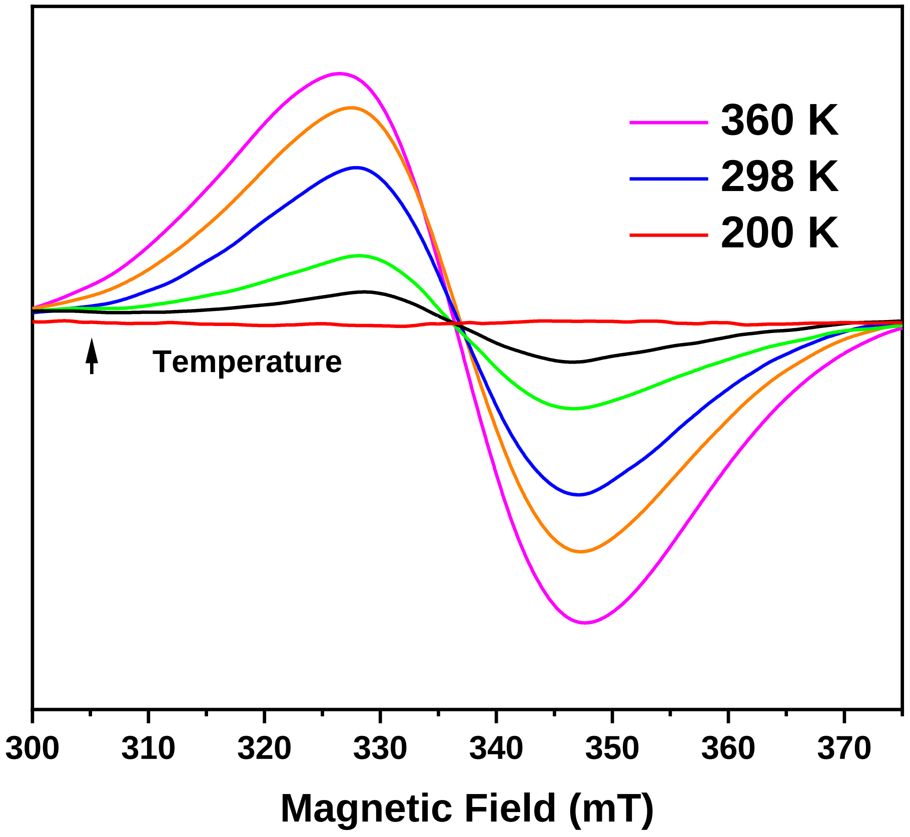

Figure S21. Temperature dependence of the X-band EPR spectra of NO_2-loaded Zr-bptc between 200 and 360 K (red: 200 K, black: 250 K, green: 270 K, blue: 298 K, orange: 310 K and magenta: 360 K).

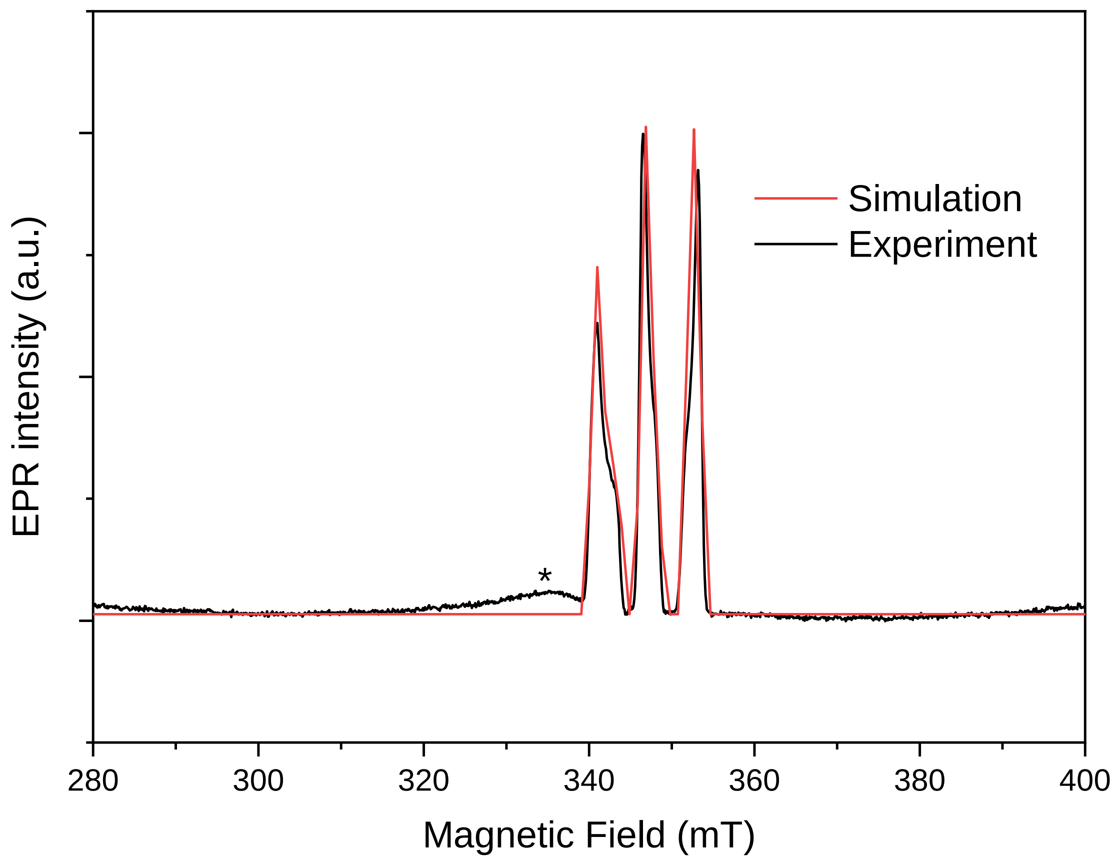

Figure S22. X-band (9.731622 GHz) EDFS spectrum of NO_2-loaded Zr-bptc at 5.7 K. The signal '*' at *ca.* 330 mT is from the cavity.

Table S5. Linewidth of cw NO_2 EPR spectra in different MOF systems.

	Gaussian Linewidth	Lorentzian Linewidth
Zr-bptc	0.4	0.4
MFM-300(Al)	0.22	0.22

9. Conversion of Captured NO_2 in Zr-bptc

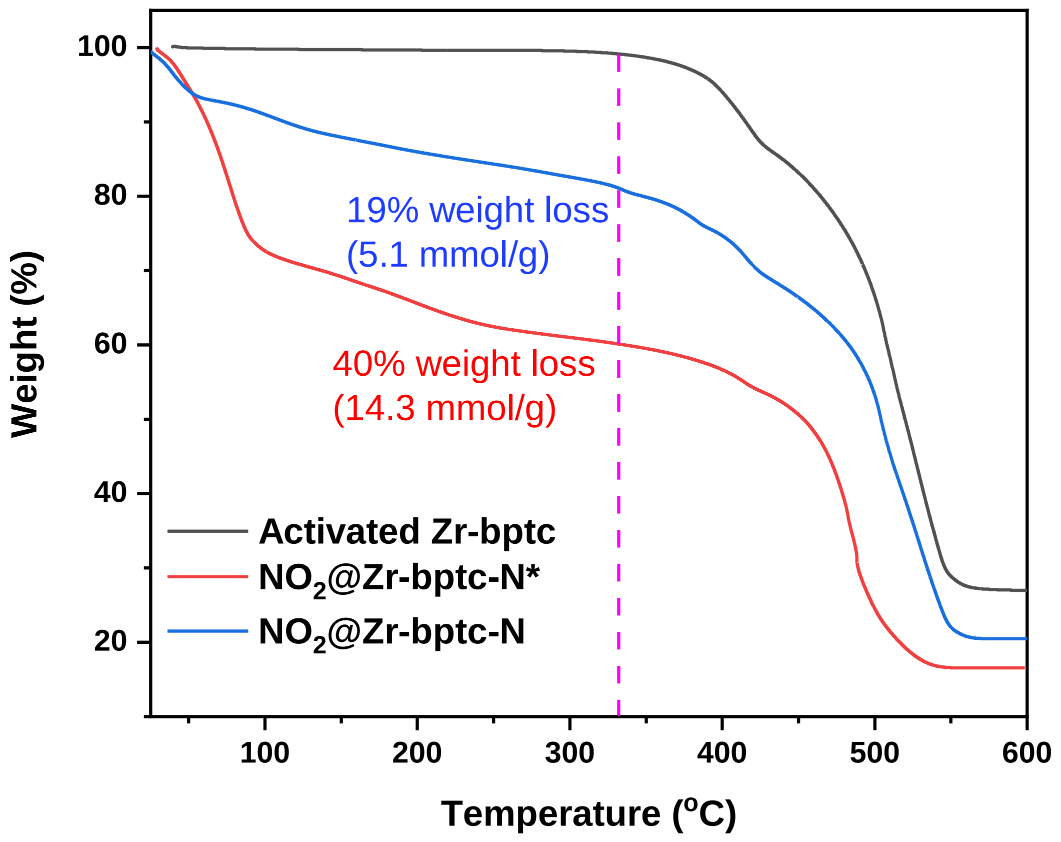

Figure S23. TGA plot of activated Zr-bptc, NO_2@Zr-bptc-N* (NO_2 loaded at 1 bar, 298 K) and NO_2@Zr-bptc-N (NO_2 loaded at 2500 ppm, 298 K). Weight losses of 19% and 40% are observed for NO_2@Zr-bptc-N and NO_2@Zr-bptc-N*, respectively. This corresponds to NO_2 capacities of 5.1 and 14.3 mmol g^{-1}, respectively; these are consistent with the values obtained respectively by breakthrough experiments (4.9 mmol g^{-1}) and isotherm adsorption measurements at 1 bar (13.8 mmol g^{-1}). The temperature was increased at a rate of 5 °C/min under air.

X-band EPR spectroscopy was used to confirm the full conversion of captured NO_2 in the nitration reactions. Phenol, anisole and toluene were selected to study conversion of captured NO_2. The EPR spectrum of NO_2@Zr-bptc-N in $CHCl_3$ was measured, and the substrate was then added. Upon completion of reaction, the EPR spectra of the reaction mixture and the supernatant of the reaction mixture were measured to confirm absence or presence of residual NO_2 and thus determine the level of conversion of captured NO_2 (Figure S24).

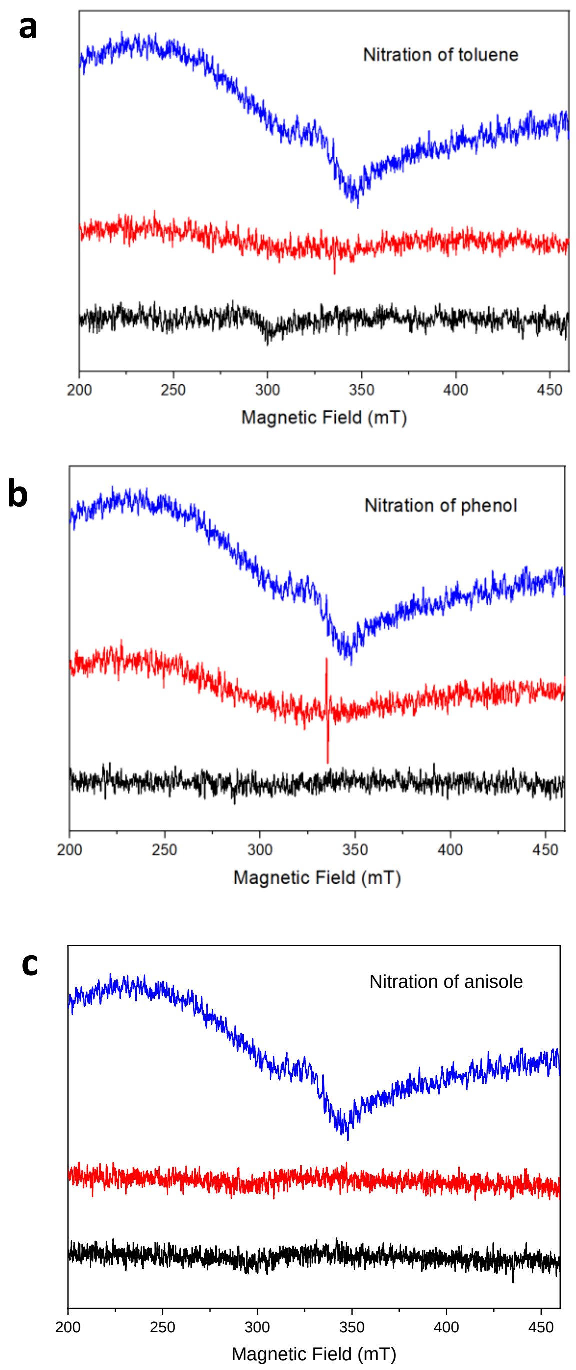

Figure S24. CW X-band EPR spectra of NO_2@ Zr-bptc-N/$CHCl_3$ mixture (blue), reaction mixture (red) and supernatant of the reaction mixture (black) in the nitration of toluene (a), phenol (b) and anisole (c) at room temperature.

X-band EPR spectroscopy was used to investigate the diffusion of NO_2 from the pore of Zr-bptc into the reaction mixture. $CHCl_3$ (5 mL) was added to NO_2@Zr-bptc-N (100 mg) under vigorous stirring for 5 minutes. Half of this mixture was transferred for EPR measurements, and the other half was filtered and the EPR spectrum of the supernatant measured. Similar intensities for the NO_2 signals are observed for these two EPR measurements (Figure S25). Thus, at the conversion stage, the captured NO_2 molecules by Zr-bptc diffuse into the reaction mixture to drive the nitration reaction.

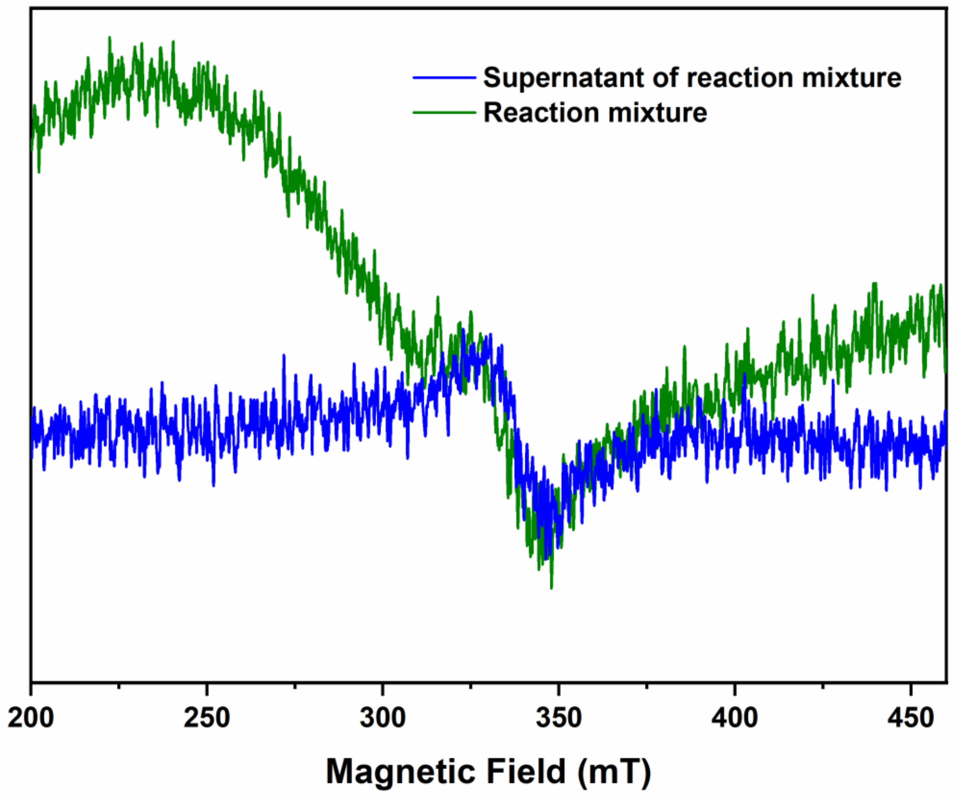

Figure S25. CW X-band EPR spectra of NO_2@Zr-bptc-N/$CHCl_3$ mixture (green) and supernatant of NO_2@ Zr-bptc-N/$CHCl_3$ mixture (blue) at room temperature (the large feature in the spectrum of MOF-containing mixture originates from trace impurities in Zr-bptc).

Procedure for conversion in control experiments

A flow of 2500 ppm NO_2 (diluted in N_2 and He) was passed separately through Zr-bptc (100 mg), and through metal salt ($ZrOCl_2 \cdot 8H_2O$) (100 mg) and through H_4bptc (H_4bptc= biphenyl-3,3',5,5'-tetracarboxylic acid) (100 mg) until complete breakthrough saturation was achieved. NO_2-loaded Zr-bptc, $ZrOCl_2 \cdot 8H_2O$ and H_4bptc were then applied separately to the conversion of anisole. GC analysis was carried out on the reaction solution to determine conversion using a combination of internal and external standards.

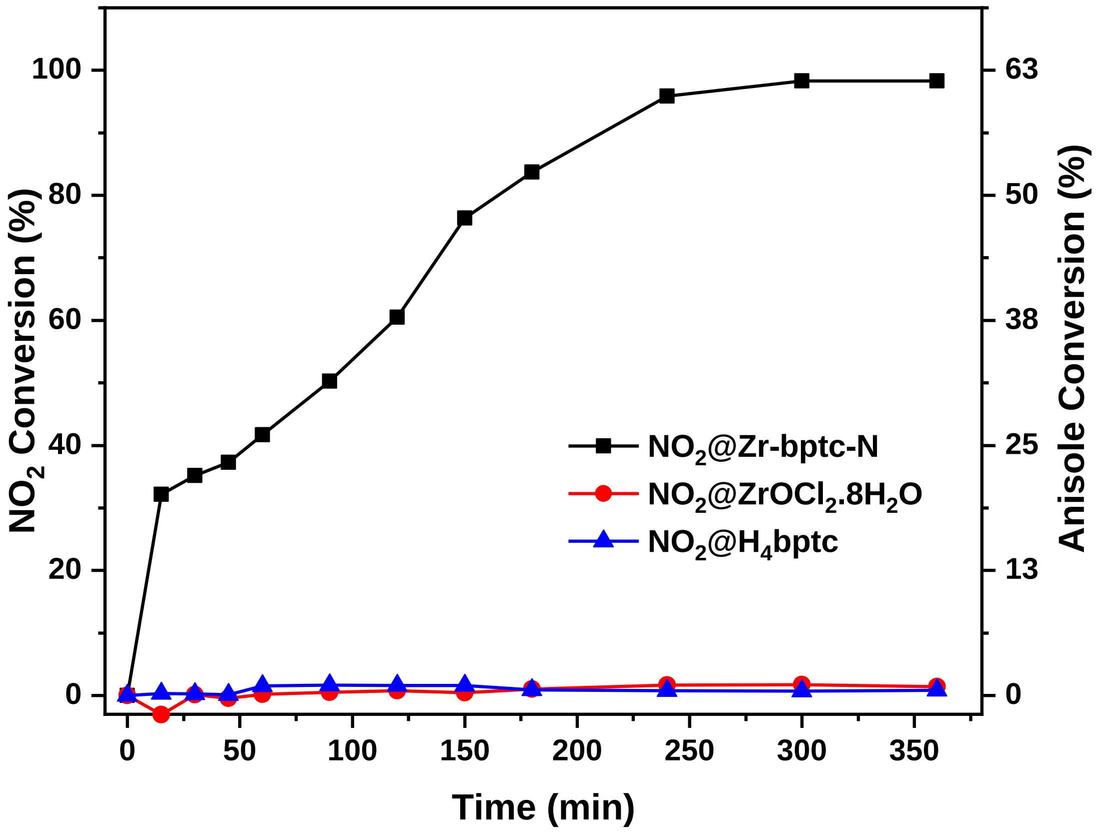

Figure S26. Plot of reaction time *vs* conversion for the nitration of anisole. Reaction conditions: anisole (0.75 mmol), NO_2-loaded solid (100 mg), *conc* H_2SO_4 (1%), $CHCl_3$ (5 mL), room temperature, 7 h. The conversion of NO_2 is based on the isolated products. No conversion was observed for reactions using NO_2-loaded $ZrOCl_2 \cdot 8H_2O$ and H_4bptc.

A pre-cleaned Schlenk flask (25 mL) with a magnetic stir bar was dosed with NO_2 at 500 mbar and 298 K (equivalent to 0.50 mmol NO_2 using ideal gas conditions). The aromatic substrate (0.75 mmol) was dissolved in $CHCl_3$ (5.0 mL) and then transferred to the flask. The reaction was conducted under the same condition with various substrates using NO_2@Zr-bptc-N. Upon completion of reaction, the mixture was analysed by GC-MS/NMR. Over-nitration and over-oxidation are observed for a number of substrates, particularly for phenol. Several factors could contribute to this observation, for example, the inaccurate control of loading of NO_2 gas to the system and high concentration of NO_2 at the liquid/gas interface.

NMR spectra of nitration products by gaseous NO_2

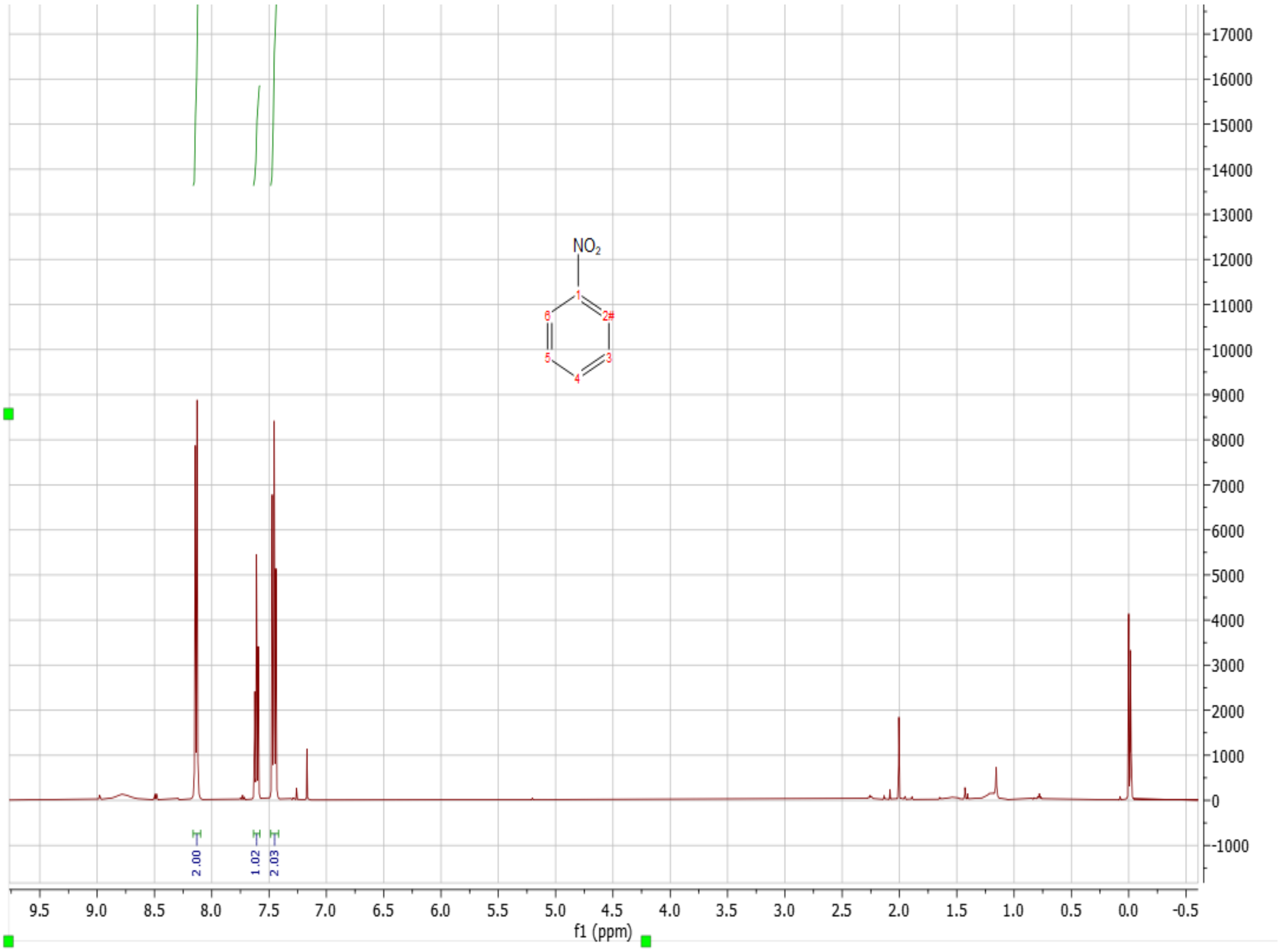

Figure S27. ^{1}H NMR spectrum of the reaction mixture of benzene with gaseous NO_2.

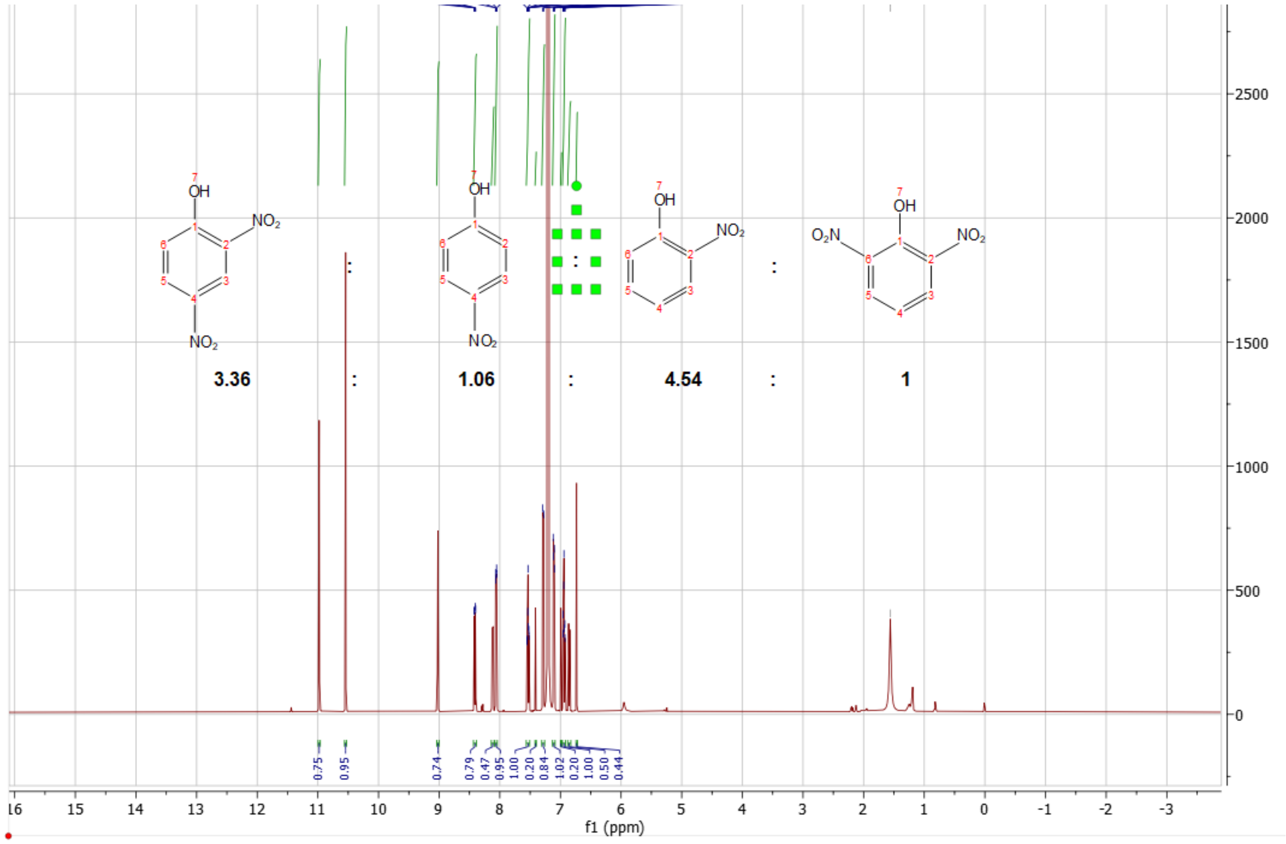

Figure S28. ^{1}H NMR spectrum of the reaction mixture of phenol with gaseous NO_2.

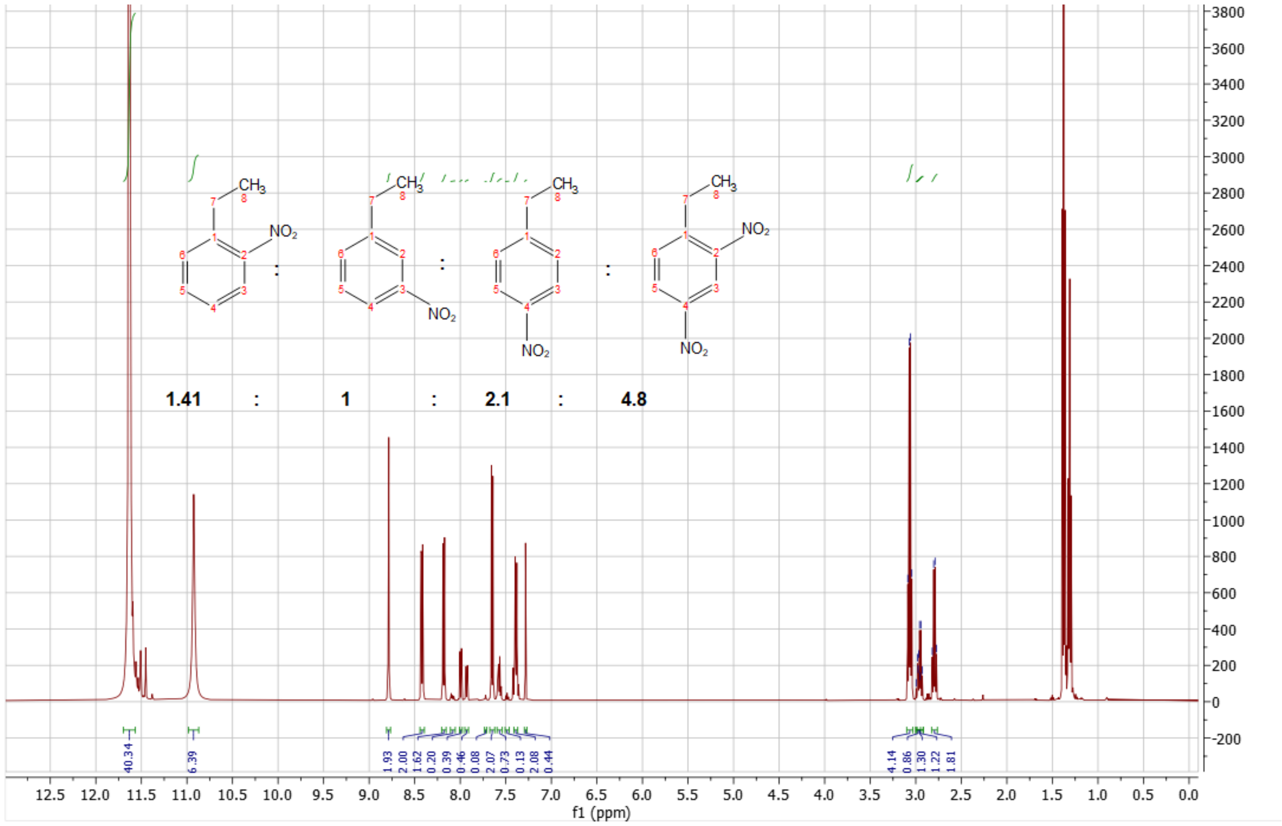

Figure S29. ^{1}H NMR spectrum of the reaction mixture of ethylbenzene with gaseous NO_2.

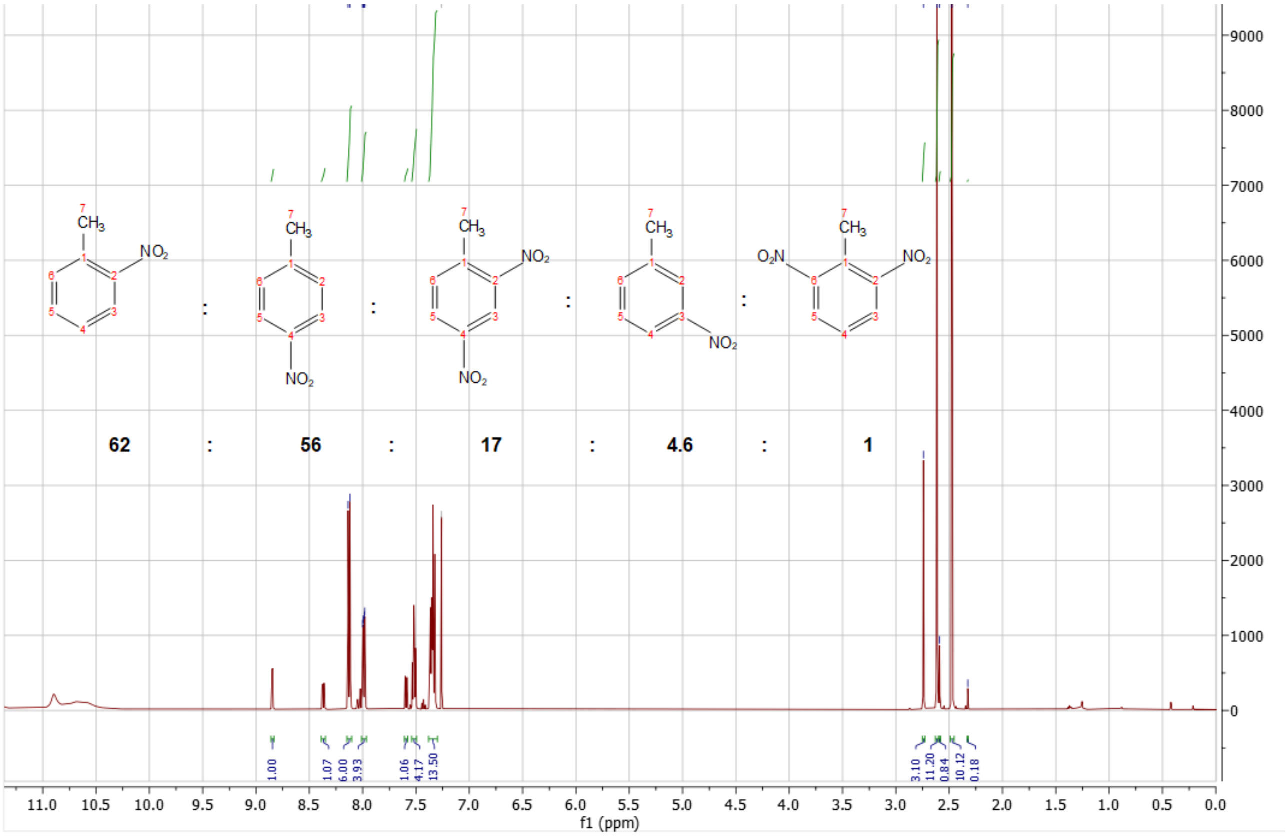

Figure S30. ^{1}H NMR spectrum of the reaction mixture of toluene with gaseous NO_2.

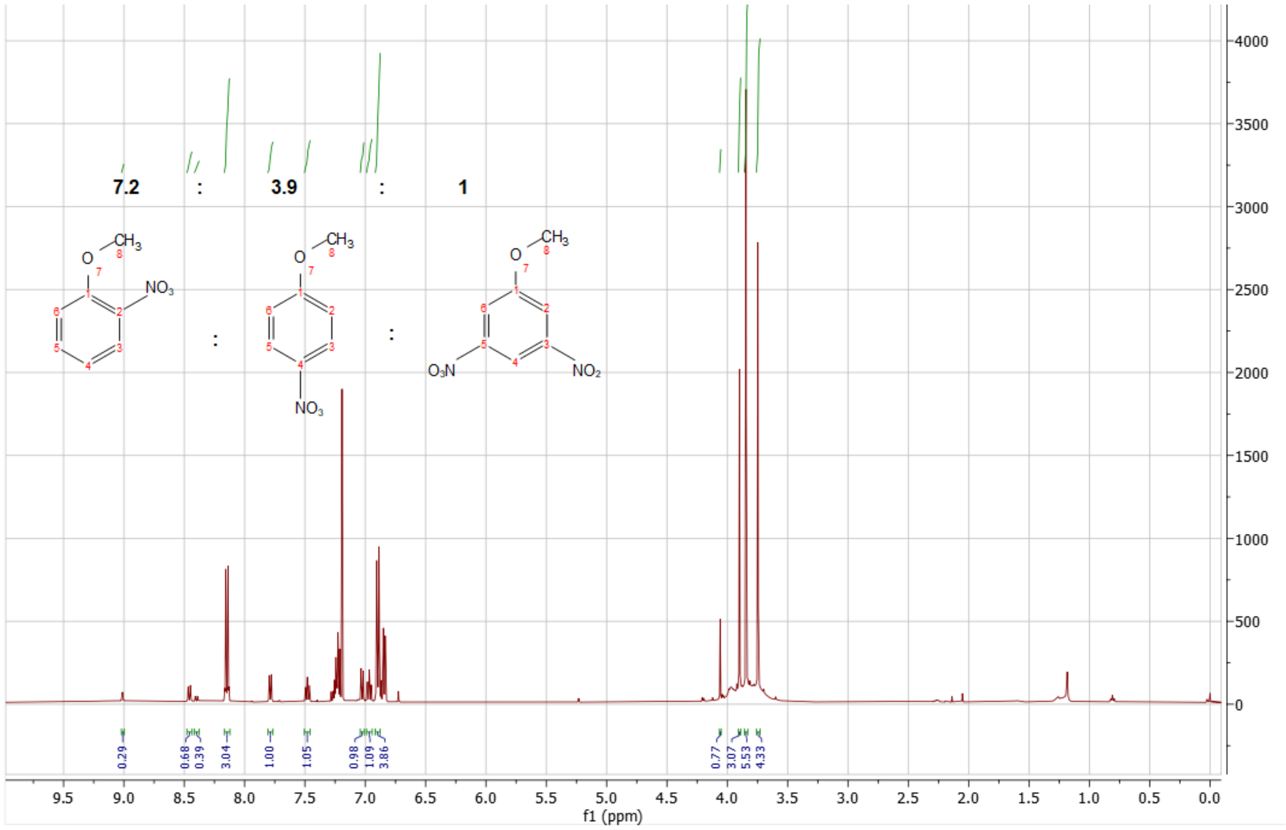

Figure S31. ^{1}H NMR spectrum of the reaction mixture of anisole with gaseous NO_2.

Nitration of substrates with gaseous NO_2

```
File        :V:\GCMS\Raw_Data\57783.D
Operator    :
Acquired    : 20 Apr 2022   12:01     using AcqMethod 50T300@25C_MIN_20_SPLIT_HOLD_5MIN_EI.M
Instrument  :    GC-MSD
Sample Name: ms-jl-37-1(2022-I)
Misc Info   :
Vial Number: 4
```

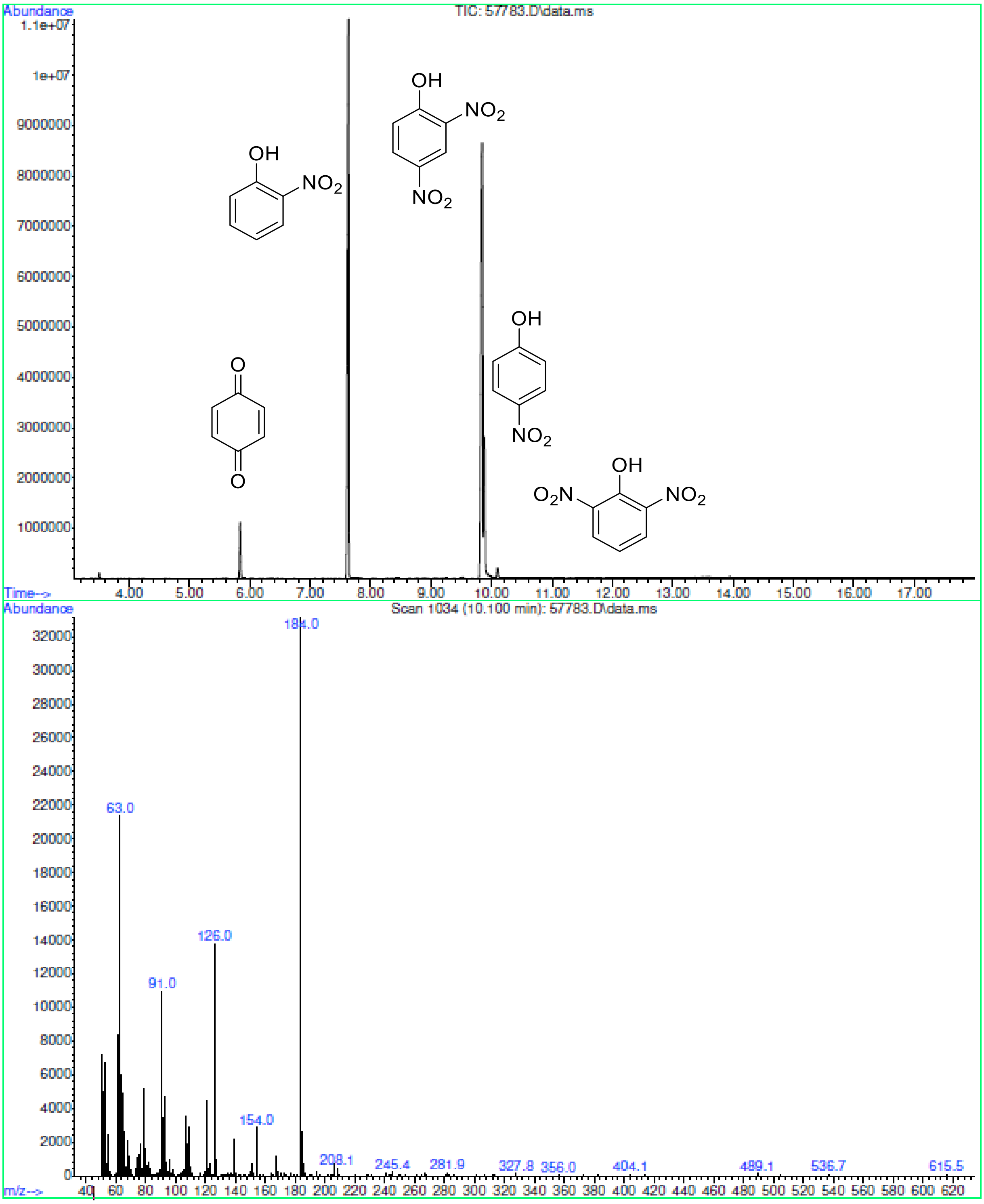

Figure S32. GC-MS plot of phenol nitration with gaseous NO_2.

```
File        :V:\GCMS\Raw_Data\57784.D
Operator    :
Acquired    : 20 Apr 2022   10:44     using AcqMethod 50T300@25C_MIN_20_SPLIT_HOLD_5MIN_EI.M
Instrument :     GC-MSD
Sample Name: ms-jl-37-2(2022-I)
Misc Info   :
Vial Number: 5
```

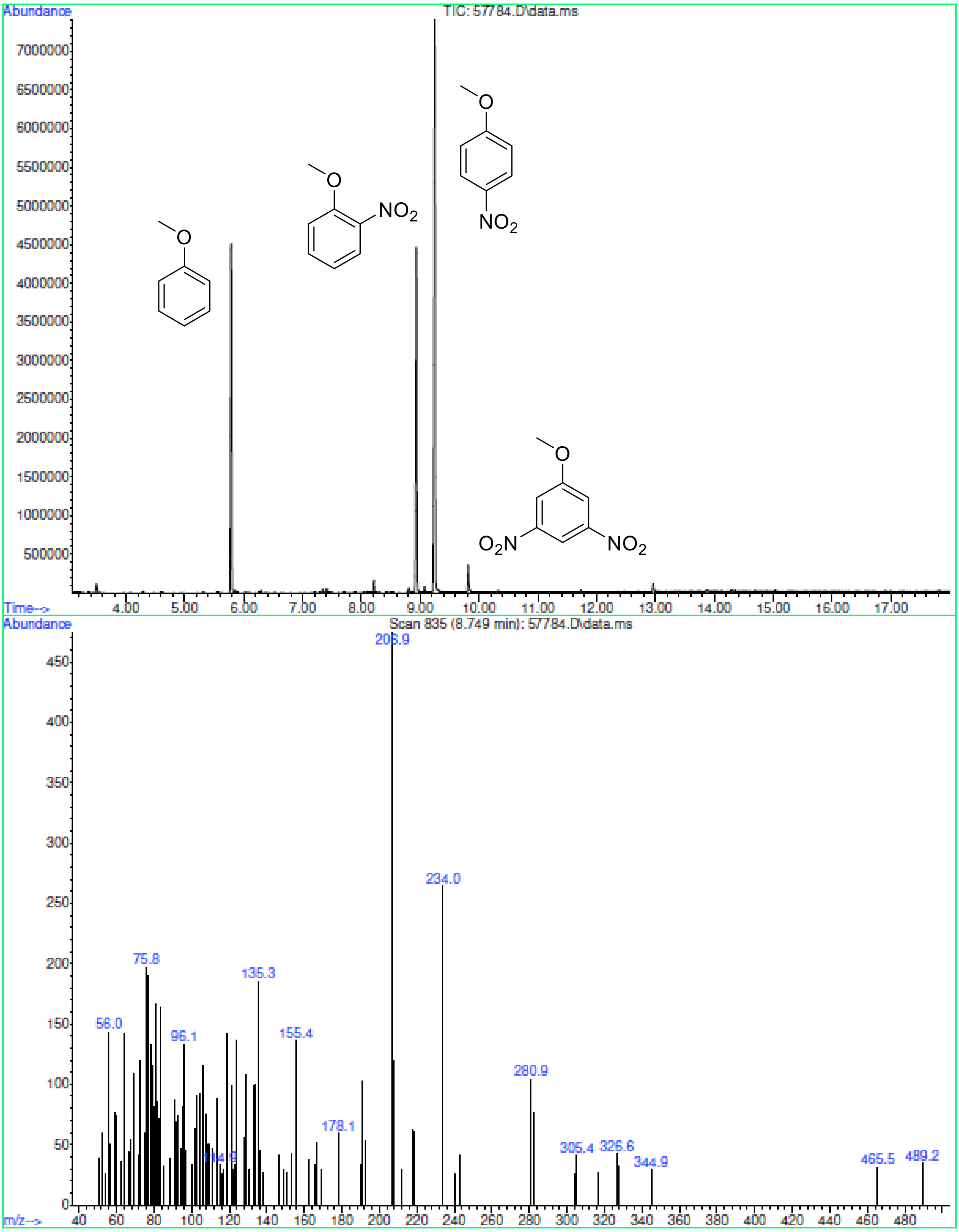

Figure S33. GC-MS plot of anisole nitration with gaseous NO_2.

```
File        :V:\GCMS\Raw_Data\57821.D
Operator    :
Acquired    : 20 Apr 2022  19:15     using AcqMethod 50T300@25C_MIN_20_SPLIT_HOLD_5MIN_EI.M
Instrument  :    GC-MSD
Sample Name: MS-JL-1-41-3 2022
Misc Info   :
Vial Number: 21
```

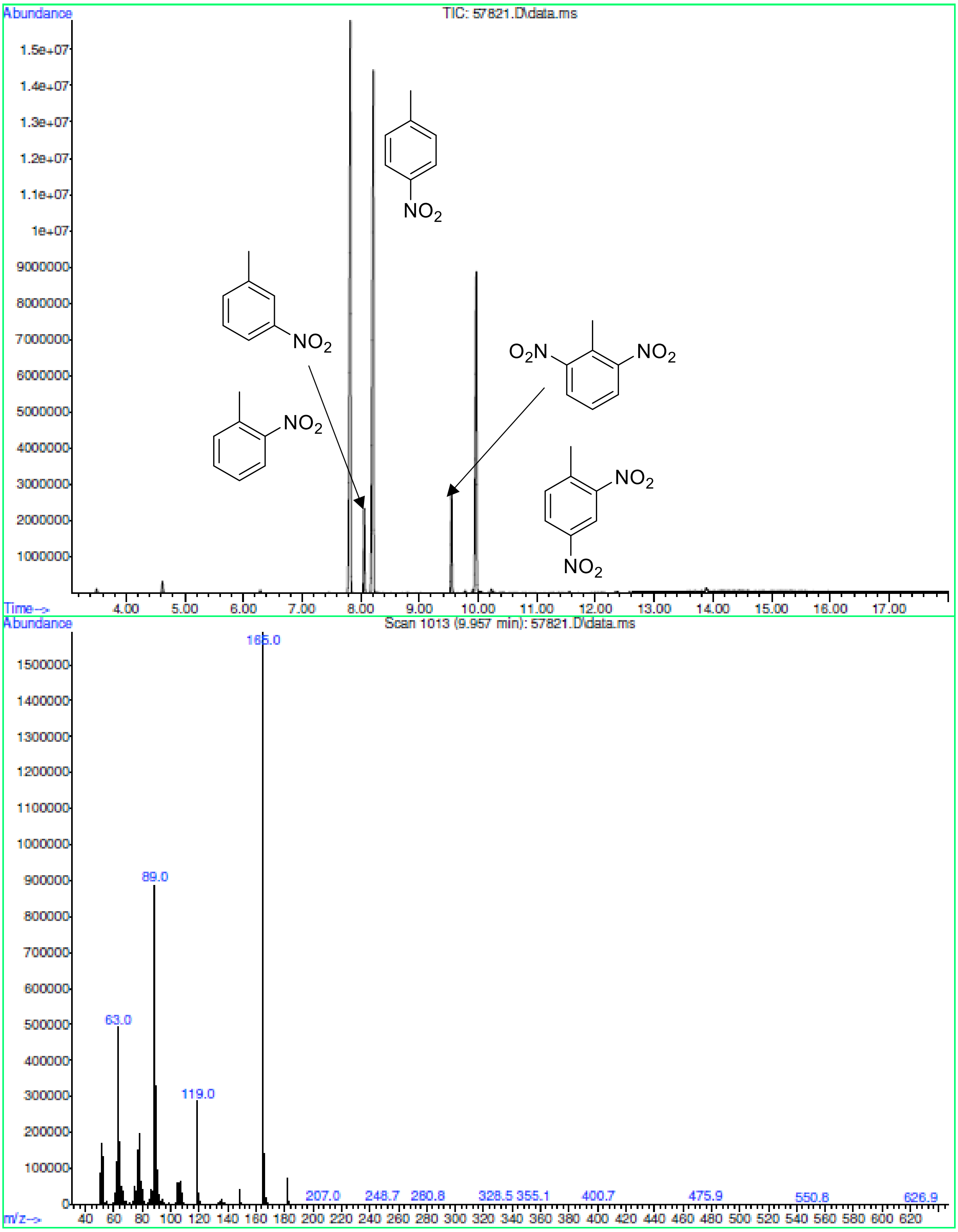

Figure S34. GC-MS plot of toluene nitration with gaseous NO_2.

```
File        :T:\58953rpt2.D
Operator    :
Acquired    : 13 May 2022   11:10      using AcqMethod 40T300@10C_MIN_20_SPLITLESS_EI.M
Instrument  :     GC-MSD
Sample Name: MS-JL-50-1(2022)
Misc Info   :
Vial Number: 53
```

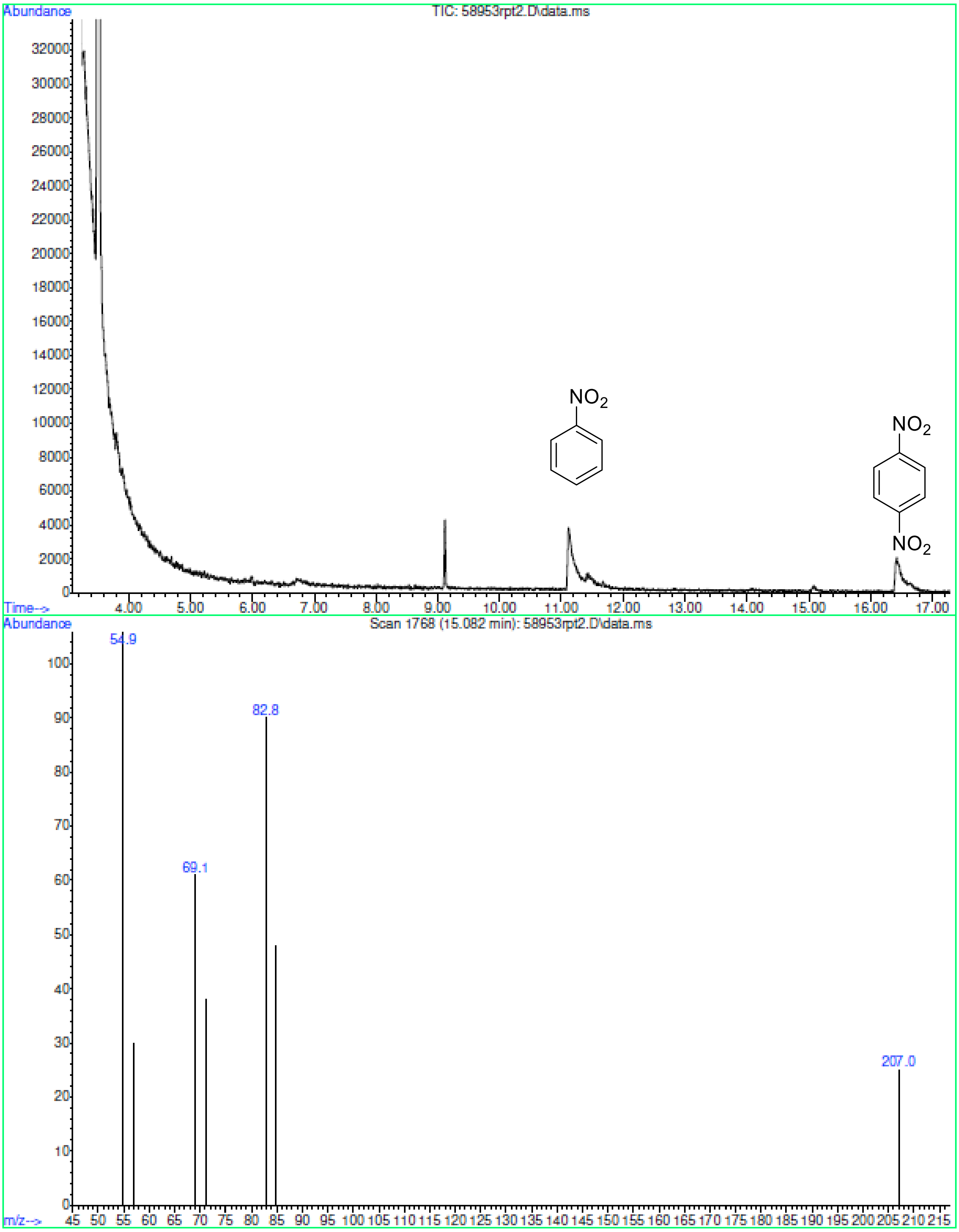

Figure S35. GC-MS plot of benzene nitration with gaseous NO_2.

```
File        :V:\GCMS\Raw_Data\57948.D
Operator    :
Acquired    : 25 Apr 2022  17:22     using AcqMethod 50T300@25C_MIN_20_SPLIT_HOLD_5MIN_EI.M
Instrument :     GC-MSD
Sample Name: MS-JL-42-4(2022)
Misc Info   :
Vial Number: 48
```

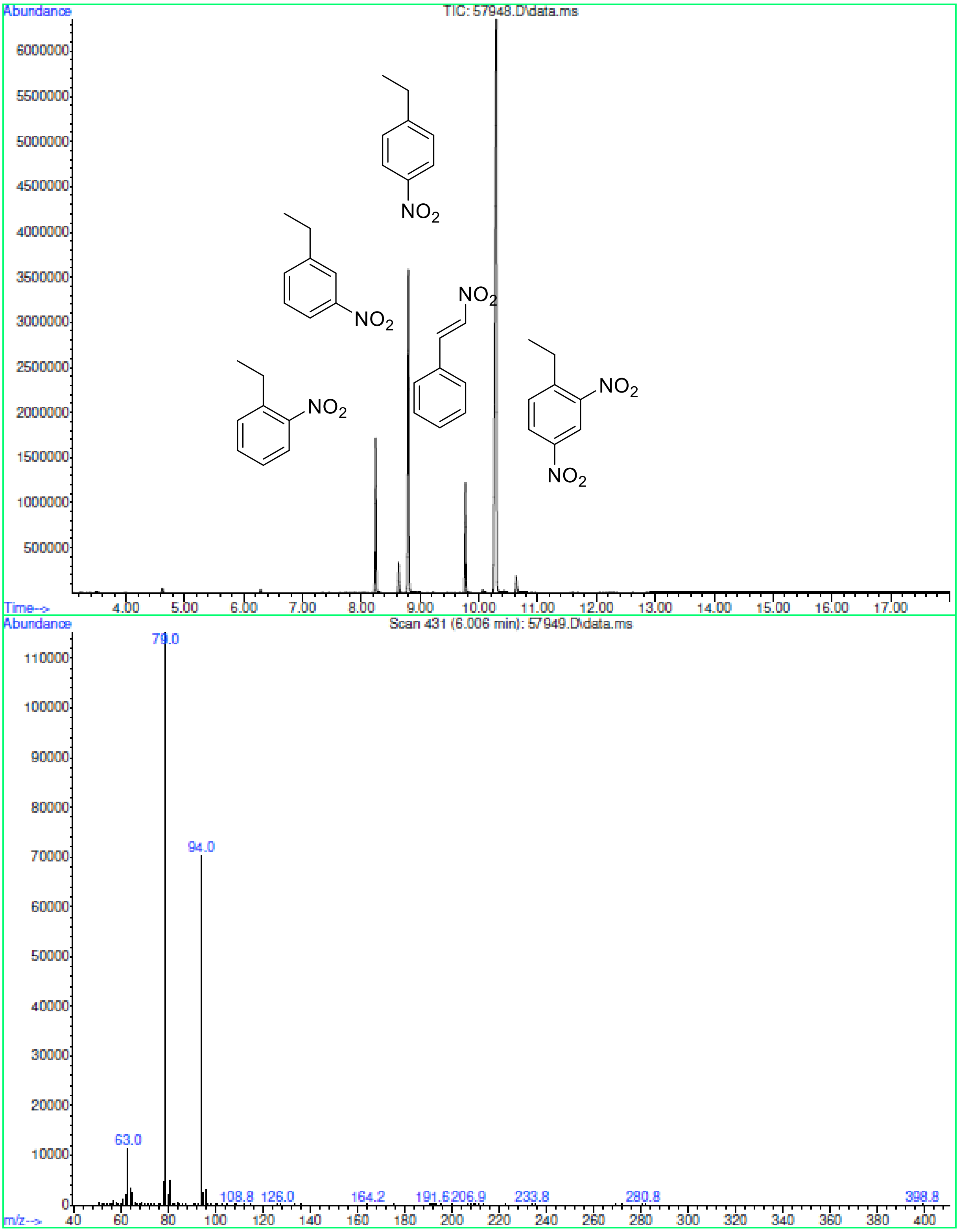

Figure S36. GC-MS plot of ethylbenzene nitration with gaseous NO_2.

Nitration of substrates with NO_2@Zr-bptc-N

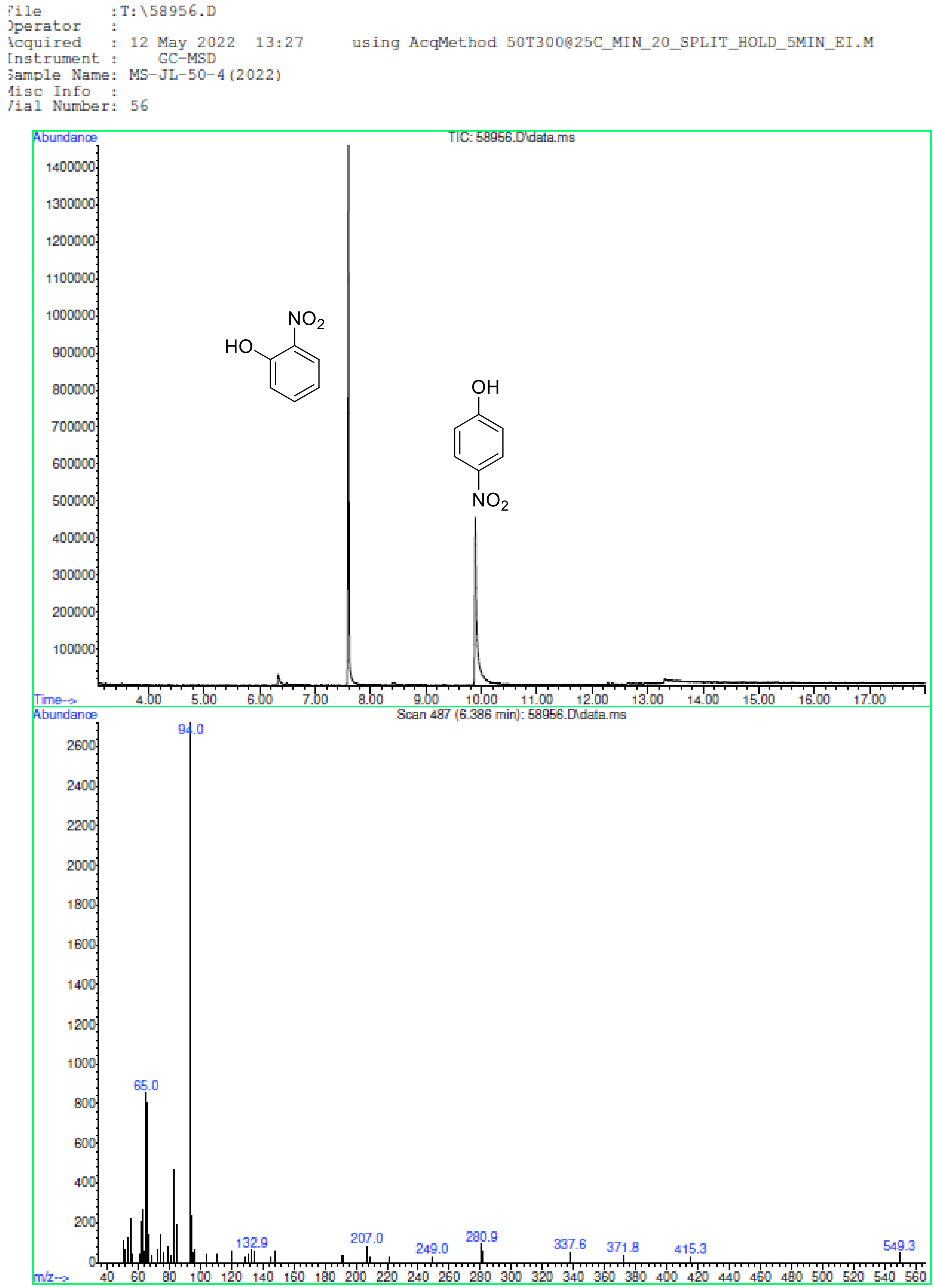

Figure S37. GC-MS plot of phenol nitration with NO_2@Zr-bptc-N.

```
File        :T:\58955.D
Operator    :
Acquired    : 12 May 2022  14:01     using AcqMethod 50T300@25C_MIN_20_SPLIT_HOLD_5MIN_EI.M
Instrument :     GC-MSD
Sample Name: MS-JL-50-2(2022)
Misc Info   :
Vial Number: 55
```

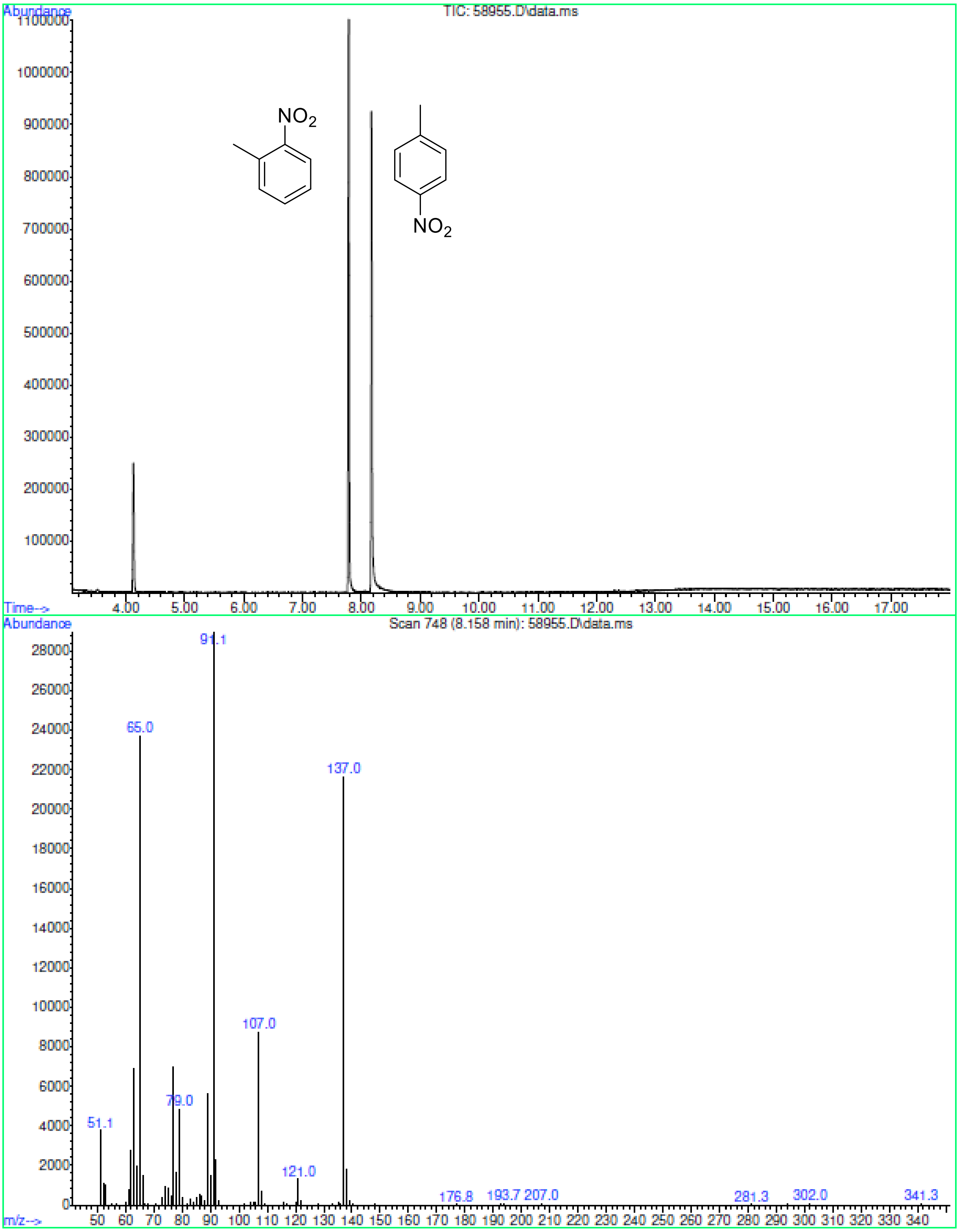

Figure S38. GC-MS plot of toluene nitration with NO_2@Zr-bptc

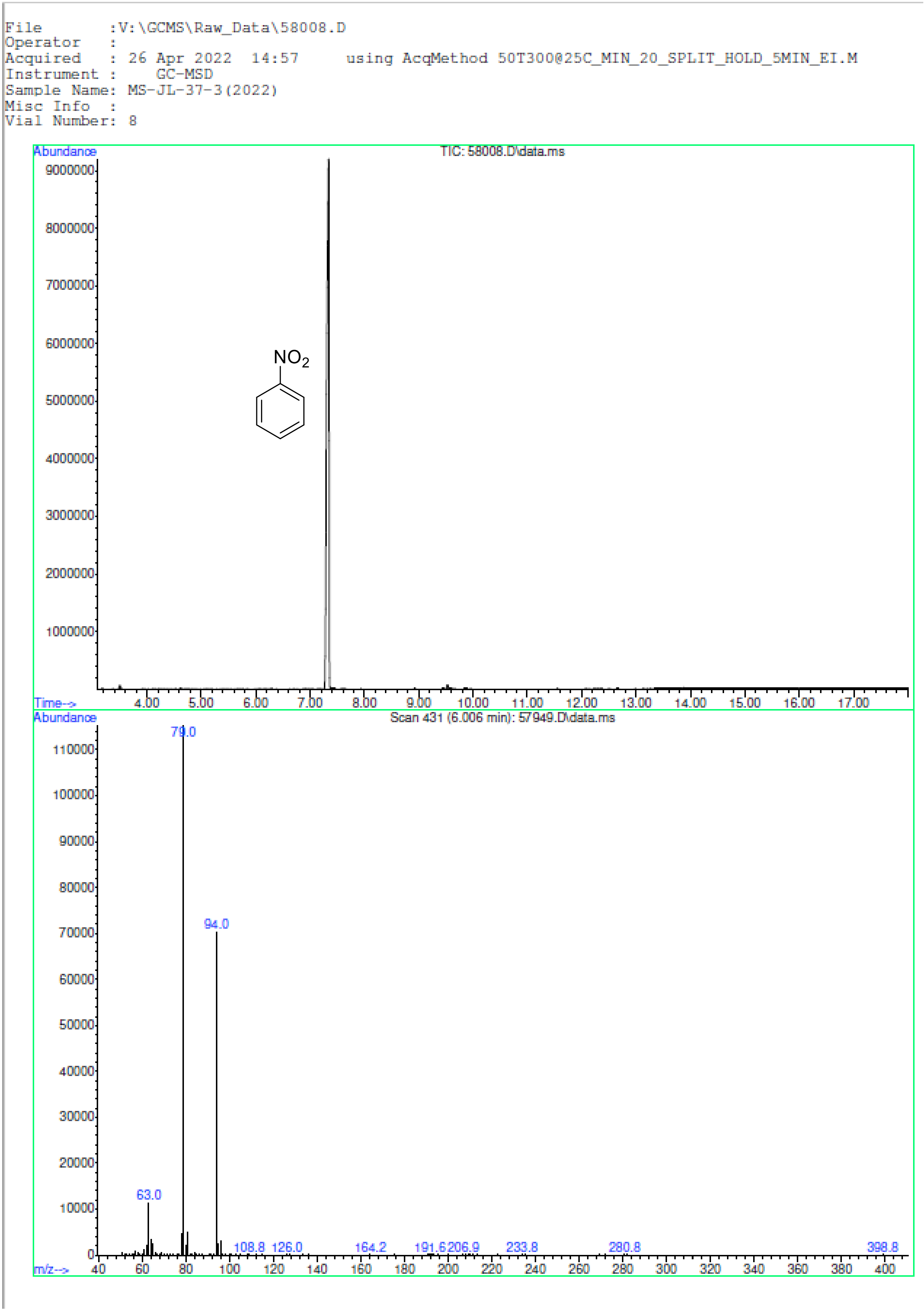

Figure S39. GC-MS plot of nitration of benzene with NO_2@Zr-bptc-N.

```
File       :T:\58954.D
Operator   :
Acquired   : 12 May 2022   14:36     using AcqMethod 50T300@25C_MIN_20_SPLIT_HOLD_5MIN_EI.M
Instrument :     GC-MSD
Sample Name: MS-JL-50-3(2022)
Misc Info  :
Vial Number: 54
```

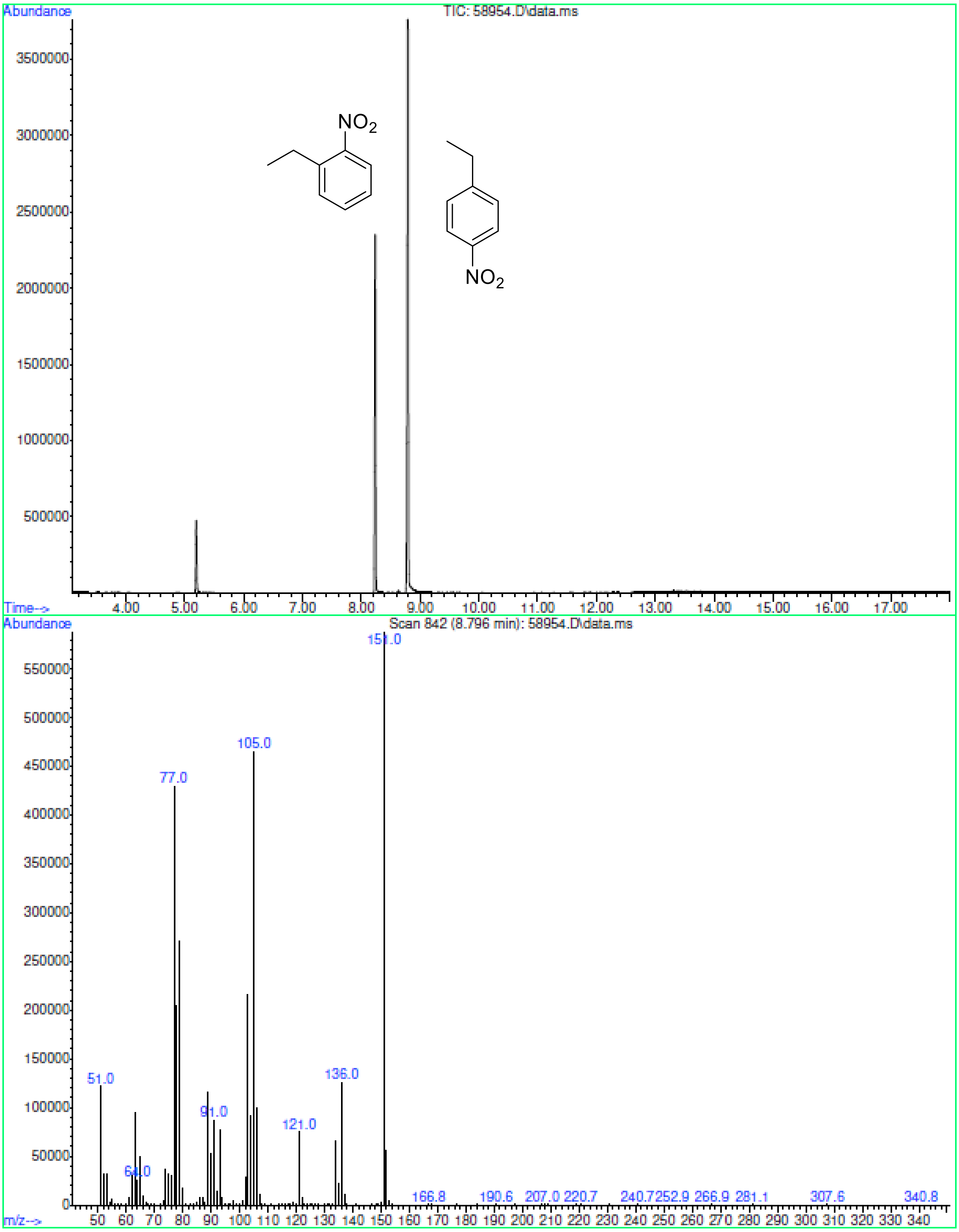

Figure S40. GC-MS plot of nitration anisole with NO_2@Zr-bptc-N.

NMR spectra of nitration products using NO_2@Zr-bptc-N

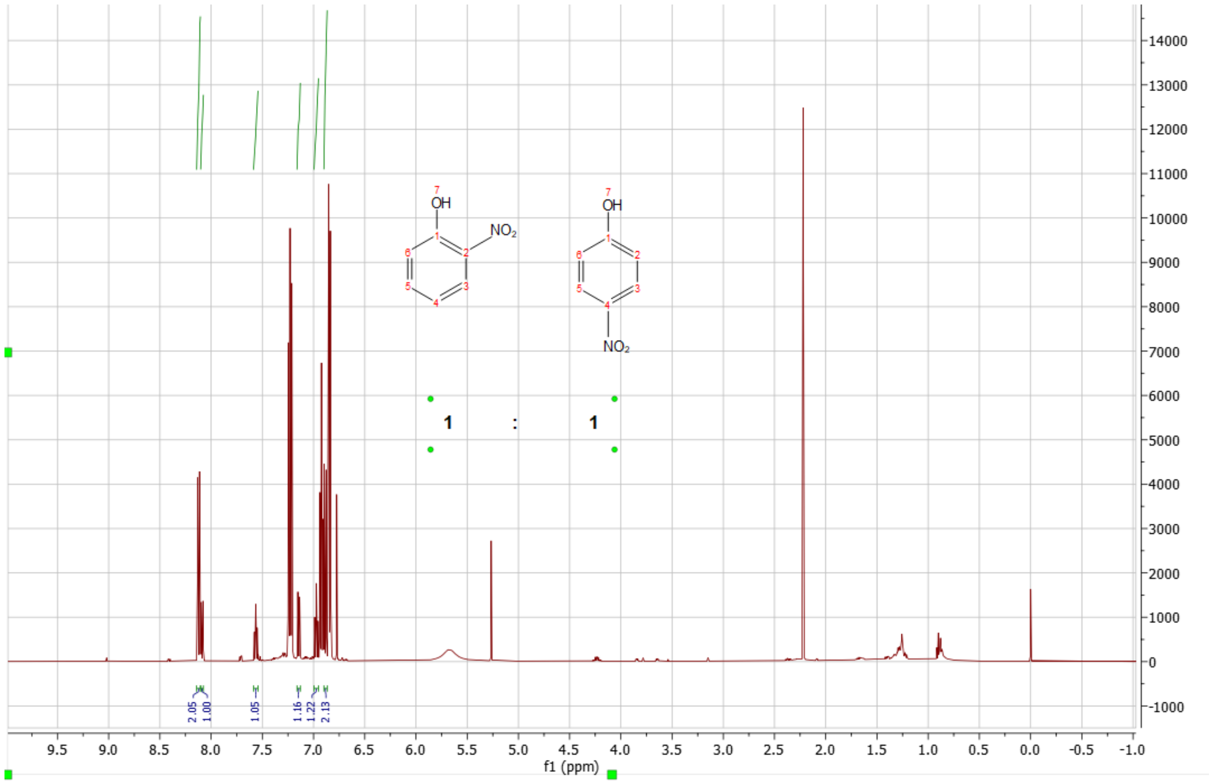

Figure S41. ^{1}H NMR spectrum of reaction mixture with phenol.

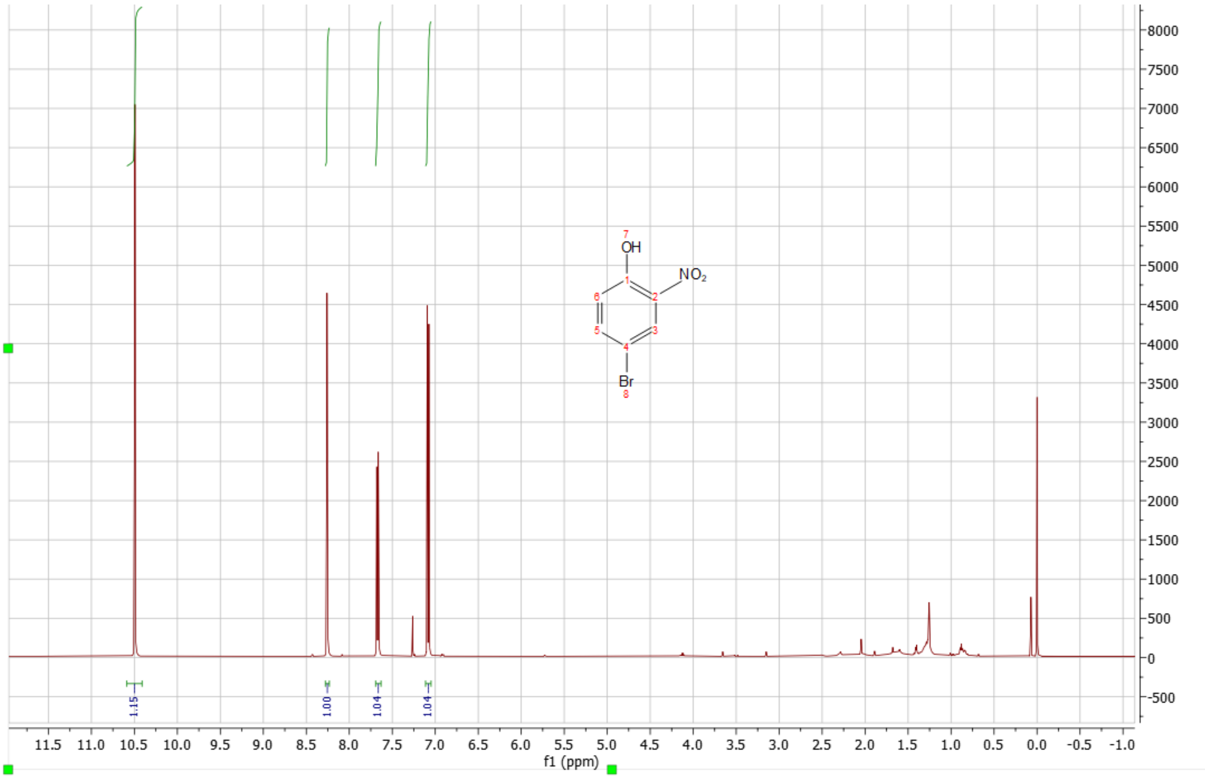

Figure S42. ^{1}H NMR spectrum of reaction mixture with 4-bromophenol.

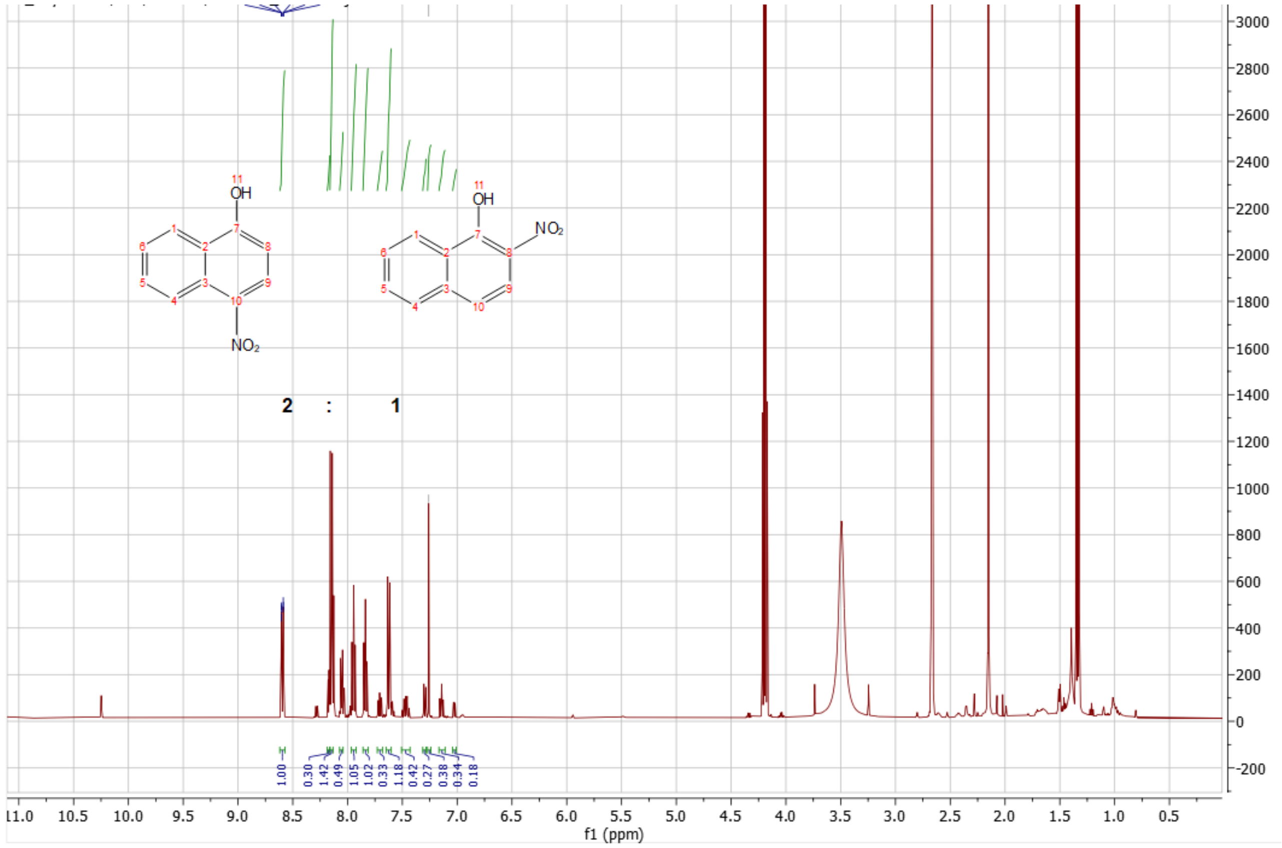

Figure S43. ^{1}H NMR spectrum of reaction mixture with naphthalen-1-ol.

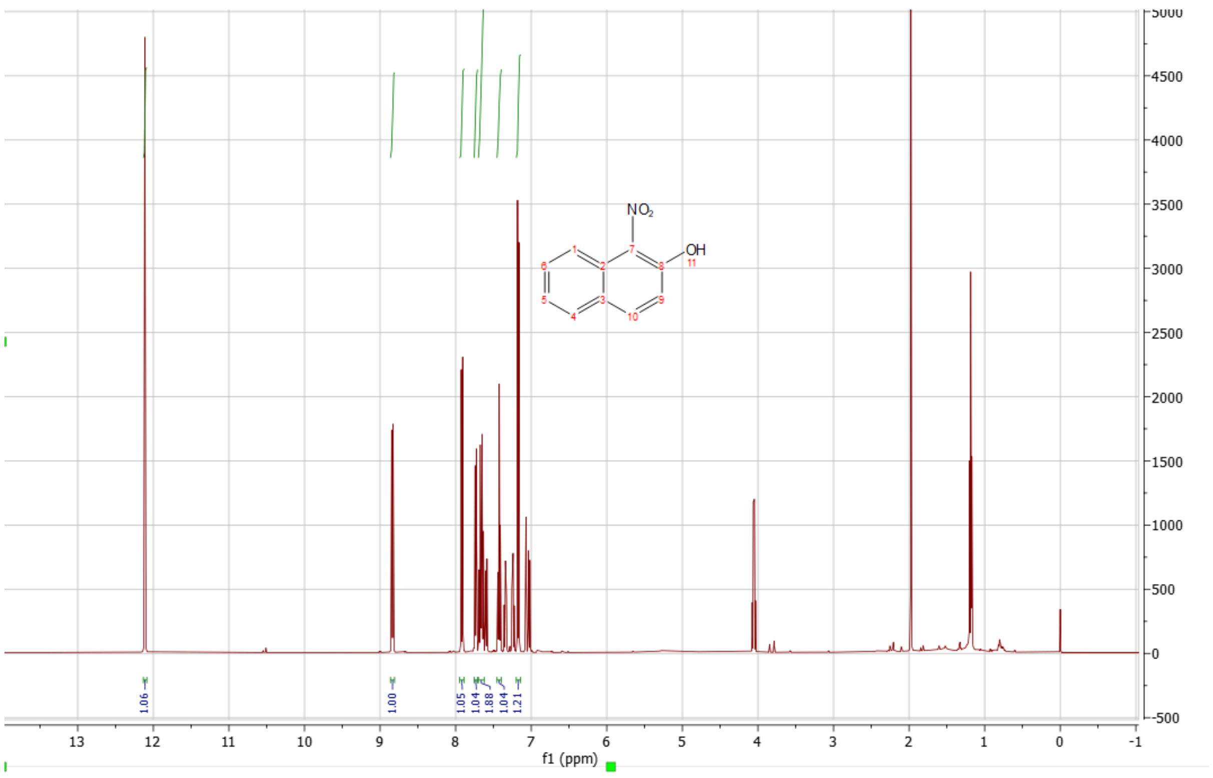

Figure S44. ^{1}H NMR spectrum of reaction mixture with naphthalen-2-ol.

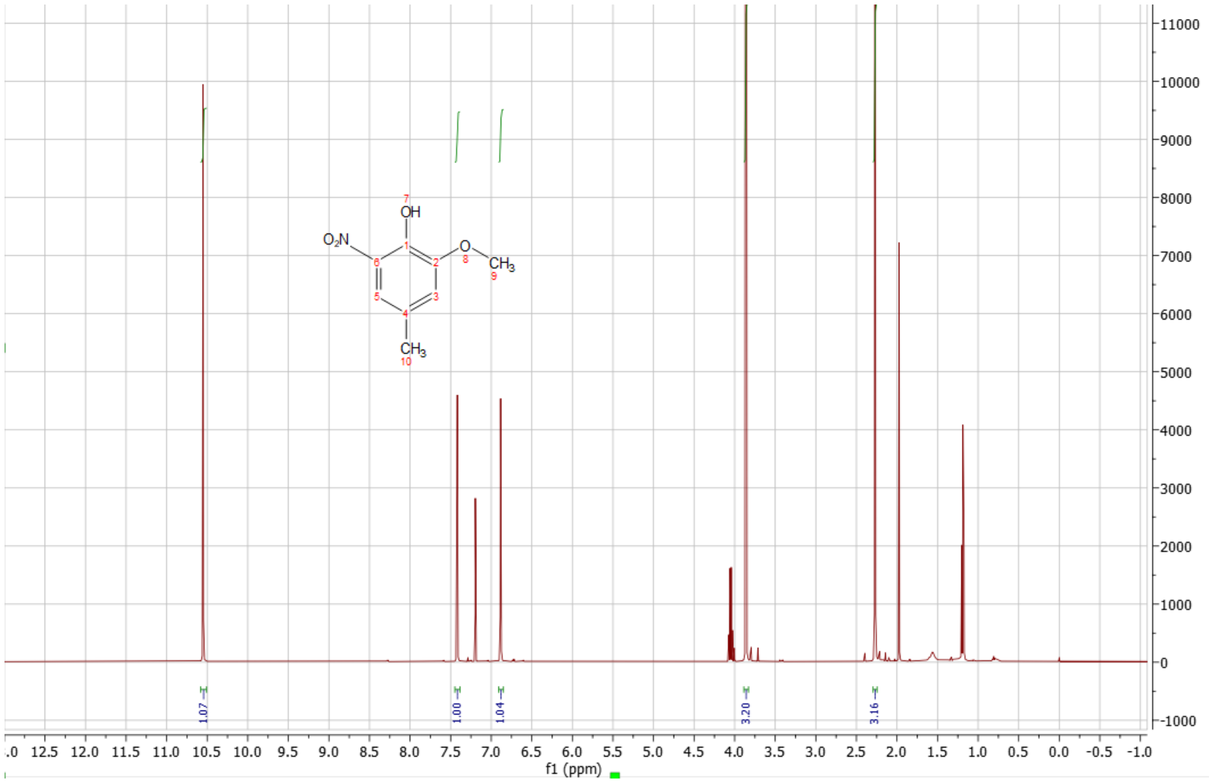

Figure S45. ^{1}H NMR spectrum of reaction mixture with 2-methoxy-4-methyl-phenol.

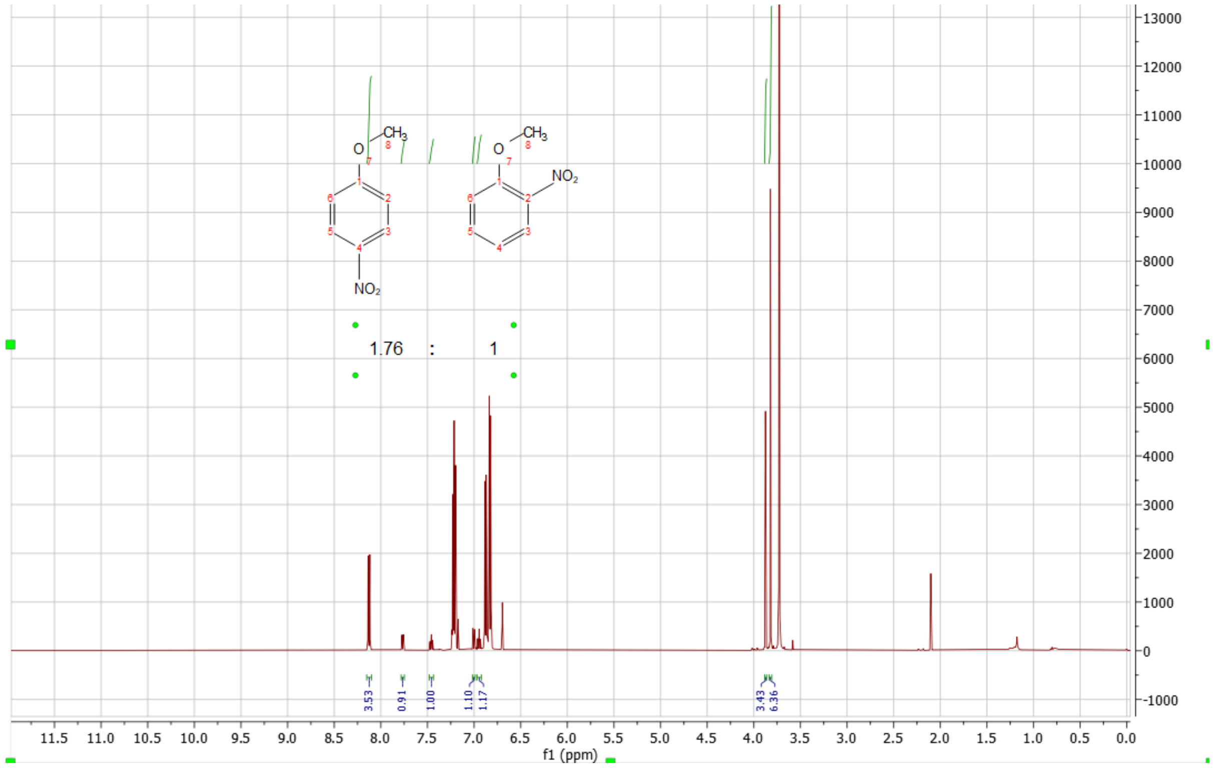

Figure S46. ^{1}H NMR spectrum of reaction mixture with anisole.

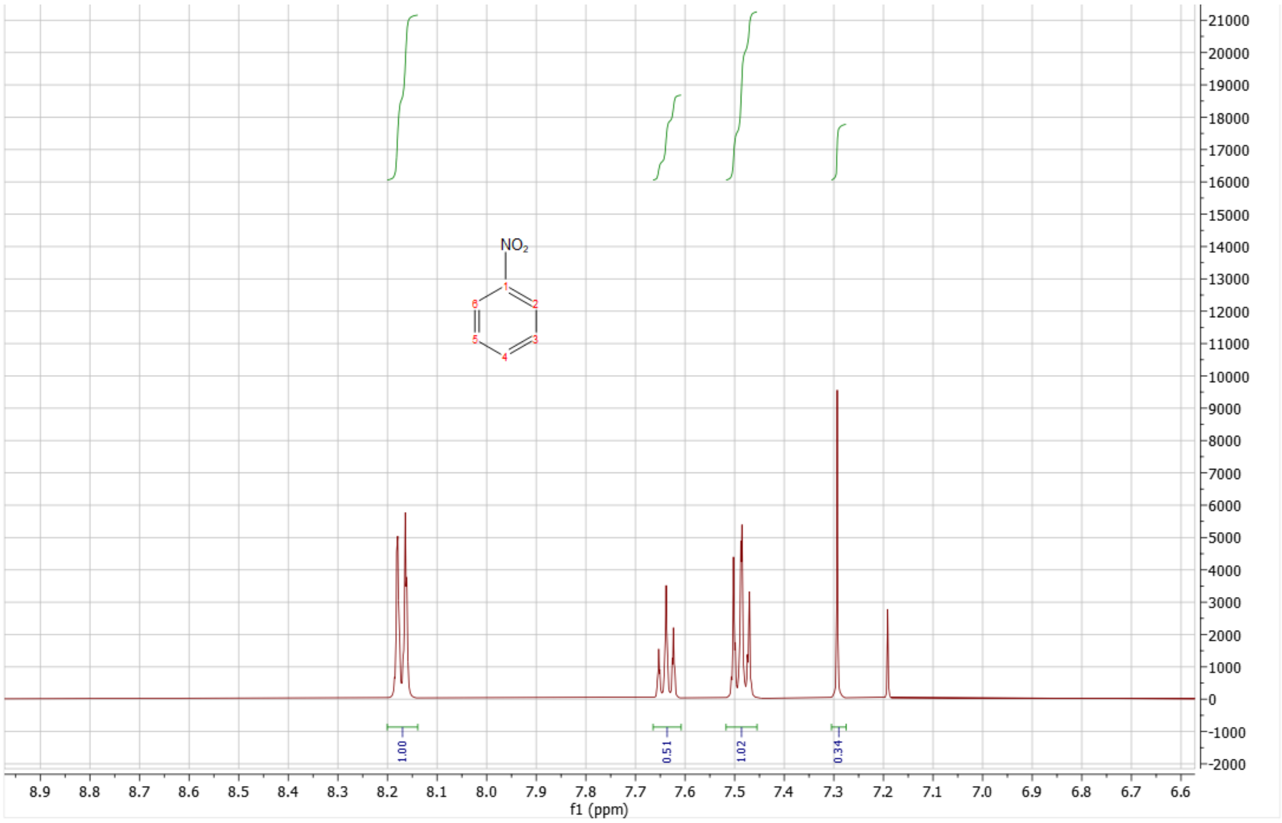

Figure S47. ^{1}H NMR spectrum of reaction mixture with benzene.

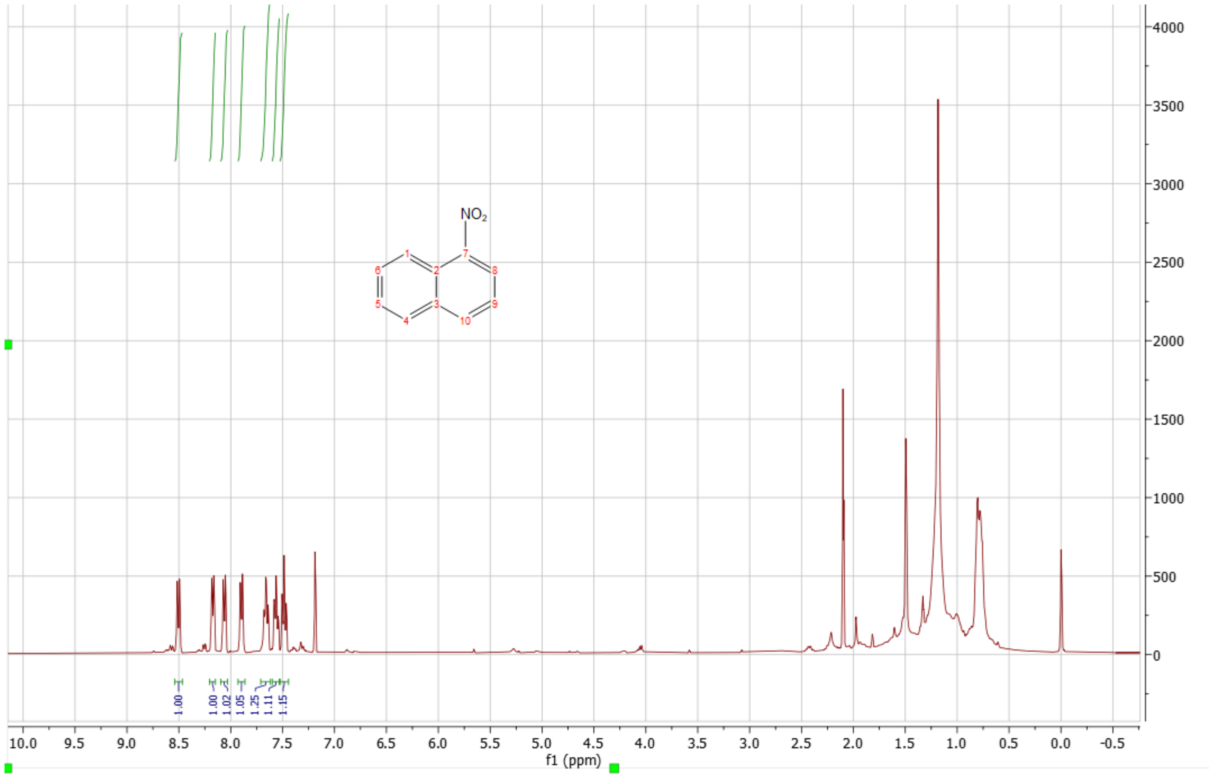

Figure S48. ^{1}H NMR spectrum of reaction mixture with naphthalene.

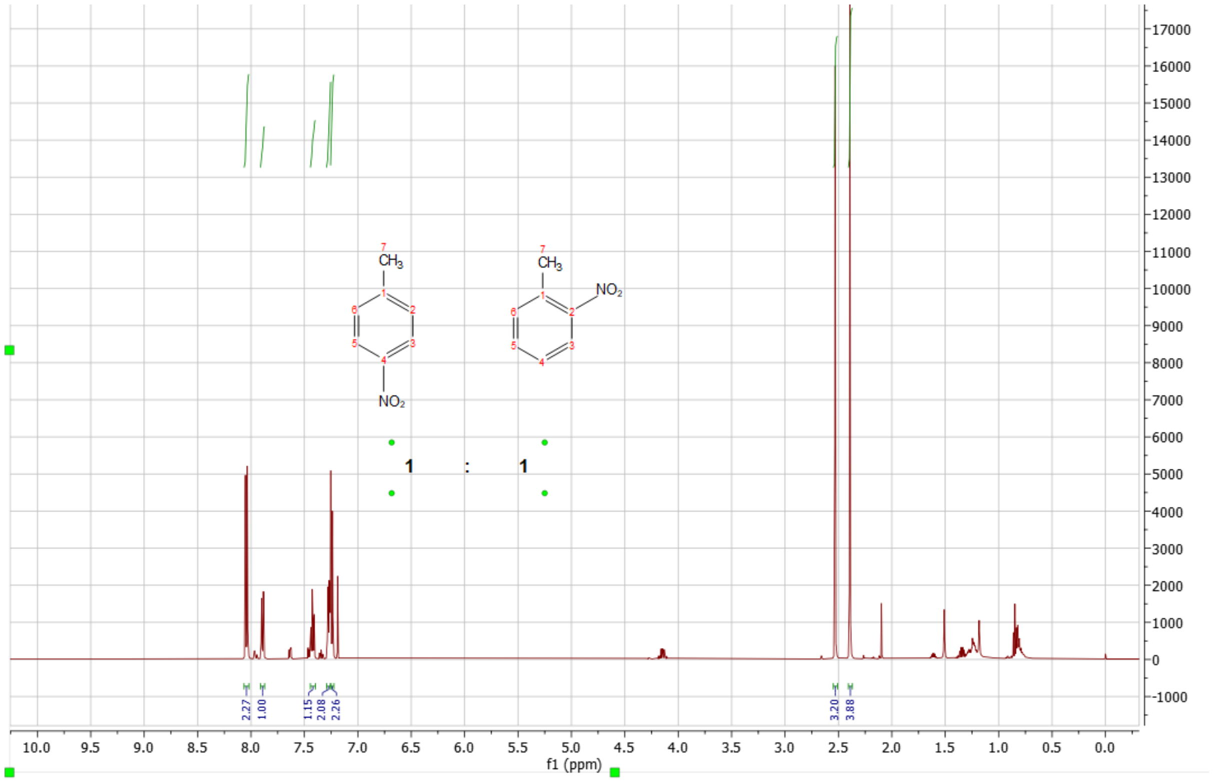

Figure S49. ^{1}H NMR spectrum of reaction mixture with toluene.

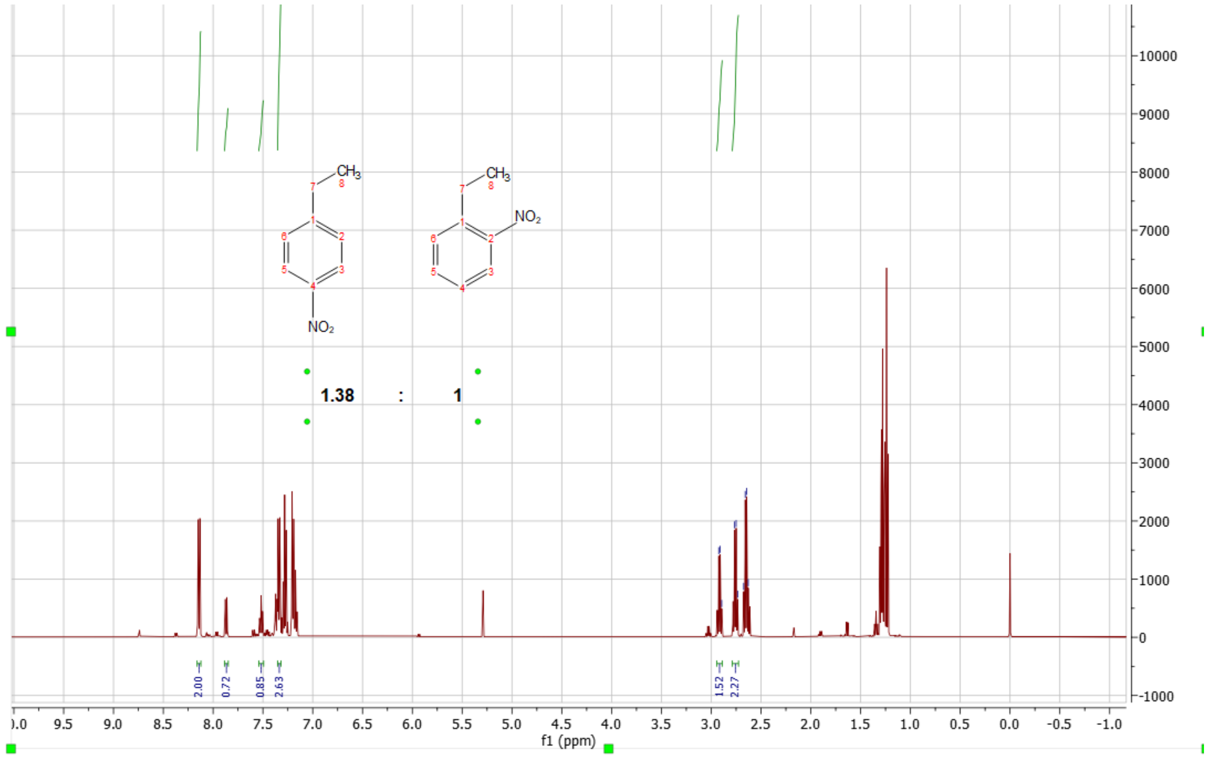

Figure S50. ^{1}H NMR spectrum of reaction mixture with ethylbenzene.

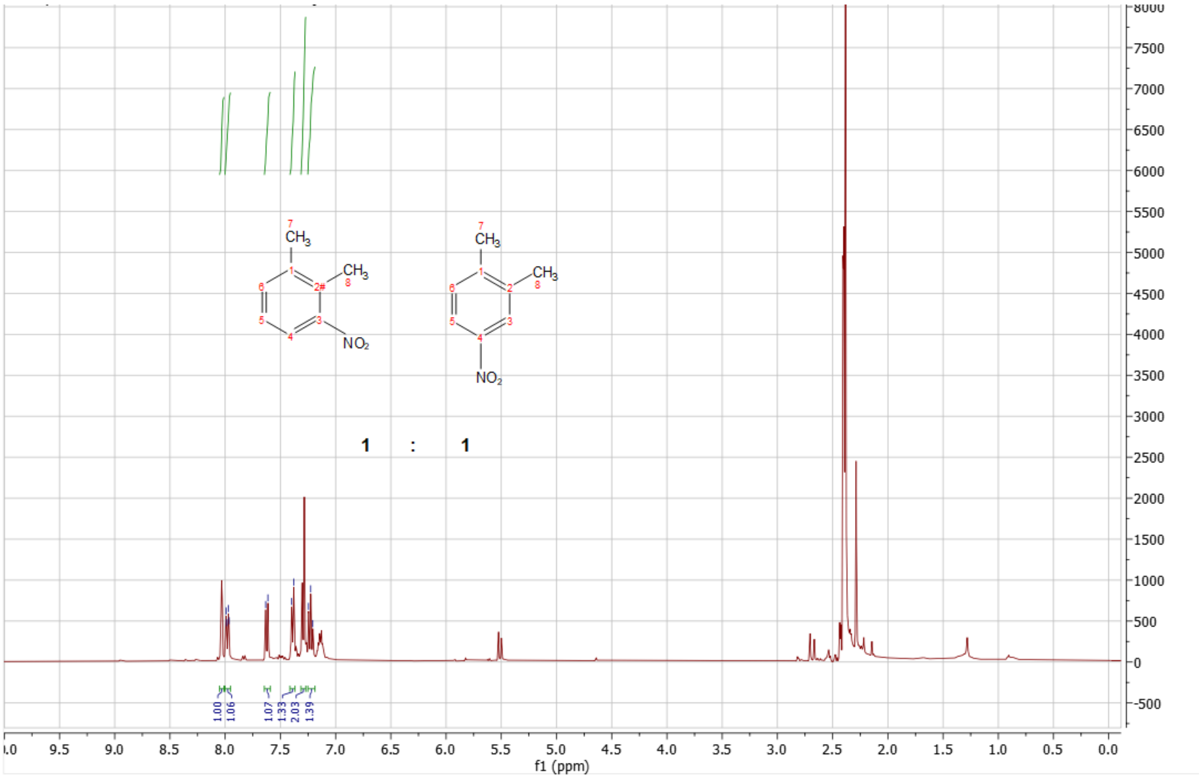

Figure S51. ^{1}H NMR spectrum of reaction mixture with *o*-xylene.

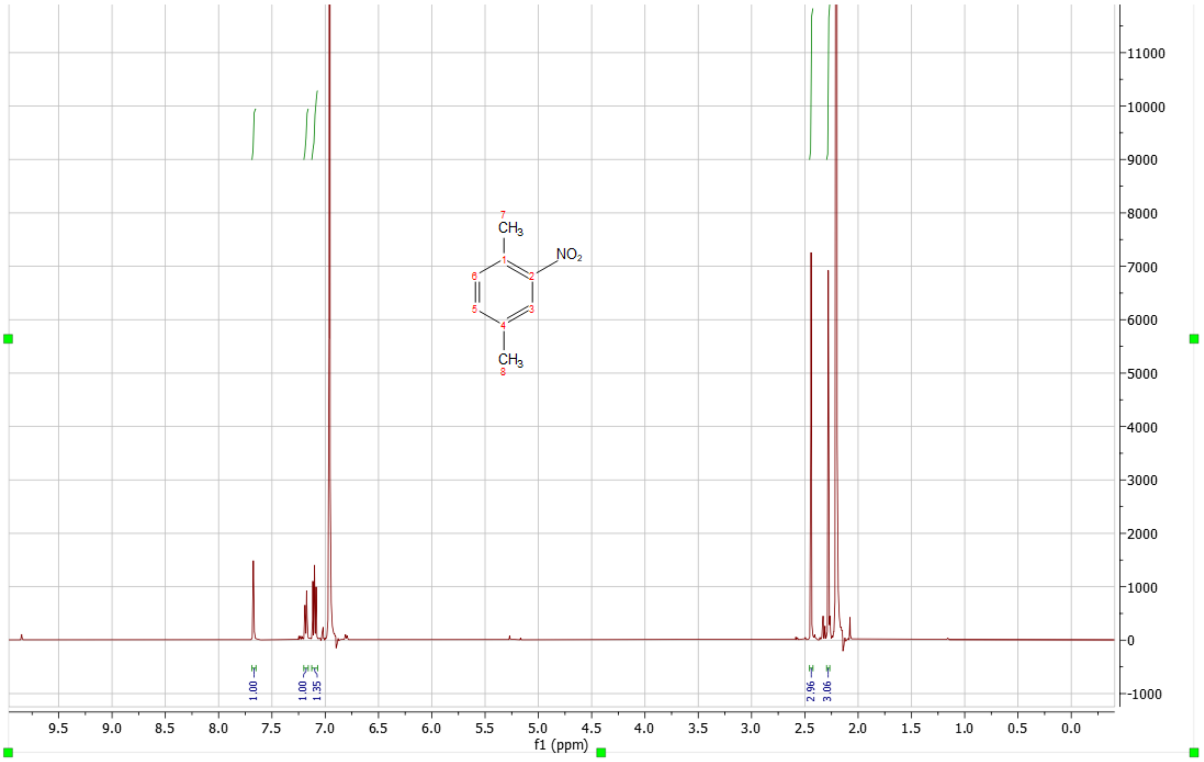

Figure S52. ^{1}H NMR spectrum of reaction mixture with *p*-xylene.

NMR spectra of purified nitration products

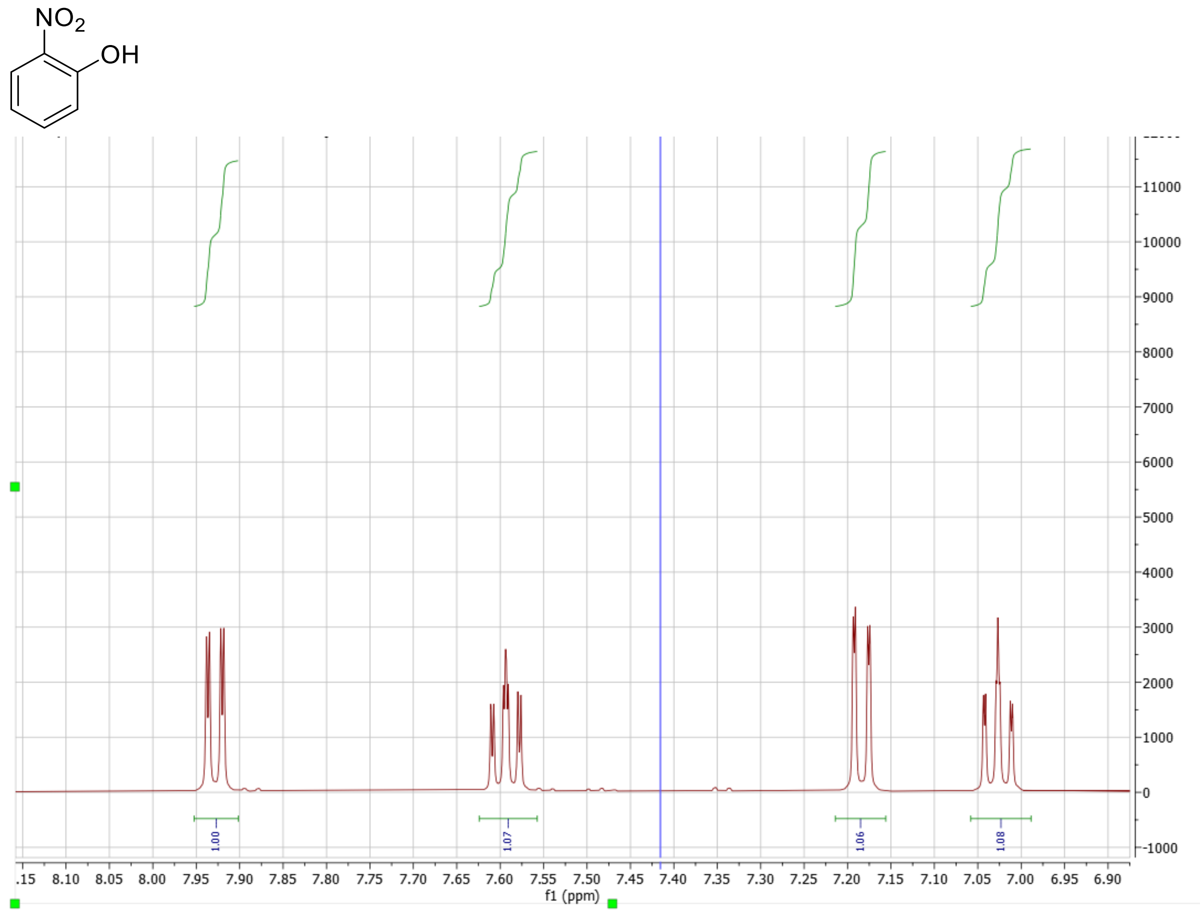

Figure S53. ^{1}H NMR spectrum of 2-nitrophenol.

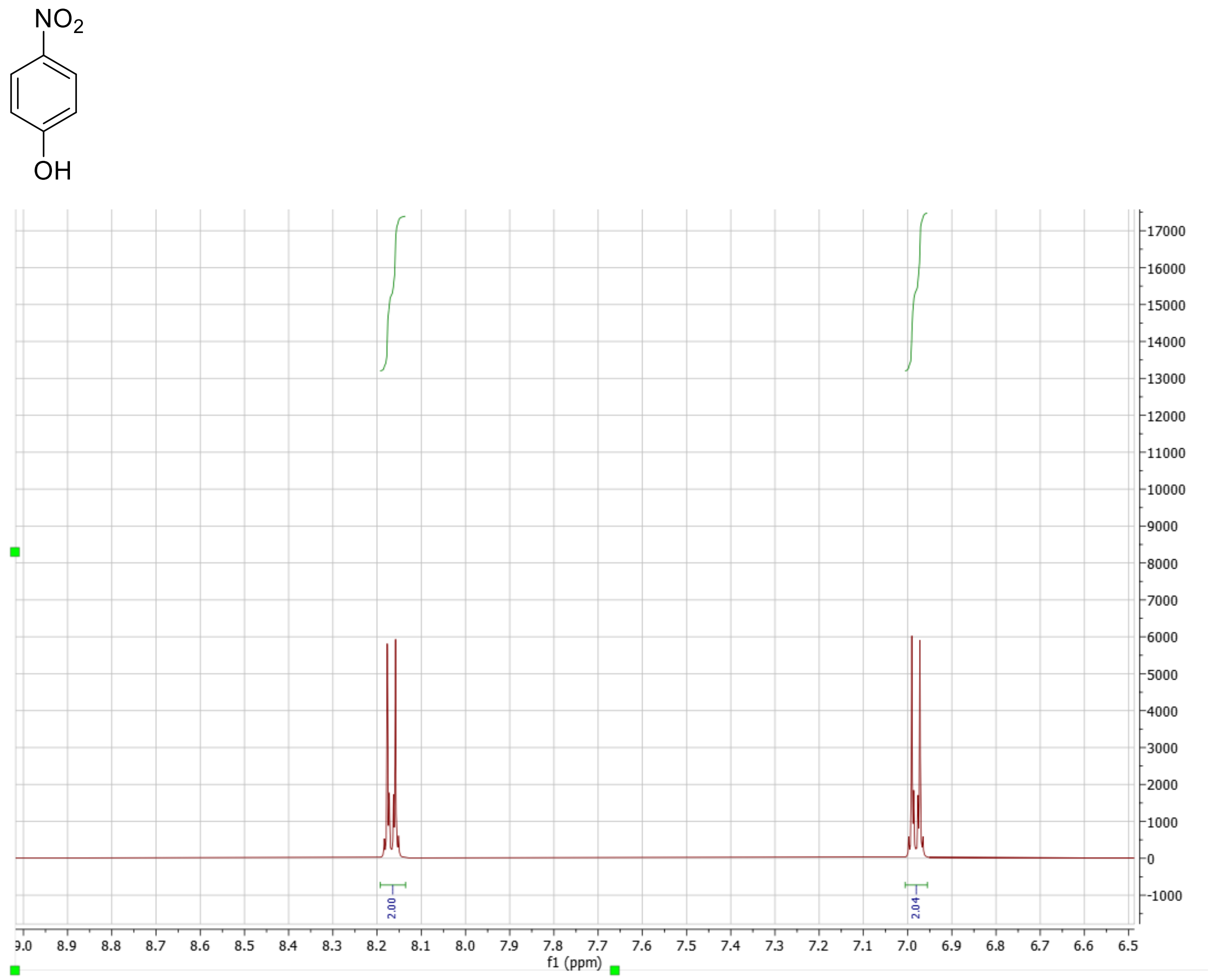

Figure S54. ^{1}H NMR spectrum of 4-nitrophenol.

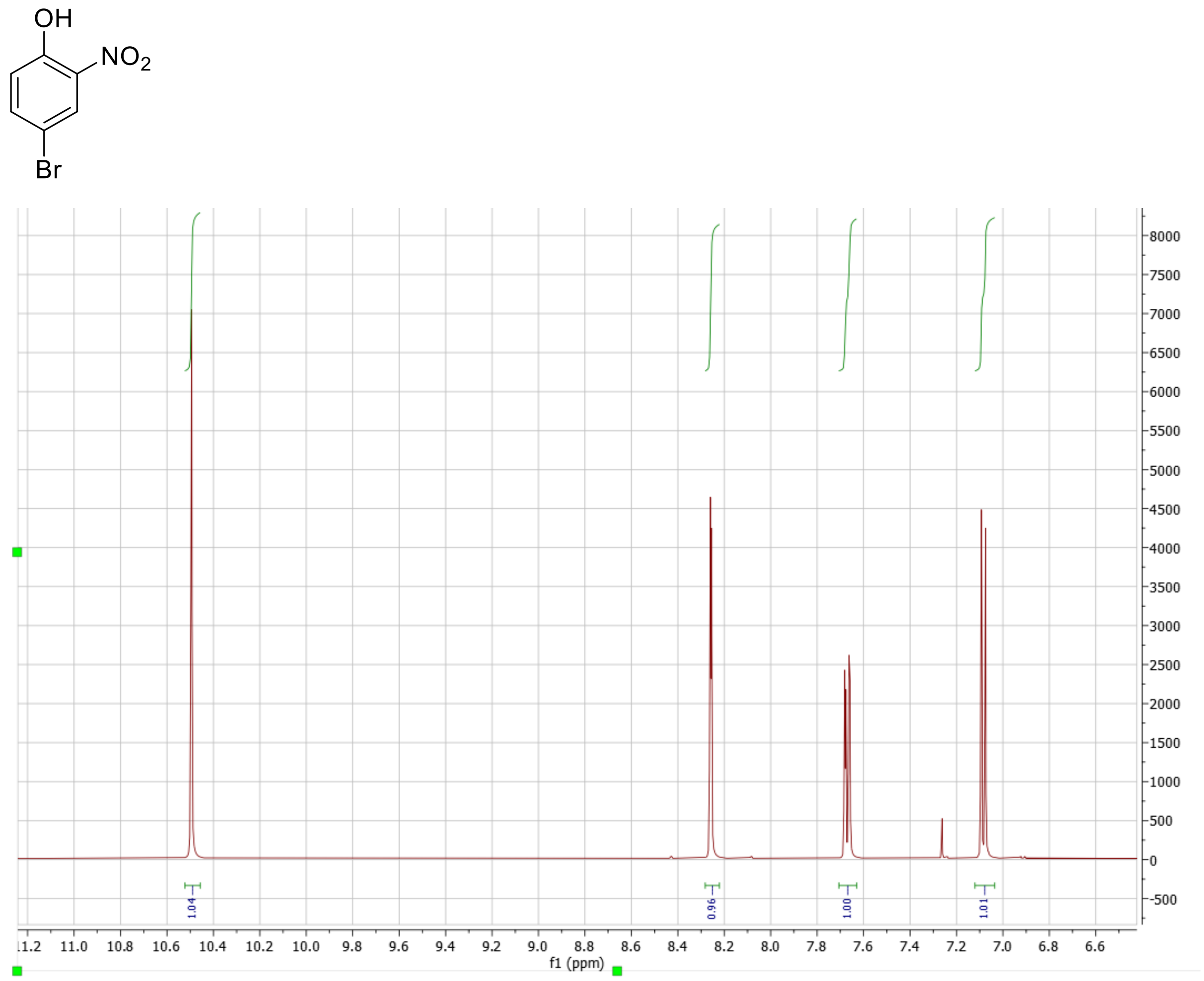

Figure S55. ^{1}H NMR spectrum of 4-bromo-2-nitrophenol.

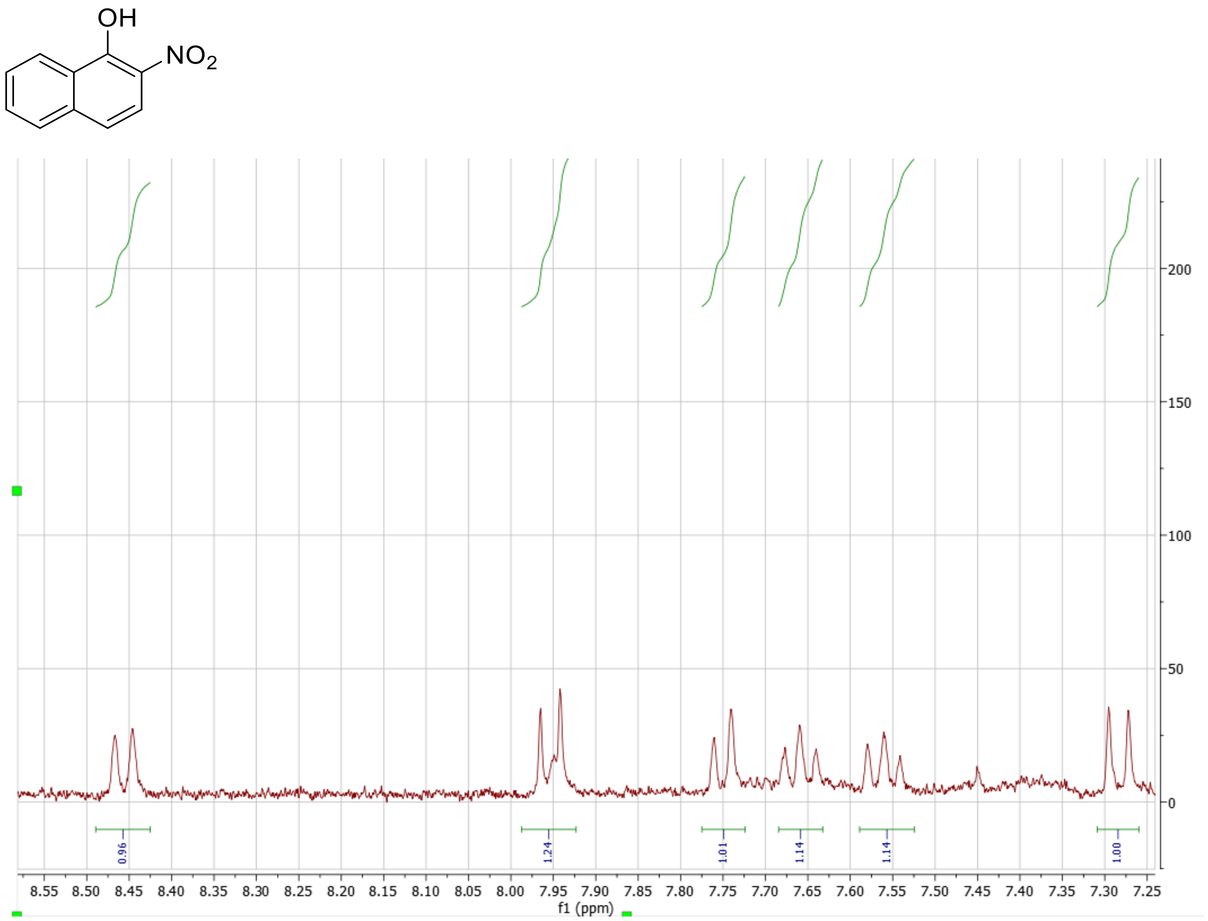

Figure S56. ^{1}H NMR spectrum of 2-nitronaphthalen-1-ol.

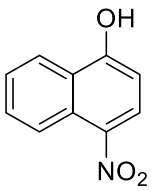

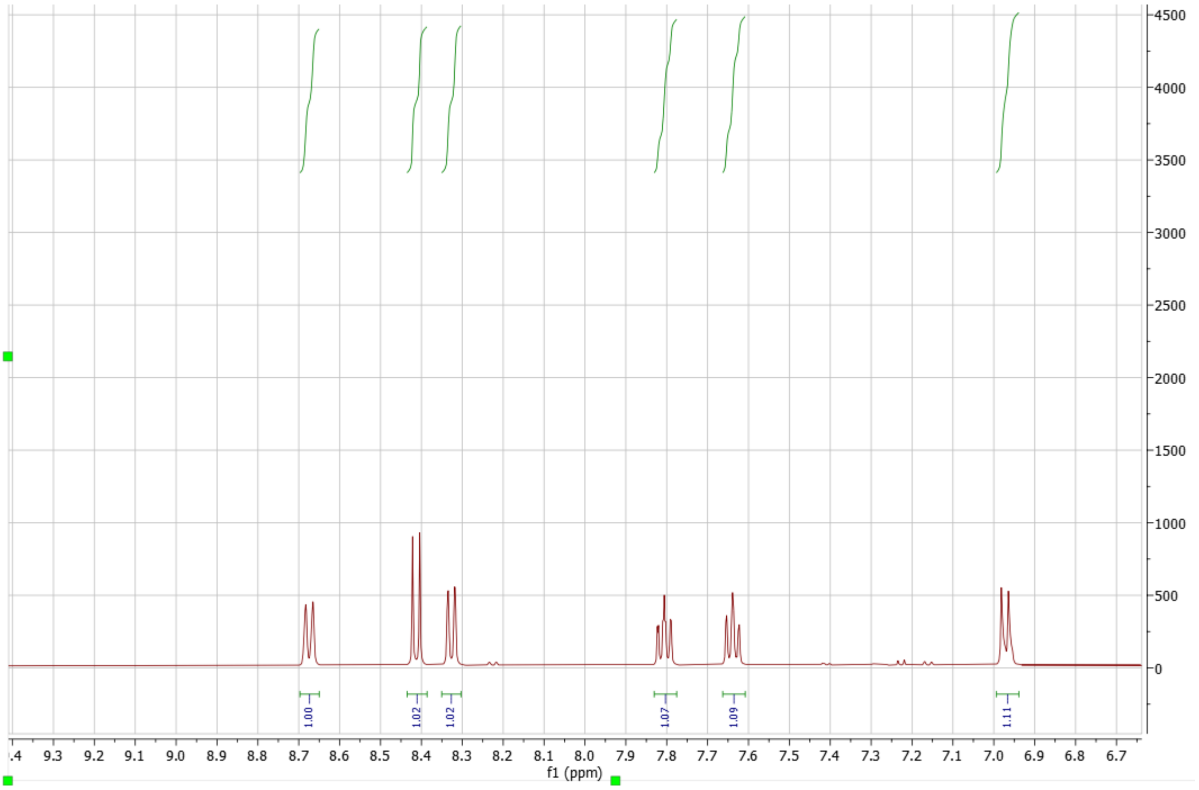

Figure S57. ^{1}H NMR spectrum of 4-nitronaphthalen-1-ol.

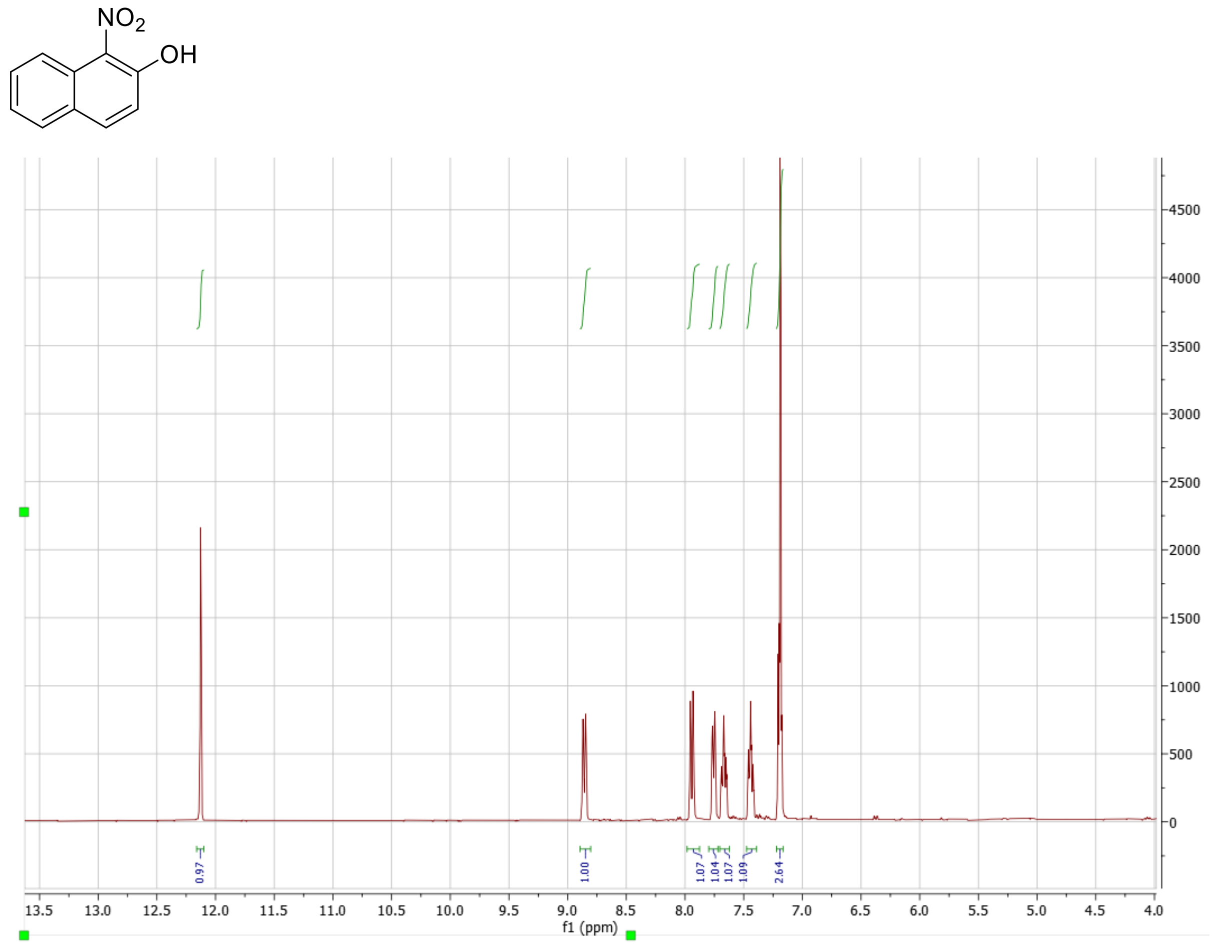

Figure S58. ^{1}H NMR spectrum of 1-nitronaphthalen-2-ol.

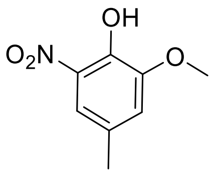

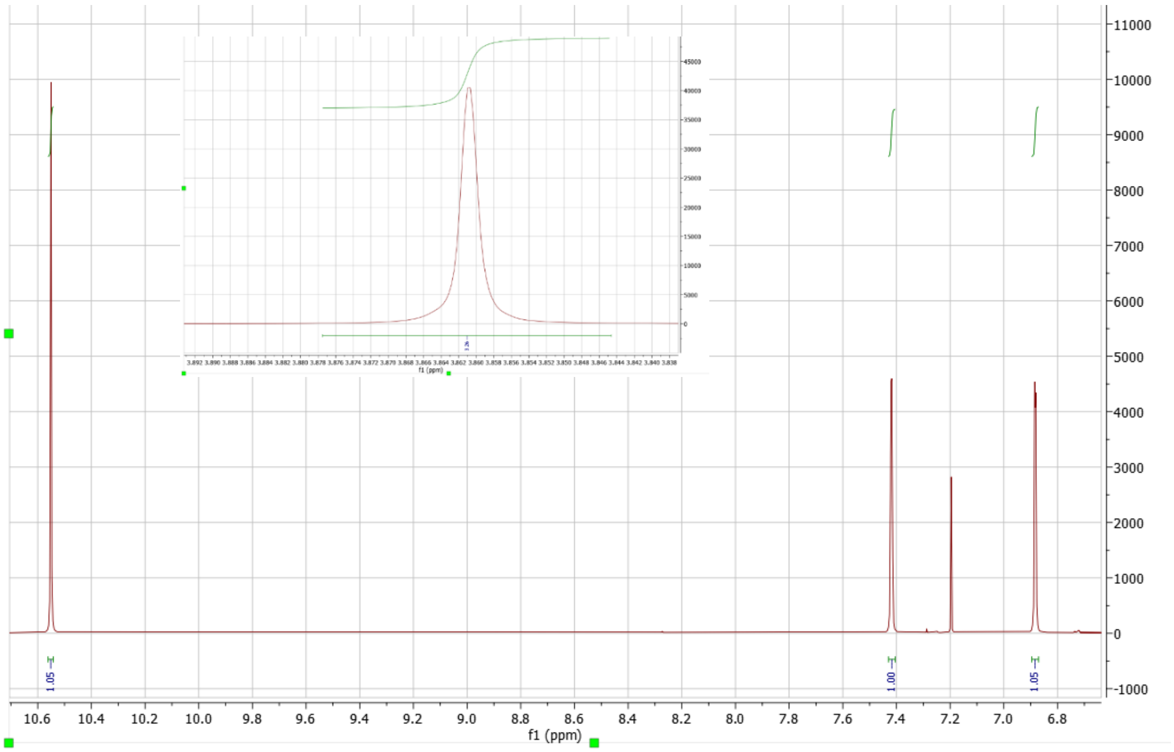

Figure S59. ^{1}H NMR spectrum of 2-methoxy-4-methyl-6-nitrophenol (inset: high field peak).

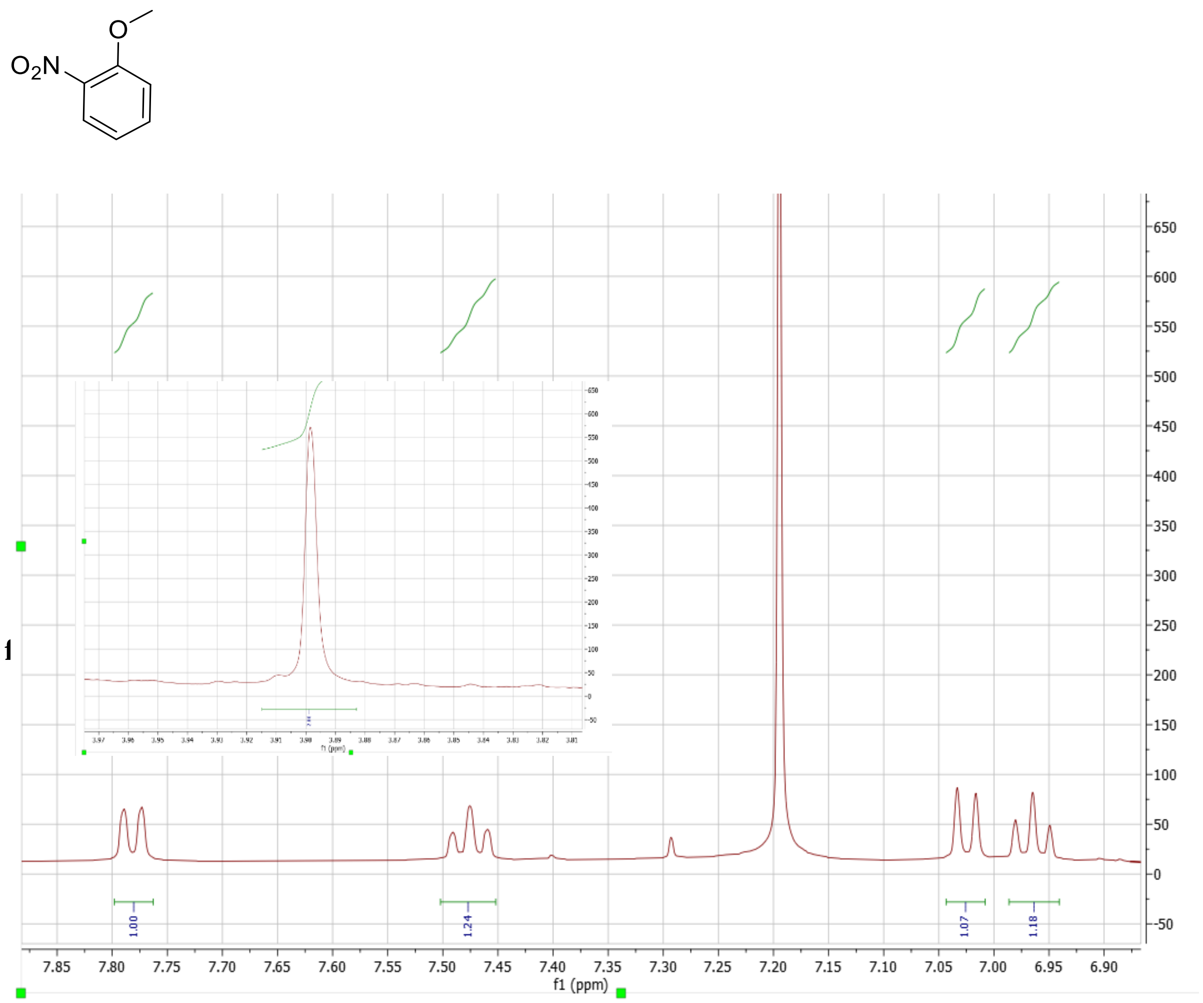

Figure S60. ¹H NMR spectrum of 1-methoxy-2-nitrobenzene (inset: high field peak).

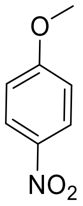

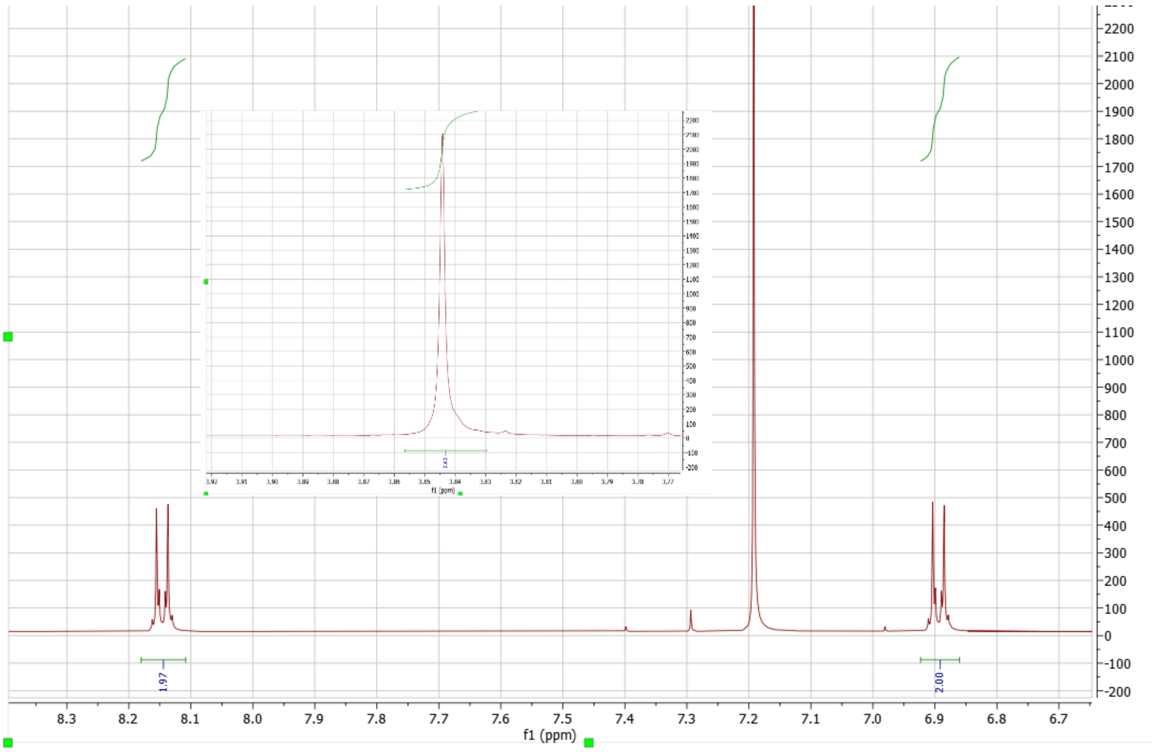

Figure S61. ^{1}H NMR spectrum of 1-methoxy-4-nitrobenzene (inset: high field peak).

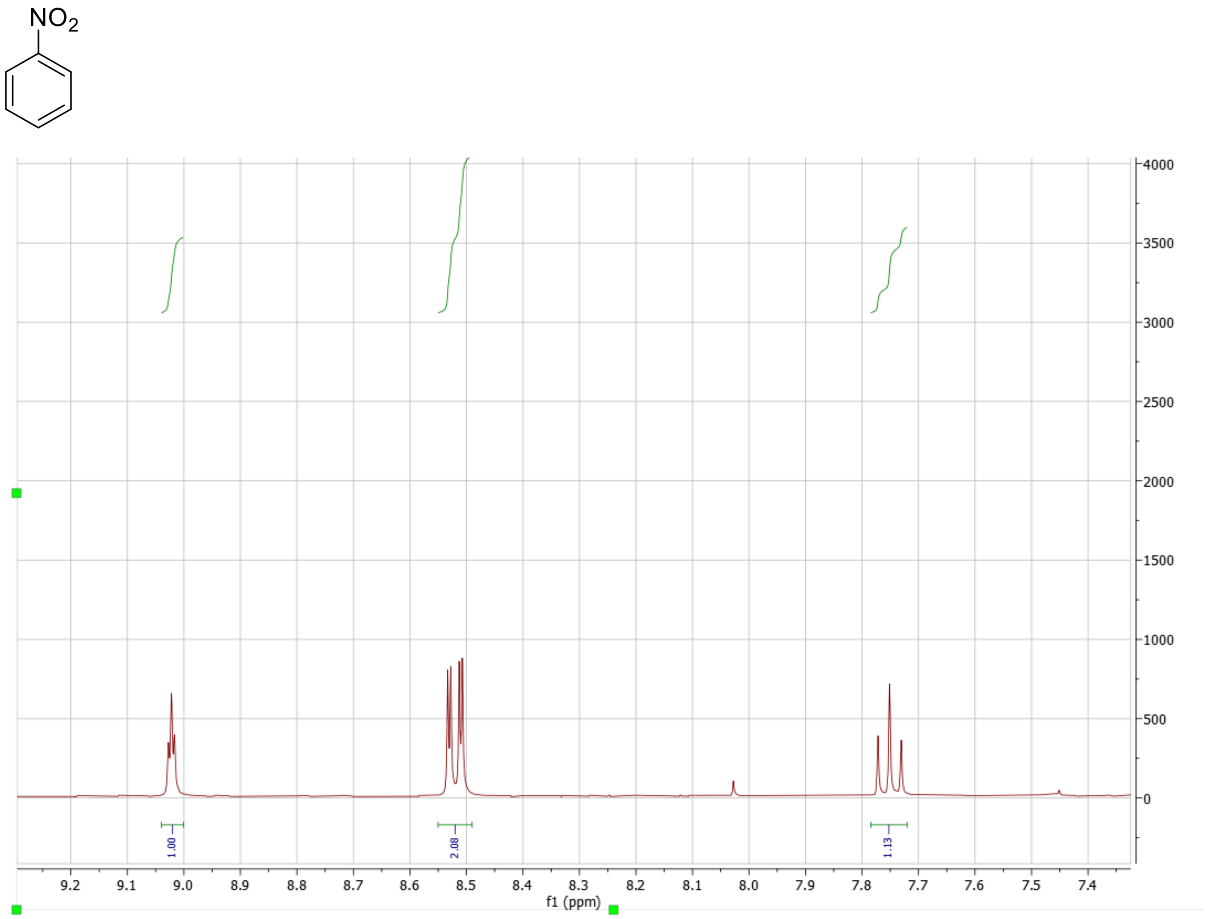

Figure S62. ^{1}H NMR spectrum of nitrobenzene.

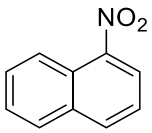

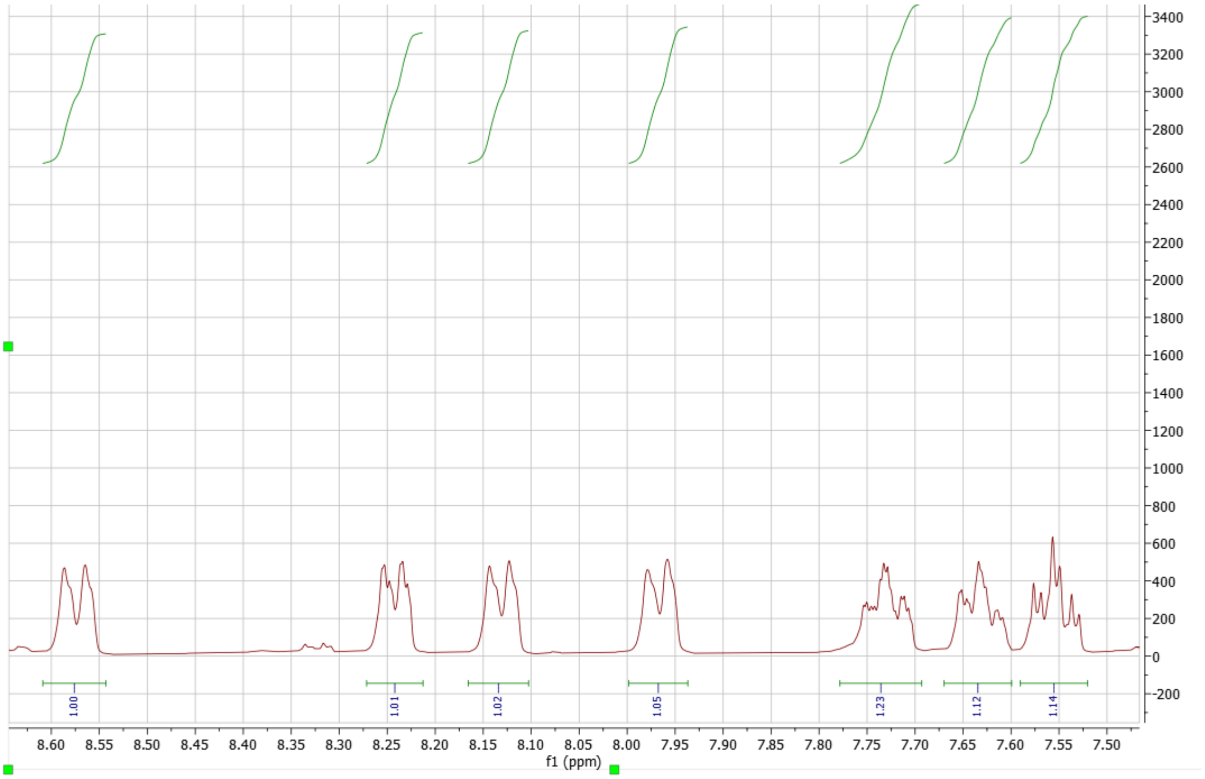

Figure S63. ^{1}H NMR spectrum of 1-nitronaphthalene.

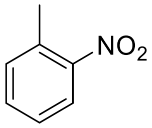

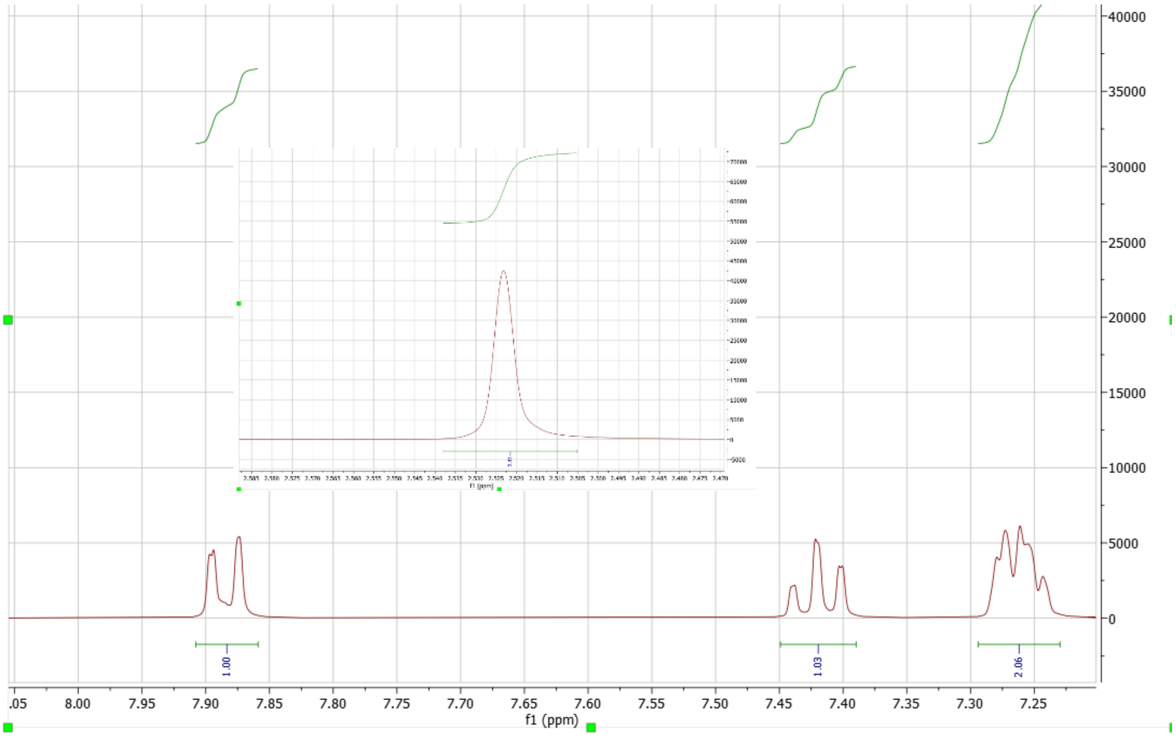

Figure S64. ^{1}H NMR spectrum of 2-nitrotoluene (inset: high field peak).

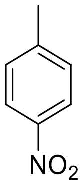

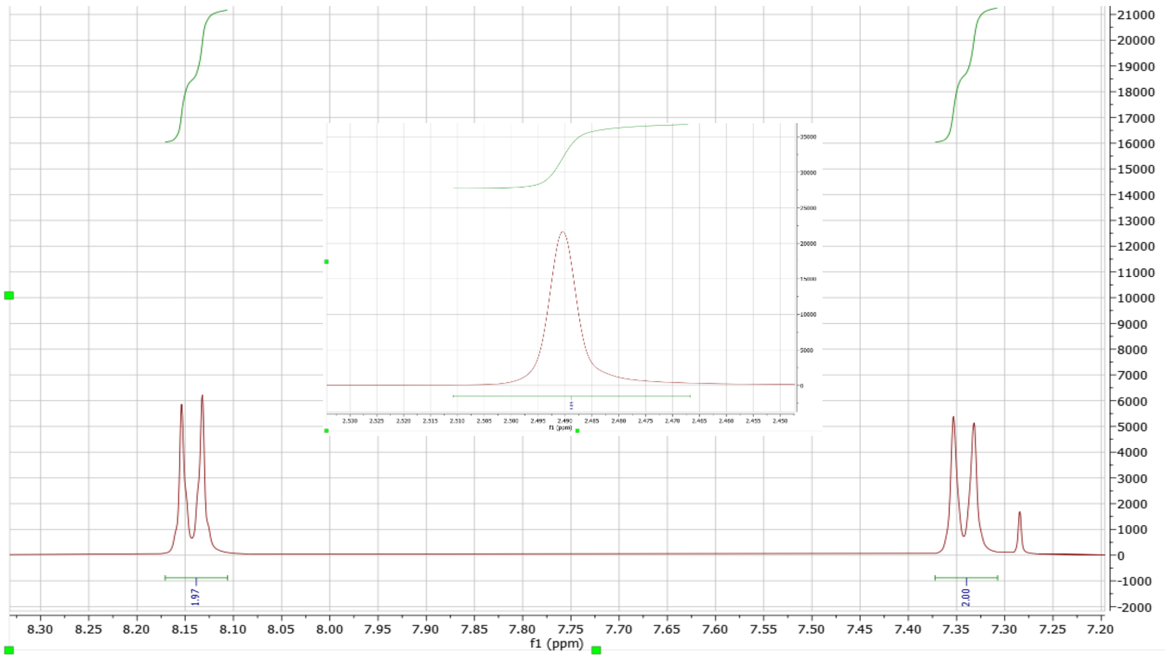

Figure S65. ^{1}H NMR spectrum of 4-nitrotoluene (inset: high field peak).

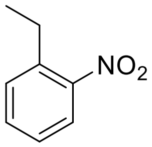

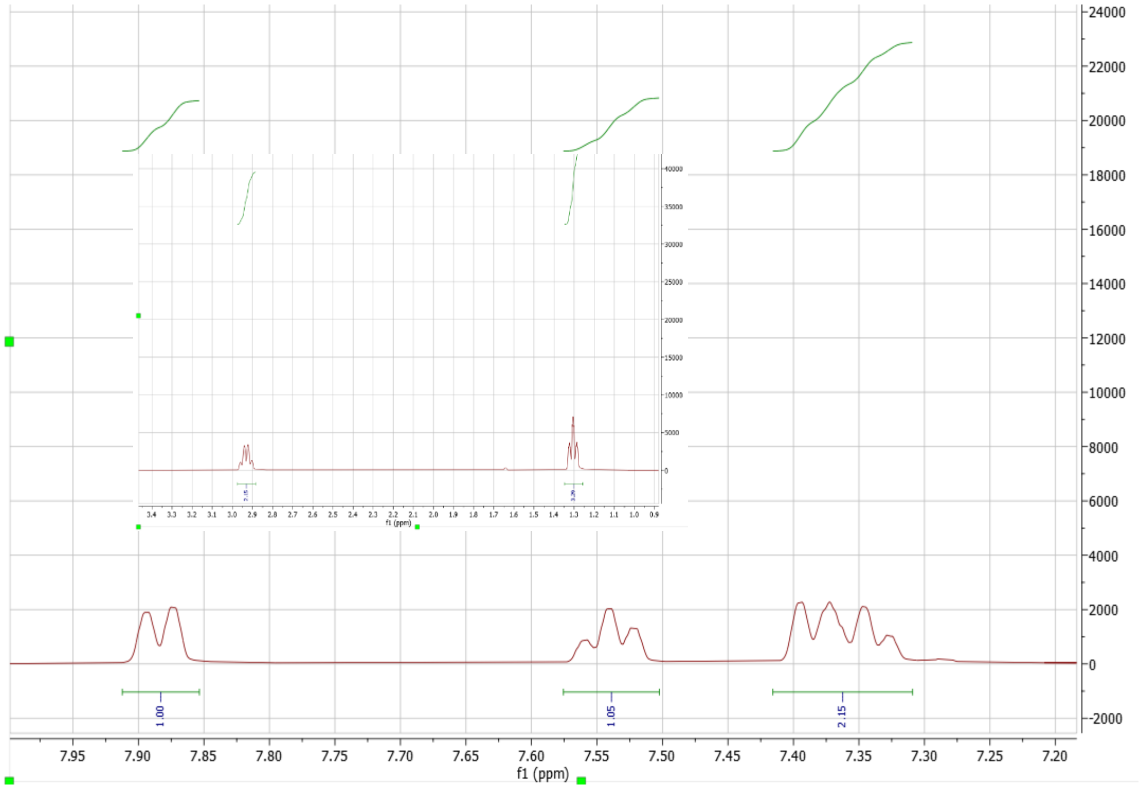

Figure S66. ¹H NMR spectrum of 1-ethyl-2-nitrobenzene (inset: high field peak).

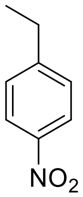

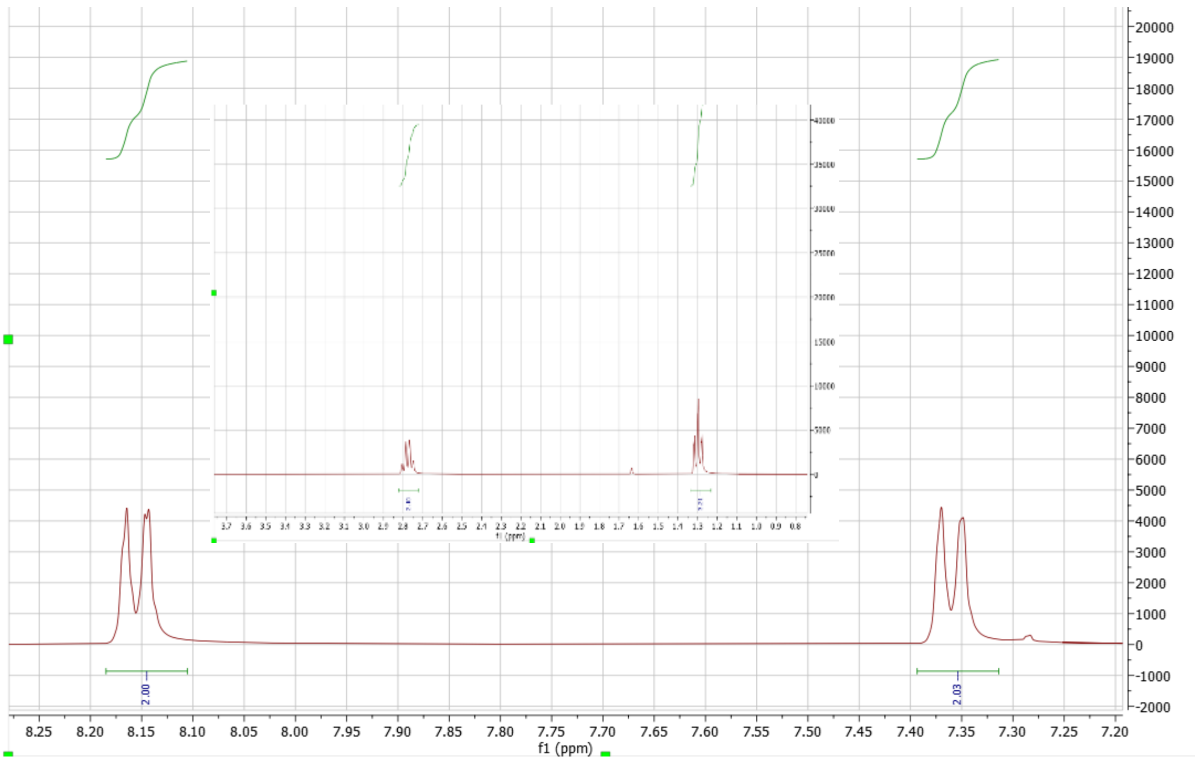

Figure S67. ^{1}H NMR spectrum of 1-ethyl-4-nitrobenzene (inset: high field peak).

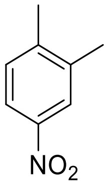

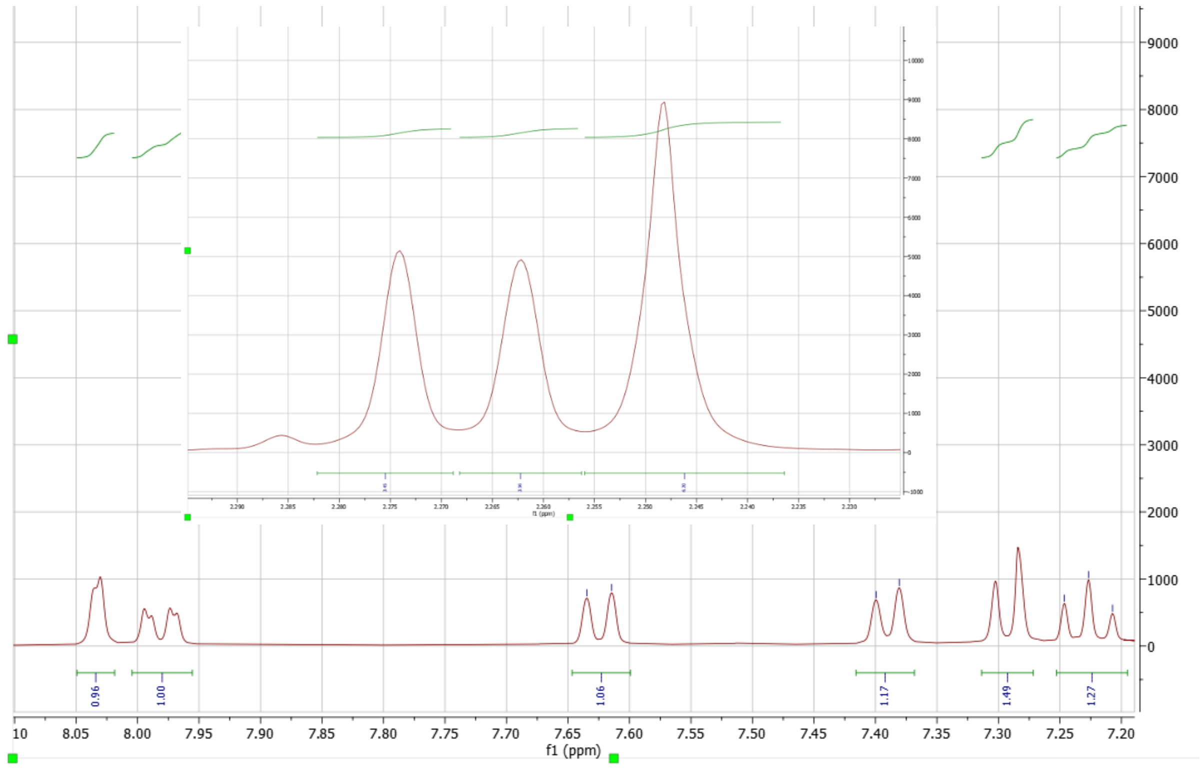

Figure S68. ^{1}H NMR spectrum of mixture of 3-nitro-*o*-xylene and 4-nitro-*o*-xylene (inset: high field peak).

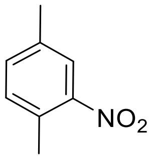

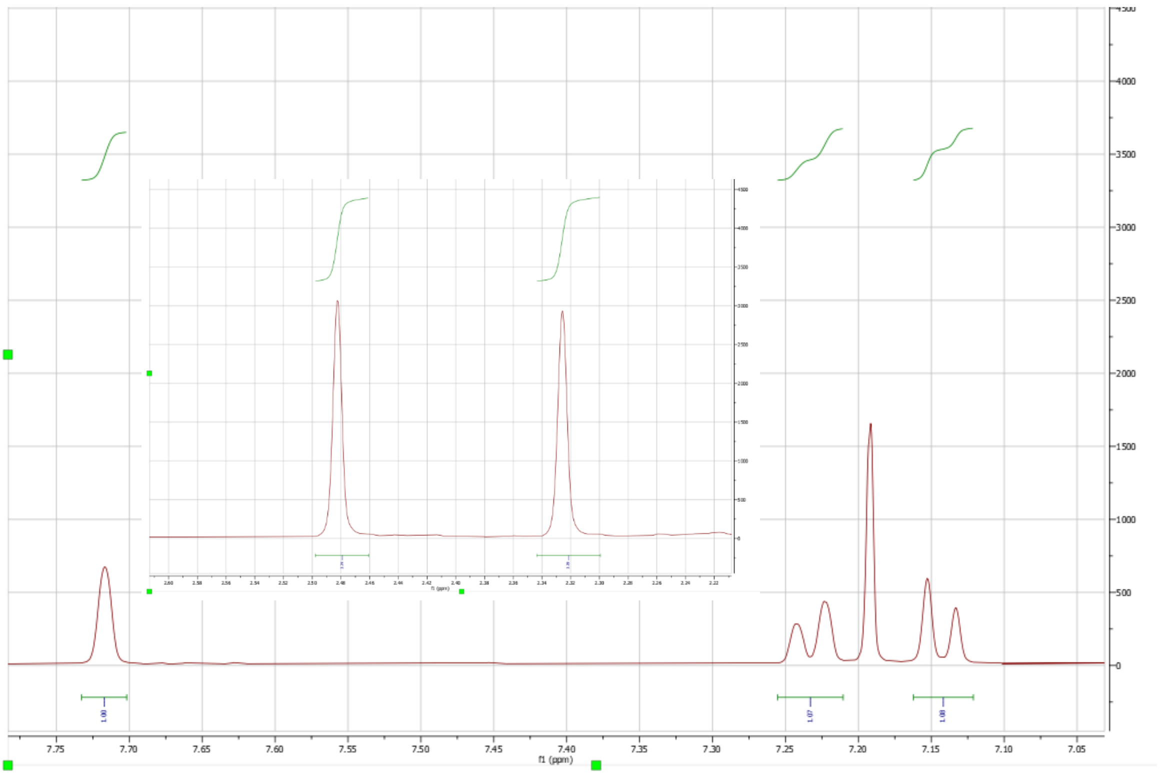

Figure S69. ^{1}H NMR spectrum of 2-nitro-*p*-xylene (inset: high field peak).

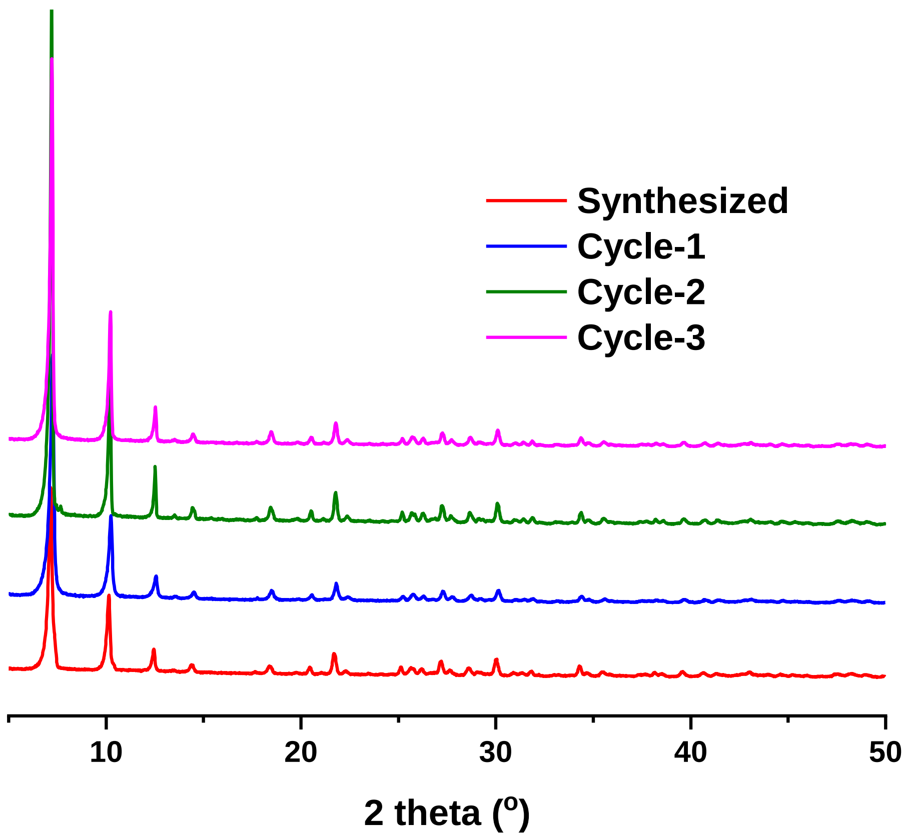

Figure S70. PXRD patterns of Zr-bptc samples after cycles of conversion of captured NO_2 to nitrobenzene.

Table S6. Elemental analysis activated and recycled Zr-bptc samples[a]

	Zr(%)	C(%)	H(%)
Calculated	33.0	34.8	1.34
Activated	32.9	33.4	1.50
Cycle-1	33.2	34.4	1.44
Cycle-2	32.0	32.7	1.47
Cycle-3	33.9	35.4	1.53

a. The recycled sample was reactivated to remove solvent

10. References

1. Wang, H.; Dong, X.; Lin, J.; Teat, J. S.; Jensen, S.; Cure, J.; Alexandrov, V. E.; Xia, Q.; Tan, K.; Wang, Q.; Olson, H. D.; Proserpio, M. D.; Chabal, J. Y.; Thonhauser, T.; Sun, J.; Han, Y.; Li, J. Topologically guided tuning of Zr-MOF pore struc-tures for highly selective separation of C6 alkane isomers. *Nat. Commun.* **2018**, 9, 1745.
2. Han, X.; Godfrey, G. W. H.; Briggs, L.; Davies, J. A.; Cheng, Y.; Daemen, L. L.; Sheveleva, M. A.; Tuna, F.; McInnes, J. L. E.; Sun, J.; Drathen, C.; George, W. M.; Ramirez-Cuesta, J. A.; Thomas, M. K.; Yang, S.; Schröder, M. Reversible adsorption of nitrogen dioxide within a robust porous metal–organic framework. *Nat. Mater.* **2018**, 17, 691–696.
3. Li, J.; Han, X.; Zhang, X.; Sheveleva, M. A.; Cheng, Y.; Tuna, F.; McInnes, J. L. E.; McPherson, J. M. L.; Teat, J. S.; Daemen, L. L.; Ramirez-Cuesta, J. A.; Schröder, M.; Yang, S. Capture of nitrogen dioxide and conversion to nitric acid in a porous met-al–organic framework. *Nat. Chem.* **2019**, 11, 1085–1090.
4. Hutter, J.; Iannuzzi, M.; Schiffmann, F.; VandeVondele, J. CP2K: atomistic simulations of condensed matter systems. *Wiley Interdiscip. Rev. Comput. Mol. Sci.* **2014**, *4*, 15-25.
5. Lippert, G.; Hutter, J.; Parrinello, M. A hybrid Gaussian and plane wave density functional scheme. *Mol. Phys.* **1997**, *92*, 477–487.
6. VandeVondele, J.; Krack, M.; Mohamed, F.; Parrinello, M.; Chassaing, T.; Hutter, J. Quickstep: Fast and accurate density functional calculations using a mixed Gaussian and plane waves approach. *Comput. Phys. Commun.* **2005**, *167*, 103–128.
7. VandeVondele, J.; Hutter. J. Gaussian basis sets for accurate calculations on molecular systems in gas and condensed phases. *J. Chem. Phys.* **2007**, *127*, 114105.
8. Goedecker, S.; Teter, M.; Hutter. J. Separable dual-space Gaussian pseudopotentials. *Phys. Rev. B* **1996**, *54*, 1703-1710.
9. Perdew, J. P.; Burke, K.; Ernzerhof, M. Generalized gradient approximation made simple. *Phys. Rev. Lett.* **1996**, *77*, 3865–3868.
10. Grimme, S.; Antony, J.; Ehrlich, S.; Krieg, H. A consistent and accurate *ab* initio parametrization of density functional dispersion correction (DFT-D) for the 94 elements H-Pu. *J. Chem. Phys.* **2010**, *132*, 154104.
11. Cheng, Y.; Daemen, L. L.; Kolesnikov, A. I.; Ramirez-Cuesta, A. J. Simulation of Inelastic Neutron Scattering Spectra Using OCLIMAX. *J. Chem. Theory Comput.* **2019**, *15*, 1974–1982.
12. Stoll, S.; Schweiger, A. EasySpin, a comprehensive software package for spectral simulation and analysis in EPR. *J. Magn. Reson.* **2006**, *178*, 42–55.
13. Han, X.; Hong, Y.; Ma, Y.; Lu, W.; Li, J.; Lin, L.; Sheveleva, A. M.; Tuna, F.; McInnes, J. L. E.; Dejoie, C.; Sun, J.; Yang, S.; Schröder, M. Adsorption of nitrogen dioxide in a redox-active vanadium metal-organic framework material. *J. Am. Chem. Soc.* **2020**, *142*, 15235−15239.
14. Levasseur, B.; Petit, C.; Bandosz, T. J. Reactive adsorption of NO_2 on copper-based metal-organic framework and graphite oxide/metal-organic framework composites. *ACS Appl. Mater. Interfaces* **2010**, *2*, 3606–3613.
15. Ebrahim, A. M.; Levasseur, B.; Bandosz, T. J. Interactions of NO_2 with Zr-based MOF: effects of the size of organic linkers on NO_2 adsorption at ambient conditions. *Langmuir* **2013**, *29*, 168–174.
16. Ebrahim, A. M.; Bandosz, T. J. Effect of amine modification on the properties of zirconium- carboxylic acid based materials and their applications as NO_2 adsorbents at ambient conditions. *Micropor. Mesopor. Mater.* **2014**, *188*, 149–162.
17. DeCoste, J. B.; Demasky, T. J.; Katz, M. J.; Farha, O. K.; Hupp, J. T. A. UiO-66 analogue with uncoordinated carboxylic acids for the broad-spectrum removal of toxic chemicals. *New J. Chem.* **2015**, *39*, 2396–2399.
18. Peterson, G. W.; Mahle, J. J.; DeCoste, J. B.; Gordon, W. O.; Rossin, J. A. Extraordinary NO_2 removal by the metal-organic framework UiO-66-NH_2. *Angew. Chem. Int. Ed.* **2016**, *55*, 6235–6238.
19. Ebrahim, A. M.; Bandosz, T. J. $Ce^{(III)}$ doped Zr-based MOFs as excellent NO_2 adsorbents at ambient conditions. *ACS Appl. Mater. Interfaces* **2013**, *5*, 10565–10573.

20. Ebrahim A. M.; Bandosz, T. J. Effect of amine type on acidic toxic gas adsorption at ambient conditions on modified CuBTC. *J. Environ. Chem. Eng.* **2022**, *10*, 107261.
21. Wang, X.; Su, R.; Zhao, Y.; Guo, W.; Gao, S.; Li, K.; Liang, G.; Luan, Z.; Li, L.; Xi, H.; Zou, R. Enhanced adsorption and mass transfer of hierarchically porous Zr-MOF nanoarchitectures towards toxic chemical removal. *ACS Appl. Mater. Interfaces* **2021**, *13*, 58848−58861.
